# Culture-free genome-wide locus sequence typing (GLST) provides new perspectives on *Trypanosoma cruzi* dispersal and infection complexity

**DOI:** 10.1371/journal.pgen.1009170

**Published:** 2020-12-16

**Authors:** Philipp Schwabl, Jalil Maiguashca Sánchez, Jaime A. Costales, Sofía Ocaña-Mayorga, Maikell Segovia, Hernán J. Carrasco, Carolina Hernández, Juan David Ramírez, Michael D. Lewis, Mario J. Grijalva, Martin S. Llewellyn

**Affiliations:** 1 Institute of Biodiversity, Animal Health & Comparative Medicine, University of Glasgow, Glasgow, United Kingdom; 2 Centro de Investigación para la Salud en América Latina, Pontificia Universidad Católica del Ecuador, Quito, Ecuador; 3 Laboratorio de Biología Molecular de Protozoarios, Instituto de Medicina Tropical, Universidad Central de Venezuela, Caracas, Venezuela; 4 Grupo de Investigaciones Microbiológicas-UR (GIMUR), Departamento de Biología, Facultad de Ciencias Naturales, Universidad del Rosario, Bogotá, Colombia; 5 London School of Hygiene & Tropical Medicine, Keppel Street, London, United Kingdom; 6 Infectious and Tropical Disease Institute, Biomedical Sciences Department, Heritage College of Osteopathic Medicine, Ohio University, Athens, OH, United States of America; University of Pennsylvania, UNITED STATES

## Abstract

Analysis of genetic polymorphism is a powerful tool for epidemiological surveillance and research. Powerful inference from pathogen genetic variation, however, is often restrained by limited access to representative target DNA, especially in the study of obligate parasitic species for which *ex vivo* culture is resource-intensive or bias-prone. Modern sequence capture methods enable pathogen genetic variation to be analyzed directly from host/vector material but are often too complex and expensive for resource-poor settings where infectious diseases prevail. This study proposes a simple, cost-effective ‘genome-wide locus sequence typing’ (GLST) tool based on massive parallel amplification of information hotspots throughout the target pathogen genome. The multiplexed polymerase chain reaction amplifies hundreds of different, user-defined genetic targets in a single reaction tube, and subsequent agarose gel-based clean-up and barcoding completes library preparation at under 4 USD per sample. Our study generates a flexible GLST primer panel design workflow for *Trypanosoma cruzi*, the parasitic agent of Chagas disease. We successfully apply our 203-target GLST panel to direct, culture-free metagenomic extracts from triatomine vectors containing a minimum of 3.69 pg/μl *T*. *cruzi* DNA and further elaborate on method performance by sequencing GLST libraries from *T*. *cruzi* reference clones representing discrete typing units (DTUs) TcI, TcIII, TcIV, TcV and TcVI. The 780 SNP sites we identify in the sample set repeatably distinguish parasites infecting sympatric vectors and detect correlations between genetic and geographic distances at regional (< 150 km) as well as continental scales. The markers also clearly separate TcI, TcIII, TcIV and TcV + TcVI and appear to distinguish multiclonal infections within TcI. We discuss the advantages, limitations and prospects of our method across a spectrum of epidemiological research.

## Author summary

This study details a rapid and cost-effective amplicon sequencing-based approach to measuring genome-wide DNA polymorphism in pathogenic microorganisms. Library preparation is completed in two simple polymerase chain reactions and thus avoids significant costs and biases of cell purification and culturing procedures typically involved prior to the sequencing of obligate parasite genomes. An emphasis on reaction multiplexability during primer panel design enables efficient genome-wide target amplification directly from infection source DNA. We provide proof-of-principle by genotyping hundreds of single-nucleotide polymorphisms in the Chagas disease agent *Trypanosoma cruzi* using metagenomic DNA extracts from infected triatomine (kissing bug) intestinal material collected in Colombia, Venezuela and Ecuador. We also evaluate method performance using reference clone DNA. Results distinguish *T*. *cruzi* population structure and diversity patterns from within-city to cross-country scales and recapitulate ancestral relationships among the sub-lineages TcI, TcIII, TcIV, TcV and TcVI. Unbalanced alternate allele frequency distributions repeatedly measured in a subset of samples also suggest potential to distinguish co-infection by multiple TcI strains. We discuss further applications as well as possibilities for method refinement.

## Introduction

Genome-wide single nucleotide polymorphism (SNP) analysis is a powerful and increasingly common approach in the study and surveillance of infectious disease. Understanding patterns of SNP diversity within pathogen genomes and across pathogen populations can resolve fundamental biological questions (e.g., reproductive mechanisms in *T*. *cruzi* [1]), reconstruct past [2] and present transmission networks (e.g., *Staphylococcus* infections within hospitals [3]) or identify the genetic bases of virulence [4,5] and resistance to drugs (see examples from *Plasmodium* spp. [6,7]). A number of obstacles, however, complicate access to representative, genome-wide SNP information using modern sequencing tools. Pathogens are often sampled in low quantities and together with large amounts of host/vector tissue, microbiota or environmental DNA. Sequencing is rarely viable directly from the infection source and studies have often found it necessary to isolate and culture the target organism to higher densities before extracting DNA. These additional steps, however, are resource-intensive and bias-prone. Pathogen isolation is less often attempted on asymptomatic infections and is less likely to succeed when levels of parasitaemia in a sample are low. Genomic sequencing data on the protozoan parasite *Leishmania infantum*, for example, has for such reasons come to exhibit considerable selection bias towards aggressive strains isolated by invasive sampling from canine hosts. Vector-isolated genomes have yet to be reported from the Americas and only a single study claims to have sequenced *L*. *infantum* from asymptomatic hosts [8]. Selection bias also often occurs due to competition among isolated strains. Studies on the related, Chagas disease parasite *Trypanosoma cruzi*, for example, are time and again confounded by growth and survival rate differences among genotypes in culture [9–11], with gradual reductions in genetic diversity often observed over time [12]. Karyotypic changes also arise during *T*. *cruzi* micromanipulation and axenic growth [13,14]. These effects in culture have confounded efforts to associate genetic variability and sub-lineage taxonomy to important clinical and eco-epidemiological traits (see further below) [15].

A variety of approaches therefore aim to obtain genome-wide SNP information without first performing pathogen isolation and culturing steps. Some studies separate target sequences from total DNA or RNA by exploiting base modifications or transcriptional properties specific to the pathogen [16], vector [17] or host [18,19]. Others describe the use of biotinylated hybridization probes [20–23] or selective whole-genome amplification, for example, based on the strand displacement function of phi29 DNA polymerase [24]. Such techniques are costly and often excessive when a study’s primary objective is to evaluate genetic distances and diversity among samples rather than to reconstruct complete haplotypes or investigate structural genetic traits. Epidemiological tracking, (sub-) lineage typing and source attribution studies, for example, often benefit little from measuring large invariant sequence areas or defining the complete architecture of sample genomes. It is nevertheless quite common to see such studies undertake expensive WGS procedures only for final analyses to take place ‘post-VCF’ [25], i.e., using a list of diagnostic markers compiled from a small fraction of polymorphic reads.

Highly multiplexed polymerase chain reaction (PCR) amplicon sequencing offers an efficient alternative when obtaining genome-wide SNP information is the primary goal. First marketed under the name Ion AmpliSeq by Thermo Fisher Scientific [26], the method consists in the simultaneous amplification of dozens to hundreds of DNA targets known or hypothesized to contain sequence polymorphism in the sample set. Each sample’s resultant amplicon pool is then prepared for sequencing by index/adaptor ligation or in a subsequent ‘barcoding’ PCR. Panel construction is highly flexible, requiring only that the primers exhibit similar melting/annealing temperatures and a low propensity to cross-react. As such, target selection can be tailored to specific research goals, for example, to profile resistance markers [27] or to genotype neutral SNP variation for landscape genetic techniques [28]. The potential to isolate and genotype pathogen DNA at high-resolution directly from uncultured sample types by multiplexed amplicon sequencing has however received little attention thus far. Simultaneous PCR-based detection of multiple pathogen species or genotypes is certainly common [29], but multiplexable primer panels are rarely designed for subsequent sequencing and polymorphism analysis. The Ion AmpliSeq brand currently offers pre-designed panels for studies on ebola [30] and tuberculosis [31] but the use of custom panels for other pathogen species (e.g., *Bifidobacterium* [32] or human papilloma virus [33]) remains surprisingly rare in the literature.

The present work describes the design and implementation of a large multiplexable primer panel for *T*. *cruzi* [34], a zoonotic parasite endemic to many tropical and subtropical areas of the American continent. *T*. *cruzi* is transmitted through the contact of abraded skin or mucosa with the feces of blood-sucking reduviid insects called triatomines. Congenital transmission and infection via contaminated food, blood or organ donations can also occur. While human infection often remains asymptomatic, 30–40% of cases involve life-threatening cardiovascular and/or gastrointestinal syndromes. This extensive clinical variability is loosely associated to genetic differences within and among the parasite’s six major sub-lineages, known as ‘discrete typing units’ (DTUs) TcI–TcVI [15]. TcI is the most widespread and genetically diverse DTU [35]. Previously considered less pathogenic than other DTUs during chronic stages of infection, it has become increasingly associated with severe chronic cardiomyopathy in areas North of the Amazon [15]. TcII, TcV and TcVI appear to predominate in central and southern South America [35], where infections causing megacolon and megaesophagus are more frequently observed [15]. TcIII and TcIV are rarely detected in domestic cycles although TcIV has been implicated in several food-borne outbreaks in Venezuela and Brazil [36,37]. Accessible, high-resolution genetic profiling methods are essential for a better understanding of these associations and other important *T*. *cruzi* traits.

In contrast to past multi-locus sequence typing (MLST) methods involving at most a few dozen (individually amplified) gene fragments [38], our ‘genome-wide locus typing’ (GLST) tool simultaneously amplifies 203 sequence targets across 33 (of 47) *T*. *cruzi* chromosomes. We apply GLST to metagenomic DNA extracts from TcI-infected triatomine vectors collected in Colombia, Venezuela and Ecuador and further describe method sensitivity/specificity by sequencing GLST libraries for *T*. *cruzi* clones representing TcI, TcIII, TcIV, TcV and TcVI. The 780 SNP sites identified via GLST repeatably distinguish parasites infecting sympatric vectors and detect correlations between genetic and geographic distances at regional (< 150 km) and continental scales. The markers also clearly separate TcI, TcIII, TcIV and TcV + TcVI and appear to distinguish multiclonal infections within TcI. We discuss advantages and limitations of our method for epidemiological studies in resource-poor settings where Chagas disease and other ‘neglected tropical diseases’ prevail.

## Methods

### Ethics statement

Triatomine sampling occurred in accordance to guidelines set by Autoridad Nacional de Licencias Ambientales permit number 63257–2014 granted to Universidad del Rosario, Ministerio del Ambiente de Ecuador permit number MAE-DNB-CM-2015-0030 granted to Pontificia Universidad Católica del Ecuador and Ministerio del Poder Popular para Ciencia y Tecnología permit number CEC-IMT 19/2009 granted to Universidad Central de Venezuela.

### Triatomine samples and *T*. *cruzi* reference clones

TcI-infected intestinal tract and/or faeces samples of *Panstrongylus chinai* and *Rhodnius ecuadoriensis* were collected by the Centro de Investigación para la Salud en América Latina (CISeAL) in Loja Province, Ecuador, following protocols described in Grijalva *et al*. 2012 [39]. DNeasy Blood and Tissue Kit (Qiagen) was used to extract metagenomic DNA. TcI-infected intestinal material of *P*. *geniculatus*, *R*. *pallescens* and *R*. *prolixus* from northern Colombia was also collected in previous projects [40–42], likewise using DNeasy Blood and Tissue Kit to extract metagenomic DNA. TcI-infected *P*. *geniculatus* specimens from Caracas, Venezuela were collected by the citizen science triatomine collection program (http://www.chipo.chagas.ucv.ve/vista/index.php) at Universidad Central de Venezuela. This program has supported various epidemiological studies in the capital district [43–45]. DNA was extracted from the insect faeces by isopropanol precipitation. Geographic coordinates and ecotypes (domestic, peri-domestic or sylvatic) of the sequenced samples are provided in S1 Table.

*T*. *cruzi* epimastigote DNA from reference clones CHILE_C22 (TcI) ARMA18_CL1 (TcIII), SAIMIRI3_CL8 (TcIV), PARA7_CL3 (TcV), CHACO9_COL15 (TcVI) and CLBRENER (TcVI) was obtained from the London School of Hygiene & Tropical Medicine (LSHTM). DNA extractions at LSHTM followed Messenger *et al*. 2015 [46].

Uninfected *R*. *prolixus* gut tissue samples used for mock infections (see ‘Wet lab method development and library preparation’) were also provided by LSHTM. Insects were euthanized with CO_2_ and hindguts drawn into 5 volumes of RNAlater (Sigma-Aldrich) by pulling the abdominal apex toward the posterior with sterile watchmaker’s forceps.

*T*. *cruzi* TcI X10/1 Sylvio reference clone (‘TcI-Sylvio’) epimastigotes used for mock infections and various other stages of method development were obtained from CISeAL. Cryo-preserved cells were returned to log-phase growth in liver infusion tryptose (LIT) and quantified by hemocytometer before pelleting at 25,000 g. Pellets were washed twice in PBS and parasites killed by resuspension in 10 volumes of RNAlater. DNA from these *T*. *cruzi* cells (and their dilutions with preserved *R*. *prolixus* intestinal tissue) was extracted by isopropanol precipitation.

Isopropanol precipitation was also used to extract DNA from *T*. *cruzi* plate clone TBM_2795_CL2. This sample was previously analyzed by WGS [1] and served as a control for GLST method development in this study.

### GLST target and primer selection

We began our GLST sequence target selection process by screening single-nucleotide variants previously identified in *T*. *cruzi* populations from southern Ecuador [1]. Briefly, Schwabl *et al*. sequenced genomic DNA from 45 cloned and 14 non-cloned *T*. *cruzi* field isolates on the Illumina HiSeq 2500 platform and mapped resultant 125 nt reads to the TcI-Sylvio reference assembly using default settings in BWA-mem v0.7.3 [47]. Single-nucleotide polymorphisms (SNPs) were summarized by population-based genotype and likelihood assignment in Genome Analysis Toolkit v3.7.0 (GATK) [48], excluding sites with low cumulative call confidence (QUAL < 1,500) and/or aberrant read-depth (< 10 or > 100) as well as those belonging to clusters of three or more SNPs. A ‘virtual mappability’ mask [49] was also applied to avoid SNP inference in areas of high sequence redundancy in the *T*. *cruzi* genome. Read-mapping and variant exclusion criteria were verified by subjecting TcI-Sylvio Illumina reads from Franzen *et al*. 2012 [50] to the same pipelines as the Ecuadorian dataset. An additional mask was set around small insertion-deletions detected in these reads based on the assumption that the reference sample should not present alternate genotypes in high-quality contigs of the assembled genome.

We extracted 160 nt segments from the *T*. *cruzi* reference genome (.fasta file) whose internal sequence (positions 41 to 120) contained between one and ten of 75,038 SNPs identified in the above WGS dataset. These 56,428 segments were further filtered for orthology between *T*. *cruzi* and *Leishmania major* genomes as defined by the OrthoMCL algorithm [51] at https://tritrypdb.org. Such conserved segments may be least prone to repeat-driven nucleotide diversity and as such most amenable to PCR [52]. The 6,259 orthology segments found by OrthoMCL therefore proceeded to primer search with the high-throughput primer design engine BatchPrimer3 [53]. As target SNPs did not occur in the outer 40 nt of each orthology segment, these flanking regions provided additional flexibility to identify primers matching the criteria listed in Table 1.

**Table 1:** Primer selection criteria specified in BatchPrimer3.

Criterion	Value
minimum primer size	24 nt
maximum primer size	35 nt
optimal primer size	24 nt
minimum product size	120 nt
maximum product size	160 nt
optimal product size	120 nt
minimum melting temperature	63°C
maximum melting temperature	65°C
optimal melting temperature	63°C
maximum self-complementarity	4 nt
maximum 3’ self-complementarity	2 nt
maximum length of mononucleotide repeats	3 nt
minimum GC content	40%
maximum GC content	60%

**Fig 1:**
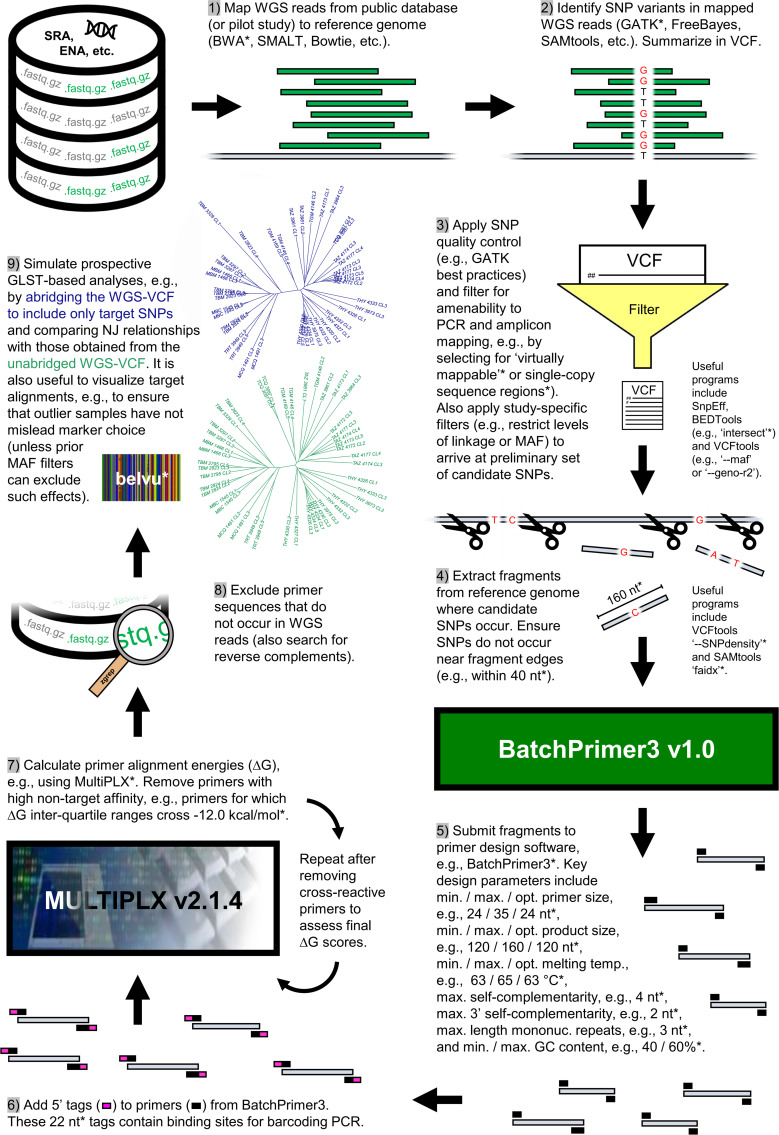
GLST sequence target selection from preliminary genomic data. Nine steps of primer panel construction and validation run clockwise from top left. Various methods and criteria can be applied to complete many of these steps. Those specific to this study are asterisked, e.g., we used BWA [47] in step 1 and GATK [48] in step 2. Abbreviations: SRA, Sequence Read Archive at www.ncbi.nlm.nih.gov/sra; ENA, European Nucleotide Database at www.ebi.ac.uk/ena; WGS, whole-genome sequencing; SNP, single-nucleotide polymorphism; MAF, minor allele frequency; PCR, polymerase chain reaction; VCF, variant call format; NJ, neighbor-joining.

Each of 286 forward primer candidates output by BatchPrimer3 received the additional 5’ tag sequence 5’-ACACTGACGACATGGTTCTACA-3’ and reverse primer candidates received the 5’ tag sequence 5’-TACGGTAGCAGAGACTTGGTCT-3’. These tag sequences enable single-end barcode and Illumina P5/P7 adaptor attachment in second-round PCR. Next, we determined binding energies (ΔG) for all possible primer-pairs using the primer compatibility software MultiPLX v2.1.4 [54]. We discarded primers with inter-quartile ranges crossing a threshold of ΔG = -12.0 kcal/mol. Primers with 20 or more interactions showing ΔG ≤ -12.0 kcal/mol were also disallowed. The remaining 248 primer-pairs (median ΔG = -9.0) underwent a last filtering step by screening for perfect matches in raw WGS sequence files (.fastq). Low match frequency led to the elimination of 45 additional primer pairs. WGS alignments corresponding to the 203 sequence regions targeted by this final primer set were visualized in Belvu v12.4.3 [55]. The 403 SNPs occurring within these sequence regions distributed evenly across individuals in Loja Province. Using the ‘nj’ function from the ‘ape’ package v5.0 [56] in R v3.4.1 [57], the 403 SNPs also reproduced neighbor-joining relationships observed based on total polymorphism identified by WGS (S1 Fig). These observations lent further support to the suitability of the GLST marker panel for the analysis of genetic differentiation at the landscape-scale. The GLST sequence target selection process described above is summarized in Fig 1.

### Wet lab method development and library preparation

The 203 primers pairs designed above (S2 Table) were purchased from Eurofins Genomics (Ebersberg, Germany) at 200 μM concentration in salt-free, 96-well plate format. Primer pairs were first tested individually to establish cycling conditions for PCR (S2 Fig). Optimal target amplification occurred with an initial incubation step at 98°C (2 min); 30 amplification cycles at 98°C (10 s), 60°C (30 s) and 72°C (45 s); and a final extension step at 72°C (2 min). The 10 μl reactions contained 5 μl Q5 High-Fidelity Master Mix (New England Biolabs), 1 μl forward primer [10 μM], 1 μl reverse primer [10 μM] and 3 μl TcI-Sylvio epimastigote DNA. The multiplexed, first-round ‘GLST’ PCR reaction was prepared by combining all 406 primers in equal proportions and diluting the combined mix to 50.75 μM, resulting in individual primer concentrations of 50.75 μM / 406 = 125 nM. GLST reactions incorporated 2 μl of this primer mix rather than two separate 1 μl forward/reverse primer inputs as above.

We first tested GLST PCR on DNA extracts from mock infections, each consisting of 10^4^, 10^5^ or 10^6^ TcI-Sylvio epimastigote cells and one uninfected *R*. *prolixus* intestinal tract (S3 Fig). Amplicons from lower concentration epimastigote dilutions gave weaker signals in gel electrophoresis, suggesting lower infection load thresholds at which vector gut DNA becomes unsuitable for GLST. Most vector gut DNA extracts obtained for this study represented donated material of limited quality and infection load, some also without signal in PCR spot tests for the presence of high frequency ‘TcZ’ [58] satellite DNA (commonly targeted to diagnose human *T*. *cruzi* infections).

We therefore first used qPCR to identify vector gut samples containing *T*. *cruzi* DNA quantities within ranges successfully visualized from GLST reactions on epimastigote DNA quantified by Qubit fluorometry (Invitrogen) and serially diluted from 1.35 ng/μl to 2.50 pg/μl in dH_2_O (S4 Fig). Each 20 μl qPCR reaction consisted of 10 μl SensiMix SYBR Low-ROX reagent (Bioline), 1 μl TcZ [58] forward primer (5’-GCTCTTGCCCACAMGGGTGC-3’) [10 μM], 1 μl TcZ [58] reverse primer (5’-CCAAGCAGCGGATAGTTCAGG-3’) [10 μM], 7 μl dH_2_O and 1 μl vector gut DNA. Samples were amplified together with a 15-step standard curve containing between 0.30 pg and 4.82 ng *T*. *cruzi* epimastigote DNA. Reaction conditions consisted of an initial incubation step at 95°C (10 min) and 40 amplification cycles at 95°C (15 s), 55°C (15 s) and 72°C (15 s). Fluorescence acquisition occurred at the end of each cycle and final product dissociation was measured in 0.5°C increments between 55 and 95°C.

Vector gut samples suggested to contain at least 1.0 pg/μl *T*. *cruzi* concentrations based on qPCR proceeded to final library construction (S1 Table) alongside DNA from *T*. *cruzi* clones TBM_2795_CL2 (TcI), CHILE_C22 (TcI) ARMA18_CL1 (TcIII), SAIMIRI3_CL8 (TcIV), PARA7_CL3 (TcV), CHACO9_COL15 (TcVI) and CLBRENER (TcVI). Several samples were processed in 2–4 replicates beginning with the first-round GLST PCR reaction step. First-round PCR products were electrophoresed in 0.8% agarose gel to separate target bands (mode = 164 nt) from primer polymers quantified with the Agilent Bioanalyzer 2100 System (see 78 nt primer peak in S5 Fig). Excised target bands were re-solubilized with the PureLink Quick Gel Extraction Kit (Invitrogen) to create input for subsequent barcoding PCR. This second PCR reaction consisted of an initial incubation step at 98°C (2 min); 7 amplification cycles at 98°C (30 s), 60°C (30 s) and 72°C (1 min); and a final extension step at 72°C (3 min). Only 7 amplification cycles were used given polymer ‘daisy-chaining’ observed when cycling at 13 and 18x (S6 Fig). The barcoding reaction adds Illumina flow cell and sequencing primer binding sites to each first-round PCR product. A different reverse primer is used for each sample. The reverse primer (5’-CAAGCAGAAGACGGCATACGAGAT*X*TACGGTAGCAGAGACTTGGTCT-3’) contains a 10 nt barcode (*X*) to distinguish reads from different samples during pooled sequencing. It also contains CS2 (sequencing primer binding sites). A single forward primer (5'-AATGATACGGCGACCACCGAGATCTACACTGACGACATGGTTCTA-3') containing CS1 is used for all samples. Each 20 μl barcoding reaction contained 10 μl Q5 High-Fidelity Master Mix (New England Biolabs), 0.8 μl forward (universal) primer [10 μM], 0.8 μl (barcoded) reverse primer [10 μM], 5.4 μl dH_2_O and 3 μl (gel-purified) first-round PCR product. Barcoding primers were purchased from Eurofins Genomics at 100 μM concentration in HPLC-purified, 96-well plate format. Barcoded amplicons (e.g., S7 Fig) were quantified by Qubit fluorometry (Thermo Fisher Scientific), pooled at equimolar concentrations, gel-excised, re-solubilized and verified by microfluidic electrophoresis (S8 Fig) as above.

### GLST amplicon sequencing and variant discovery

The GLST pool was sequenced twice on an Illumina MiSeq instrument. We first used the pool to ‘spike’ additional base diversity into a collaborator’s 16S amplicon sequencing run. 16S samples were loaded to achieve 80% sequence output whereas GLST and PhiX DNA were each loaded at 10%. This first run occurred in 500-cycle format using MiSeq Reagent Kit v2. The second run occurred in 300-cycle format using MiSeq Reagent Micro Kit v2 and was dedicated solely to GLST (also no PhiX DNA). Both runs were performed at Glasgow Polyomics using Fluidigm Access Array sequencing primers FL1 (CS1 + CS2) and CS2rc [59].

Demultiplexed sequence reads were trimmed to 120 nt and mapped to the TcI-Sylvio reference assembly using default settings in BWA-mem v0.7.3 [47]. Mapped reads with poor alignment scores (AS < 100) were discarded to decontaminate samples of non-*T*.*cruzi* sequences sharing barcodes with the GLST dataset. Identical results were achieved using BWA-sw in DeconSeq v0.4.3 [60] to decontaminate reads. After merging alignment (.bam) files from sequencing runs 1 and 2, SNPs were identified in each sample using the ‘HaplotypeCaller’ algorithm in GATK v3.7.0 [48]. Population-based genotype and likelihood assignment followed using ‘GenotypeGVCFs’. We excluded SNP sites with QUAL < 80, D < 10, mapping quality (MQ) < 80 and or Fisher strand bias (FS) > 10. Individual genotypes were set to missing (./.) if they contained < 10 reads and set to reference (0/0) if they contained only a single alternate read (i.e., if they were classified as heterozygotes based on minor allele frequencies ≤ 10%). These filtering thresholds were cleared by all expected SNPs (i.e., SNPs also found in prior WGS sequencing) but not by all new SNPs found using GLST (e.g., see comparison of QUAL density curves in S9 Fig). SNP calling with GATK [48] was also performed separately for sequencing runs 1 and 2 in order to exclude SNP sites uncommon to both analyses from the merged dataset described above.

### GLST repeatability, population genetic and spatial analyses

A phylogenetic tree was built from the filtered SNP dataset by counting the number of non-reference alleles (0, 1 or 2) in each genotype at all biallelic sites with the VCFtools v0.1.13 [61] function ‘--012’, summing pairwise Euclidean distances and plotting neighbor-joining relationships with the ‘nj’ function from the ‘ape’ package v5.0 [56] in R v3.4.1[57]. Only sites with genotypes called in all individuals (i.e., ‘non-missing sites’) were included in analysis.

Genetic differences at non-missing sites were also visualized as a median-joining network, i.e., a minimum spanning tree composed of observed sequences and unobserved (reconstructed) sequence nodes [62]. In order to account for both biallelic and polyallelic sites, we first created a multi-SNP alignment by applying the ‘vcf-to-tab’ script from VCFtools v0.1.13 [61] and concatenating each sample’s output fields. For example, genotypes ‘A/C’, ‘A/T’ and ‘G/G’ (ordered by genomic position) become ‘ACATGG’ for sample X. Mismatching alignment positions were then counted for each sample pair in the network construction program PopART v1.7 [63]. For biallelic sites, the distance calculated between two samples using this unphased alignment method is equivalent to that obtained by recoding all genotypes to non-reference allele counts and summing absolute differences (i.e., 0, 1 or 2 per site) as in neighbor-joining construction above. For polyallelic sites, the method allows for genotypes with equivalent alternate allele counts but distinct allelic identities to be distinguished. For example, if the reference allele is ‘A’ and sample X’s genotype ‘A/C’ is compared with sample Y’s genotype ‘A/G’, the difference between X and Y is 1. If sample Z’s genotype is ‘C/C’, the difference between X and Z is 1 and the difference between Y and Z is 2.

Linkage and neutrality statistics were calculated using VCFtools [61] functions ‘--geno-r2’ (calculates correlation coefficients between genotypes following Purcell *et al*. 2007 [64]), ‘--het’ (calculates inbreeding coefficients using a method of moments [65]) and ‘--hwe’ (filters sites by deviation from Hardy-Weinberg Equilibrium following Wigginton *et al*. 2005 [66]). F_ST_ differentiation was calculated using ARLSUMSTAT v3.5.2 [67]. These calculations considered only the first replicate of individuals present in multiple replicates.

Correlations between geographic and genetic differences among samples from Colombia, Venezuela and Ecuador were measured using a Euclidean genetic distance matrix calculated from non-reference allele counts at biallelic sites as described for neighbor-joining construction above. The ‘mantel’ function from the ‘vegan’ package v2.4.4 [68] in R v3.4.1 [57] was used to test significance of the Mantel statistic by permuting geographic distances and re-measuring correlations to genetic distances 999 times. SNP sites in which genotypes were missing in > 10% individuals were excluded from analysis. Replicates 2–4 were also excluded as before. Geographic distances were measured by projecting sample latitude/longitude (WGS 84) coordinates into a common xy plane (EPSG code 3786) selected following Šavrič *et al*. 2016 [69] (S1 Table).

The decision to exclude SNP sites with missing genotypes from several analyses initially led to significant information loss due to the presence of two outlier samples, ARMA18_CL1_rep2 and COL253, libraries of which had been sequenced despite poor target visibility in gel electrophoresis (i.e., final PCR product banding appeared similar to that of ECU2 in S7 Fig). Read-depths for the two samples averaged 1.2 interquartile ranges below the sample set median and precluded genotype assignment at > 25% SNP sites. We therefore excluded them from all analyses.

## Results

### SNP polymorphism and repeatability

**Fig 2:**
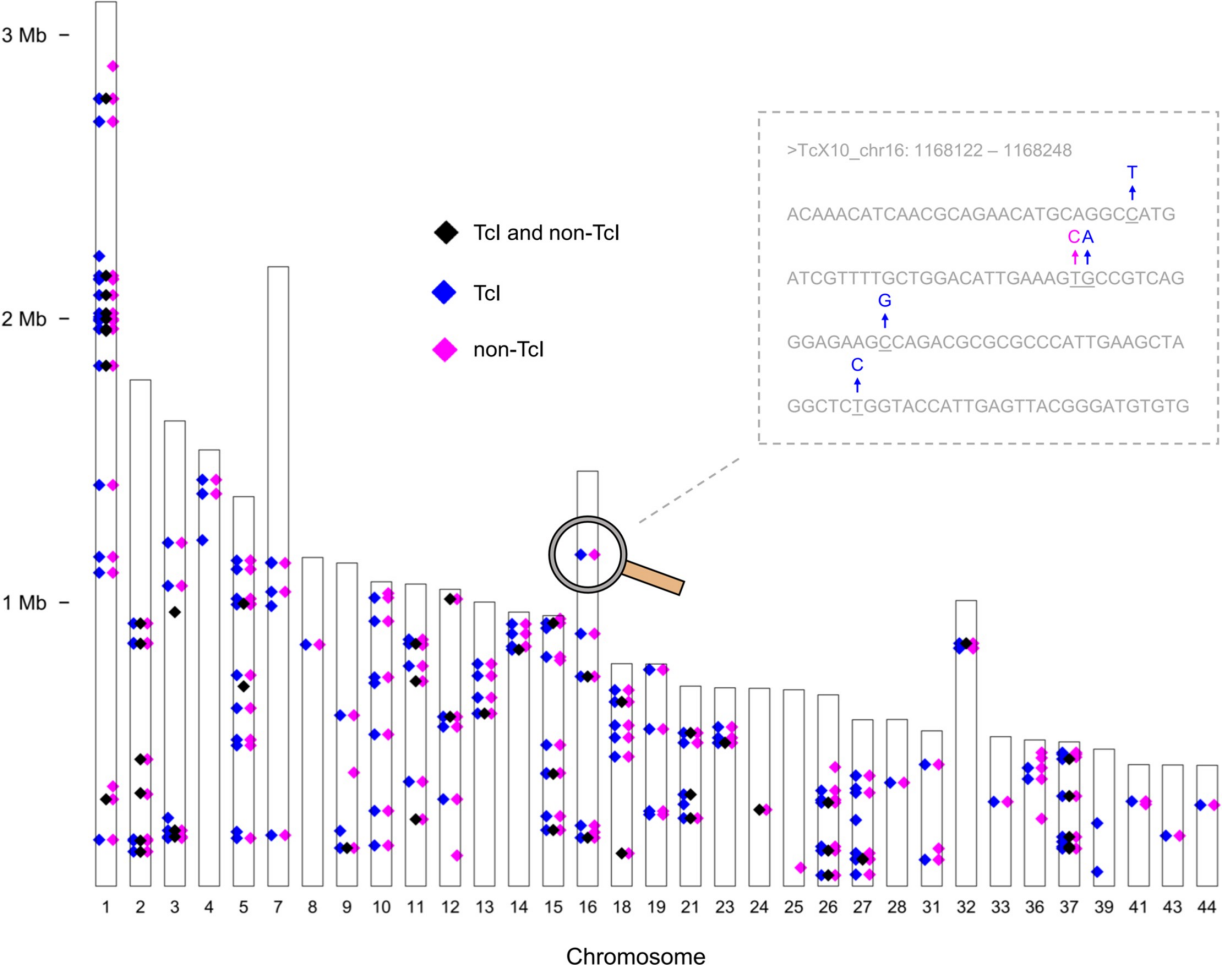
Variant loci detected in *T*. *cruzi* I samples and reference clones of other DTUs. The genome-wide distribution of polymorphic segments genotyped using GLST is shown relative to the TcI-Sylvio reference assembly. Blue diamonds represent 303 SNPs detected only in TcI samples and pink diamonds represent 393 SNPs detected only in non-TcI reference clones. Black diamonds represent 84 SNPs detected in both TcI samples and non-TcI reference clones. The close-up illustrates how diamonds representing nearby SNPs (e.g., those occurring on the same GLST target segment) overlap in genome-wide view. Chromosomes 17, 20, 22, 29, 30, 34, 35, 38, 40, 42, 45, 46 and 47 were not targeted by GLST. Chromosome 6 contains one target segment but this segment showed no polymorphism in the sample set.

GLST amplicons contained a total of 780 SNP sites, 387 polymorphic among TcI samples and 393 private to non-TcI reference clones (Fig 2). Seven hundred and seventy-three of these sites were biallelic, and seven contained one additional alternate allele. Median read-depth per individual genotype was 267x, and 90% of genotypes were represented by ≥ 20 reads (S10 Fig). Of 403 loci targeted from the WGS dataset [1], 97% (391) were recovered by GLST and 82 contained polymorphism outside of Ecuador. GLST recovered 80 of 87 SNPs previously identified in TBM_2795_CL2 using WGS. Minimum parasite DNA concentration successfully genotyped from metagenomic DNA was 3.69 pg/μl (sample ECU36–see S11 Fig).

The TBM_2795_CL2 control sample underwent GLST in four replicates. These replicates were identical at all 561 SNP sites for which genotypes were called in all samples of the dataset. Median number of allelic differences (AD = 0, 1 or 2 per site) at non-missing sites between other replicate pairs was 3 (Table 2). Pairwise AD did not correlate to minimum, maximum or difference in mean read-depth between the two replicates (p < 0.80).

**Table 2:** Allelic differences between GLST replicates. Eighteen samples were processed in 2–4 replicates after DNA extraction. A single SNP locus can differ by 0, 1 or 2 between two replicates (i.e., replicates can match at both, one or neither allele). The AD measurement represents the total number of pairwise differences across all loci for which genotypes are called in all individuals (n = 561). The discrepancy between VZ35814 replicates likely represents barcode contamination with VZ16816 (see close similarity in Fig 4).

Replicate comparison	AD
COL319_rep1 vs. COL319_rep2	0
ECU10_rep1 vs. ECU10_rep2	0
TBM_2795_CL2_rep1 vs. TBM_2795_CL2_rep2	0
TBM_2795_CL2_rep1 vs. TBM_2795_CL2_rep3	0
TBM_2795_CL2_rep1 vs. TBM_2795_CL2_rep4	0
TBM_2795_CL2_rep2 vs. TBM_2795_CL2_rep3	0
TBM_2795_CL2_rep2 vs. TBM_2795_CL2_rep4	0
TBM_2795_CL2_rep3 vs. TBM_2795_CL2_rep4	0
VZ13516_rep1 vs. VZ13516_rep2	0
COL154_rep1 vs. COL154_rep2	1
COL466_rep1 vs. COL466_rep2	1
ECU3_rep1 vs. ECU3_rep2	1
COL135_rep1 vs. COL135_rep2	2
COL468_rep1 vs. COL468_rep2	2
ECU4_rep1 vs. ECU4_rep2	2
COL155_rep1 vs. COL155_rep2	3
COL466_rep1 vs. COL466_rep3	3
COL468_rep1 vs. COL468_rep3	3
COL468_rep2 vs. COL468_rep3	3
VZ6616_rep1 vs. VZ6616_rep2	3
COL466_rep2 vs. COL466_rep3	4
VZ1016B_rep1 vs. VZ1016B_rep2	4
CLBRENER_rep1 vs. CLBRENER_rep2	7
COL133_rep1 vs. COL133_rep2	9
ECU9_rep1 vs. ECU9_rep2	10
COL78_rep1 vs. COL78_rep2	12
VZ35814_rep1 vs. VZ35814_rep2	49

Variant calling was highly consistent: prior to variant filtration, only 10 SNP sites were called from run 1 that were not also called from run 2 (these were excluded from analysis–see Methods). Read-mapping coverage was also strongly correlated between sequencing runs (Pearson's r = 0.93, p < 0.001) (S12 Fig), but marker quantity appeared insufficient for chromosomal copy number estimation (S13 Fig).

### Differentiation among *T*. *cruzi* individuals, sampling areas and DTUs

**Fig 3:**
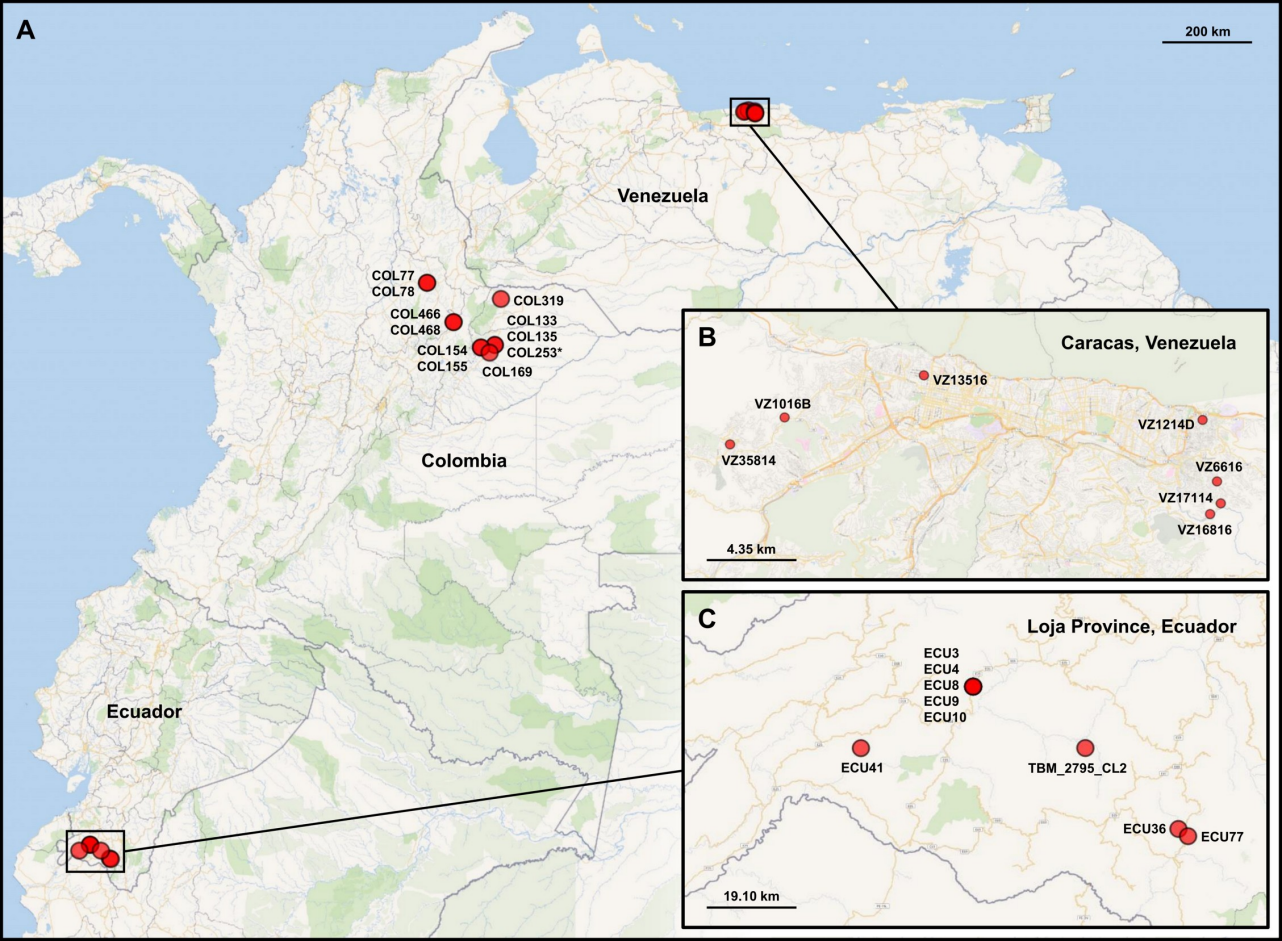
Map of vector sampling sites. A) Sampling in Colombia involved a larger spatial area than that in Venezuela and Ecuador. *T*. *cruzi*-infected intestinal material was collected from *Panstrongylus* and *Rhodnius* vectors in Arauca, Casanare, Santander and Boyacá. COL253 is asterisked because low read-depth led to the exclusion of this sample from all analyses. B) *P*. *geniculatus* material from Venezuela was collected within the Metropolitan District of Caracas. C) *P*. *chinai* and *R*. *ecuadoriensis* material from Ecuador was collected in Loja Province. S1 Table lists coordinates and other sample details.

**Fig 4:**
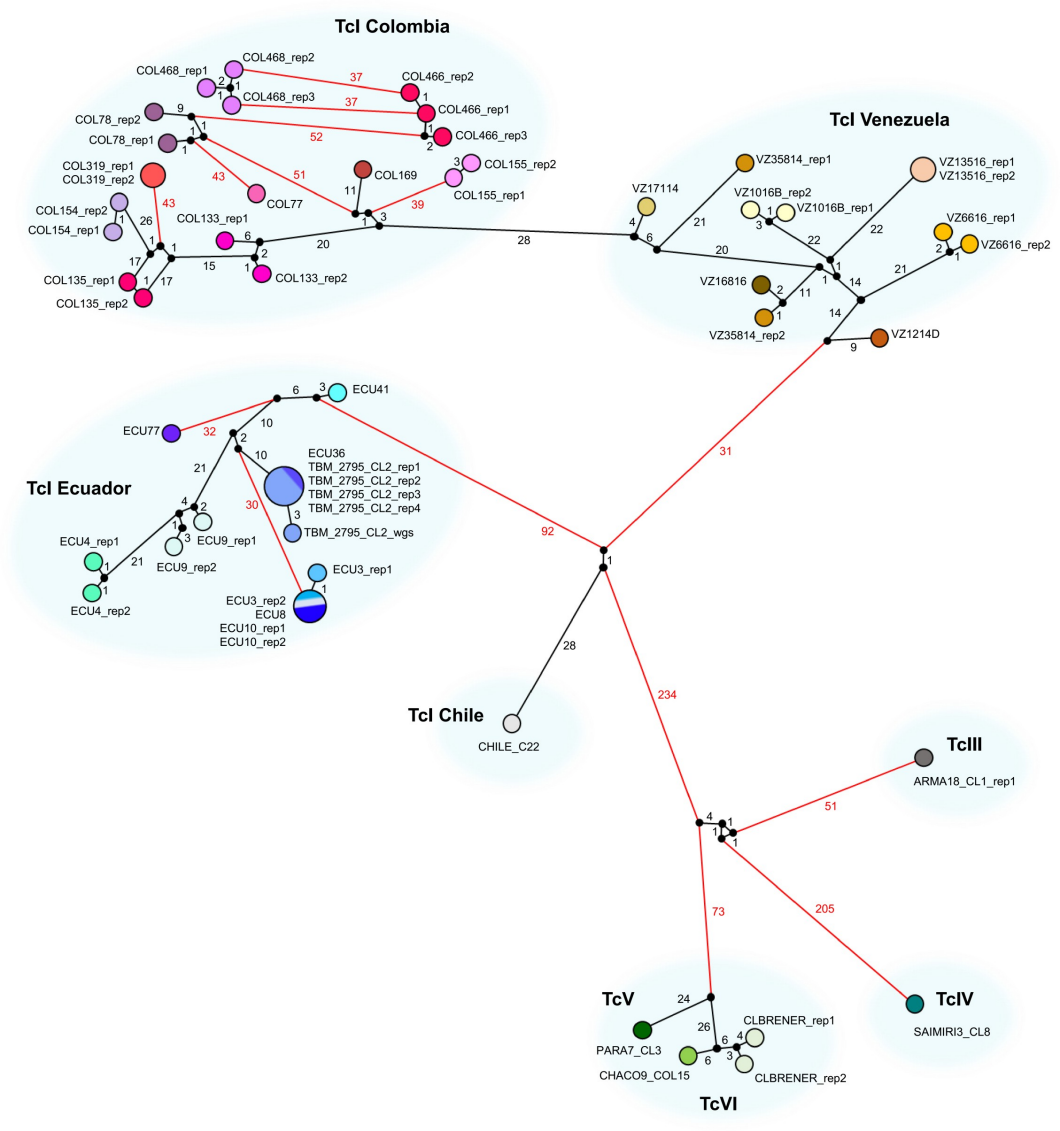
Allelic Differences among *T*. *cruzi* I samples and reference clones of other DTUs as a median-joining network. A single SNP locus can differ by 0, 1 or 2 between two individuals (i.e., the individuals match at both, one or neither allele). The AD measurement indicated on each edge of the network represents the total number of differences across all loci for which genotypes were called in all individuals of the dataset (n = 561). Red edges indicate differences of 30 and above. Technical replicates are represented by circles of the same fill color. Larger circles represent the occurrence of identical GLST genotypes. Edge length is not directly proportional to AD.

Sampling sites in Colombia, Venezuela and Ecuador are plotted in Fig 3, and a median-joining network of allelic differences among GLST genotypes is shown in Fig 4. GLST clearly distinguished TcI individuals at common collection sites in Soata (COL466 vs. COL468, AD = 37), Paz de Ariporo (COL133 vs. COL135, AD = 33), Tamara (COL154 vs. COL155, AD = 107) and Lebrija (COL77 vs. COL78, AD = 43) municipalities of Colombia but not in the community of Bramaderos (ECU3 vs. ECU8 vs. ECU10, AD = 0) in Loja Province, Ecuador. Samples from nearby sites within Caracas, Venezuela were also clearly distinguished by GLST (e.g., VZ16816 vs. VZ17114, AD = 43). Nucleotide diversity (π = mean pairwise AD) was higher in samples from Caracas (π = 29.0) than in those from Loja Province (π = 22.8) but not in those from Colombia (π = 43.2) (Table 3). Hardy-Weinberg ratios, linkage and inbreeding coefficients are also listed in Table 3.

**Table 3:** Basic diversity statistics for *T*. *cruzi* I samples from Colombia (COL), Venezuela (VZ) and Ecuador (ECU).

Group (n)	PS	PS in HWE	F_IS_ (Q1, M, Q3)	r^2^ (Q1, M, Q3)	π	F_ST_ to COL	F_ST_ to VZ	F_ST_ to ECU
COL (11)	175	169	-0.19, 0.13, 0.24	0.03, 0.07, 0.19	43.2	0.000	0.136	0.595
VZ (7)	147	143	-0.35, -0.19, 0.11	0.02, 0.09, 0.27	29.0	0.136	0.000	0.632
ECU (9)	148	142	-0.20, -0.09, 0.18	0.04, 0.17, 0.36	22.8	0.595	0.632	0.000

Abbreviations: n, sample size; PS, polymorphic sites; HWE, Hardy-Weinberg equilibrium; F_IS,_ inbreeding coefficient; r^2^, linkage coefficient; π, nucleotide diversity; Q, quartile; M, median; F_ST,_ between-group fixation index.

**Fig 5:**
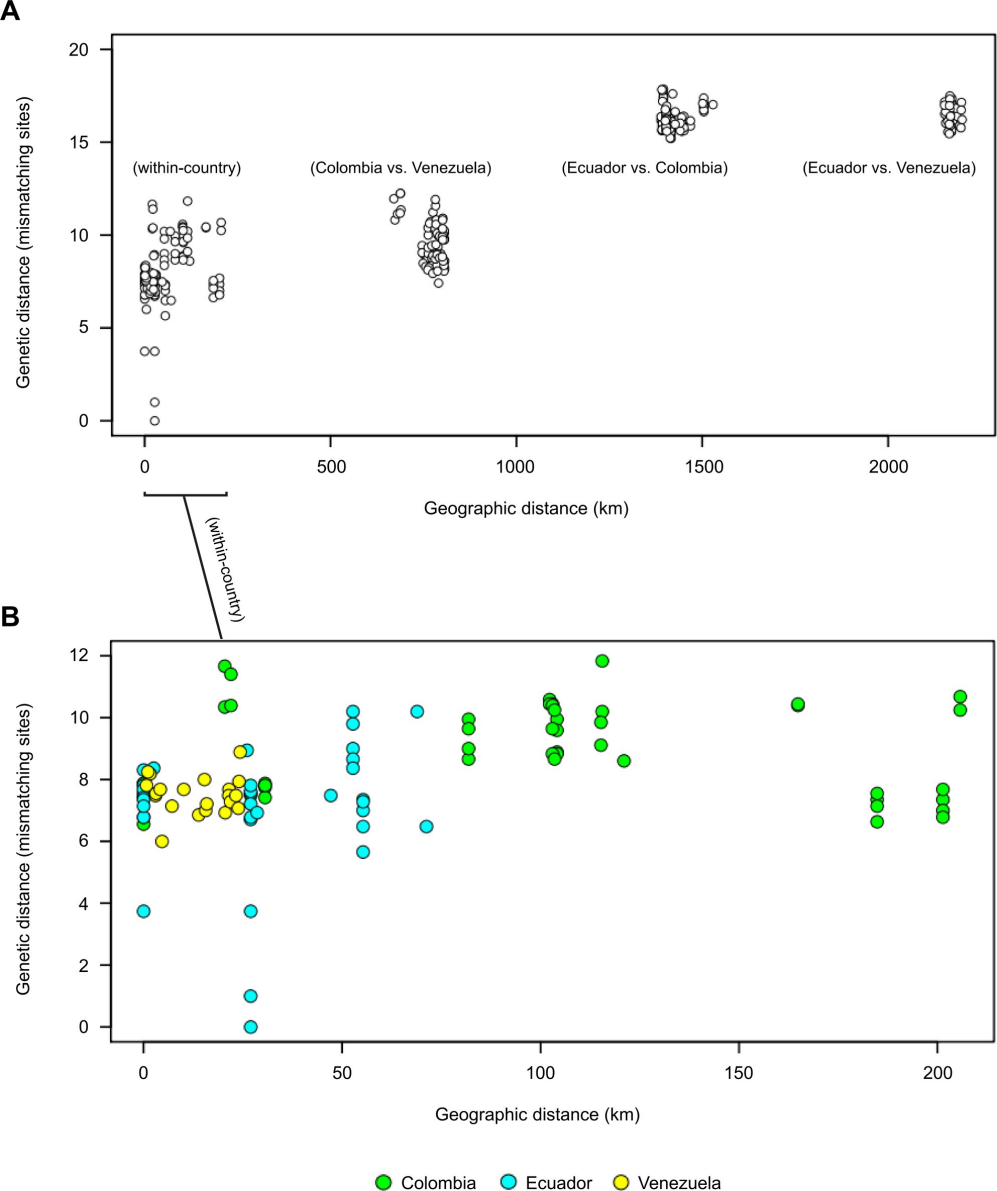
Spatio-genetic correlation among *T*. *cruzi* I samples. A) Each circle represents geographic and genetic distances between two TcI samples. Positive correlation in the multi-country dataset (Mantel’s r = 0.89, p = 0.001) is driven by divergence between samples from Ecuador and Colombia/Venezuela (see two clusters at top right). B) Nevertheless, this relationship remains significant for within-country comparisons at < 250 km (Mantel’s r = 0.30, p = 0.009) and < 150 km (Mantel’s r = 0.48, p = 0.002). Green, cyan and yellow fill colors represent comparisons within Colombia, Ecuador and Venezuela, respectively. Each of the above Mantel tests remains significant when sample pairs with genetic distances < 2 are removed. Only variant sites with ≤ 10% missing genotypes (n = 285) are used in analysis. Only the first replicate is used for samples represented by multiple replicates.

Genetic distances increased with spatial distances among samples (Mantel’s r = 0.89, p = 0.001), but the correlation coefficient was largely driven by high F_ST_ between sample sets from Colombia/Venezuela and Ecuador (Table 3 and Fig 5A): Mantel’s r decreased to 0.30 (p = 0.001) after restricting analysis to sample pairs separated by < 250 km (Fig 5B). Within-country spatio-genetic correlation appeared stronger for samples separated by < 150 km (Mantel’s r = 0.48, p = 0.002) given a lack of correlation observed at higher distance classes within the Colombian dataset (Fig 5B).

**Fig 6:**
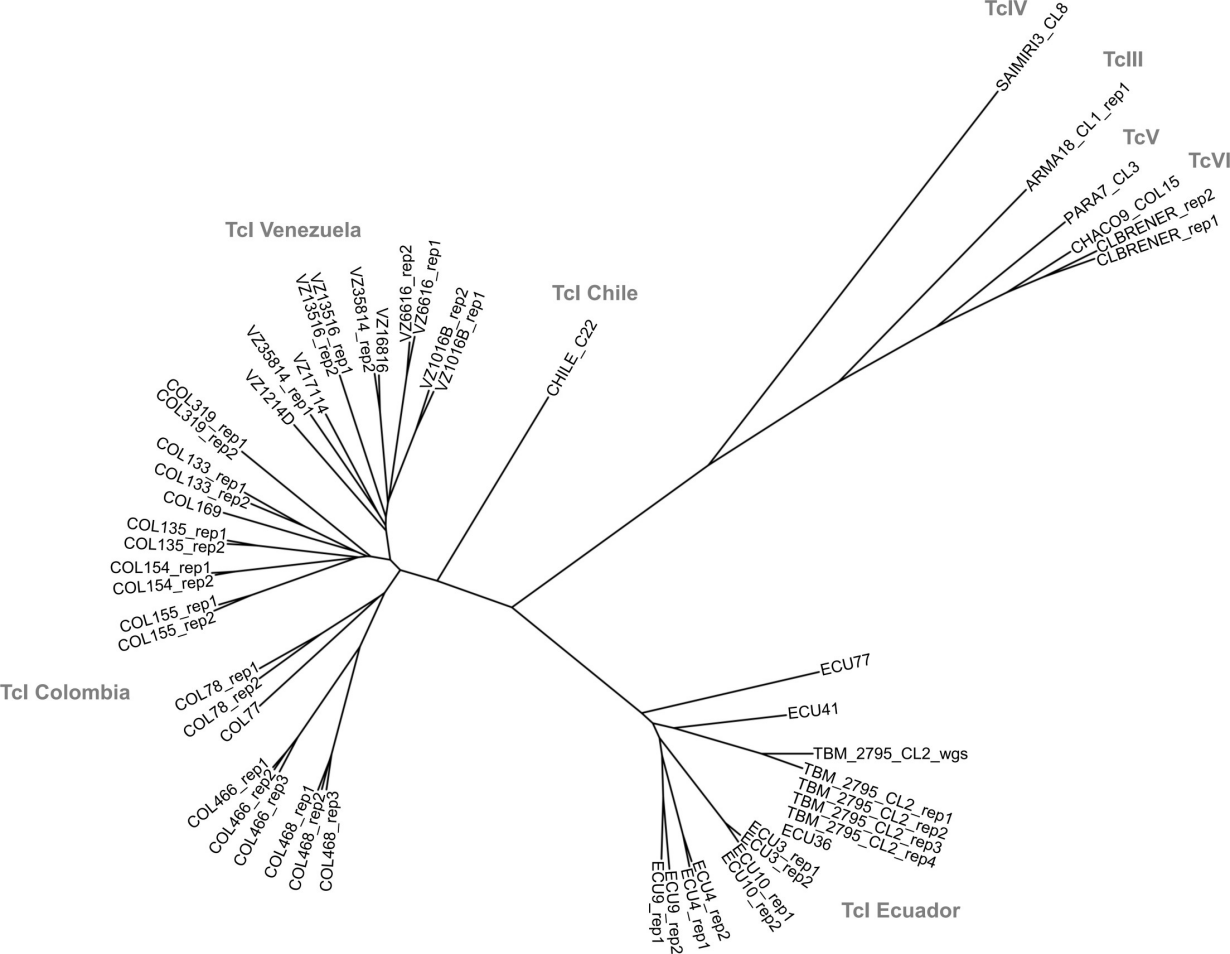
Neighbor-joining relationships among *T*. *cruzi* I samples and reference clones of other sub-lineages. Genetic distances are based on 556 biallelic SNP sites for which genotypes are called in all individuals. Results indicate high repeatability among most technical replicates (see ‘rep1–4’ suffices) and clearly separate TcI, TcIII, TcIV and TcV + TcVI. The tree also contains TBM_2795_CL2_wgs. This control sample was genotyped at the same 556 GLST loci using whole-genome sequencing (Illumina HiSeq) data from Schwabl *et al*. 2019 [1]. See S14 Fig for a tree with additional reference clones (genotypes generated *in silico* by subsetting WGS variant calls to GLST targets).

GLST also clearly separated DTUs TcI, TcIII, TcIV and TcV + TcVI in network (Fig 4) and neighbor-joining tree construction (Fig 6). AD between reference clones of different DTUs ranged from 153 (ARMA18_CL1 (TcIII) vs. PARA7_CL3 (TcV)) to 472 (CHILE_C22 (TcI) vs. SAIMIRI3_CL8 (TcIV)).

### Heterozygosity and allele frequency distributions

**Fig 7:**
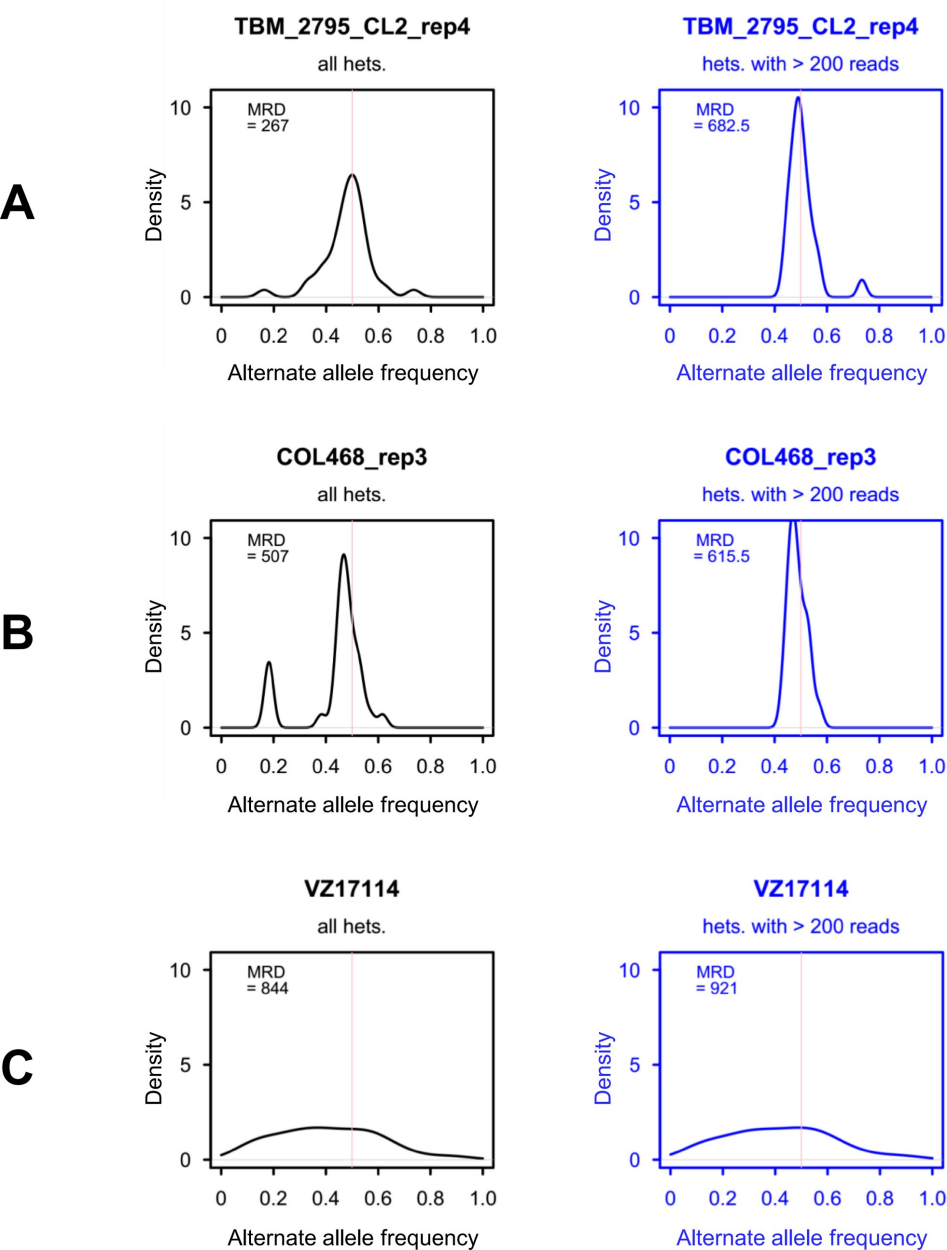
Alternate allele frequency distributions of heterozygous genotypes at biallelic sites. A) Alternate allele frequency (i.e., the number of non-reference reads divided by the total number of reads representing each genotype) had a mode near 50% in most samples, e.g., see TBM_2795_CL2. B) Distinct and/or additional modes frequently diminished when excluding genotypes represented by ≤ 200 reads (black vs. blue plot). C) For approximately one third of samples, distinct allele frequency distributions did not change after setting this exclusion. S15–S17 Figs provide plots for the full sample set. Plots were generated using the ‘density’ function in R. Abbreviations: MRD, median read-depth of heterozygous genotypes; hets., heterozygous genotypes.

Alternate allele frequencies measured in heterozygous genotypes at biallelic sites were distributed with a single strong mode near 50% in most samples (Fig 7A, S15–S17 Figs, S3 Table), suggesting many strains were predominantly diploid and potentially monoclonal. In a limited number of samples, alternate allele frequency distributions (AFDs) showed secondary modes and/or no clear mode near 50% but these irregularities diminished after excluding genotypes represented by ≤ 200 reads (e.g., see COL_468 in Fig 7B). Irregular AFDs observed for replicates of ECU4, COL78, COL133, COL135, COL169 (S15–S17 Figs) and VZ17114 (Fig 7C), however, showed no substantial change after this exclusion and were highly consistent between available replicates. AFDs in these six individuals, all of which had substantial median read-depth (253 ≤ MRD ≤ 924), did not appear symptomatic of frequent copy number variation at heterozygous sites (i.e., no strong peaks at 25%, 33%, 67% or 75% as might occur if many loci existed in three or four copies instead of two). Possibly representing multiclonal infections, this group of samples showed a higher median rate of heterozygosity per polymorphic genotype (HPG, S3 Table) than did the remainder of the dataset (71% vs. 50%) (Wilcoxon test, W = 144, p = 0.002). HPG in replicates of presumably monoclonal TcI clones TBM_2795_CL2 and Chile_C22, by contrast, ranged between 39% and 44% (S3 Table). Excluding highly heterozygous TcV and TcVI clones (S3 Table), median number of heterozygous SNPs (i.e., absolute counts as opposed to HPG) was also higher in these six samples than in the remainder of the dataset (Wilcoxon test, W = 127.5, p = 0.002). Despite these possible signs of multiclonality, however, we found little evidence for within-sample polyallelism across the 26,042 sites targeted by GLST. Between zero and ten sites (0.04%) showed reads representing more than two alleles within any single TcI sample–the maximum observed in VZ1016B_rep2 (S3 Table). Within-sample polyallelism in non-TcI clones ranged from one (in ARMA18_CL1_rep1) to 28 (in PARA7_CL3) (S3 Table).

## Discussion

### Principle results

The GLST primer panel design and amplicon sequencing workflow outlined in this study aimed to profile *T*. *cruzi* genotypes at high resolution directly from infected triatomine intestinal content by simultaneous amplification of 203 genetic target regions that display sequence polymorphism in publicly available WGS reads. Mapped GLST amplicon sequences generated from *T*. *cruzi* reference clones and from metagenomic intestinal DNA extracts containing a minimum of 3.69 pg/μl *T*. *cruzi* DNA achieved high target specificity (< 1% off-target mapping) and yield (391 of 403 target SNP sites mapped). Mapping depth variation across target loci was highly repeatable between sequencing runs. Three hundred and eighty-seven SNP sites were identified among *T*. *cruzi* I samples and 393 SNP sites were identified in non-TcI reference clones. These markers showed low levels of linkage disequilibrium at fine spatial scales (e.g., within Caracas) and clearly separated *T*. *cruzi* individuals within and across DTUs, for the most part also individuals collected at the same or closely separated localities in Colombia, Venezuela and Ecuador. An increase in pairwise genetic differentiation was observed with increasing geographic distance in analyses within and beyond 150 km. Finally, we observed similar abundances of reads representing alternate and reference alleles at heterozygous sites in monoclonal TcI reference clones. Distinct alternate allele frequency distributions in a subset of field samples suggested the detection of multiclonal infections using GLST.

### Cost-effective spatio-genetic analysis

GLST achieved an important resolution benchmark in recovering isolation-by-distance (IBD) [70] at less than 150 km. These correlations indicate the potential of GLST in spatially explicit epidemiological studies which, for example, aim to identify environmental variables or landscape features that modify IBD [28]. High spatial sampling effort is typically required by such studies and often limits budget for genotyping tools. GLST appears promising in this context as it bypasses pathogen culture and library preparation (< 4 USD per sample (see cost summary in S4 Table)) can be completed comfortably in two days. The first-round PCR reaction requires very low primer concentrations (0.125 μM) such that a single GLST panel purchase (0.01 μmol production scale) enables > 100,000 reactions and can be shared by several research groups. Sequencing represents a substantial cost but is highly efficient due to short fragment sizes and few off-target reads. High library complexity also promotes the use of GLST libraries as an alternative to PhiX, i.e., as a spike-in to enhance complexity and thus read quality in a different sequencing run. Our study easily decontaminated reads from a spiked amplicon pool sharing barcodes with GLST (run 1). Alternatively, i.e, when GLST is sequenced alone (run 2), one Illumina MiSeq run can generate > 70x median genotype read-depth for 100 samples using Reagent Micro Kit v2 (starting at ca. 1,500 USD, depending on provider–see S4 Table). Read-depth can likely be elevated substantially by improving normalization and clean-up steps.

### GLST in relation to multi-locus microsatellite typing

We consider multi-locus microsatellite typing (MLMT) as the primary alternative for high-resolution *T*. *cruzi* genotyping directly from metagenomic DNA. MLMT has revolutionized theory on *T*. *cruzi* ecology and microevolution, for example, on the role of disparate transmission cycles [71,72], ecological host-fitting [73] and ‘cryptic sexuality’ [74] in shaping population genetic structure in TcI. In some cases [75,76] (but others not [72,73,77]), the hypervariable, polyallelic nature of microsatellites allows every sample in a dataset to be distinguished with a different multi-locus genotype (MLG). This depends on panel size and spatial scale but also on local reproductive modes–for example, sampling from clonal sylvatic vs. non-clonal domestic transmission cycles has correlated with the presence or absence of repeated MLGs [72]. In this study, we found two identical GLST genotypes shared among five samples from southern Ecuador. All other samples appeared unique, including those from Venezuela, where triatomine collection occurred at seven domestic localities within the city of Caracas. The small subset of repeated genotypes found in this study may reflect patchy, transmission cycle-dependent clonal/sexual population structure in southern Ecuador (see Schwabl *et al*. 2019 [1] and Ocaña-Mayorga *et al*. 2010 [72]) but may also represent a weakness in GLST compared to MLMT in tracking individual parasite strains. The use of large MLMT panels, however, is significantly more resource-intensive because each microsatellite marker requires a separate PCR reaction and capillary electrophoresis cannot be highly multiplexed. MLMT data are poorly archivable across studies and may also be less suitable for inter-lineage phylogenetic analyses due to unclear mutational models and artefactual similarity from saturation effects [78]. Although our GLST panel was designed for TcI, its focus on orthologous sequence regions enabled efficient co-amplification of non-TcI DNA. GLST clearly separated TcI samples from all non-TcI reference clones, with highest divergence observed in SAIMIRI3_CL8. Interestingly, large MLMT panels have shown comparatively little differentiation between this sample and TcI, also more generally suggesting that TcIV and TcI represent monophyletic sister clades [78]. By detecting substantially higher heterozygosity in TcV and TcVI clones, GLST also showed its potential to distinguish hybrid genotypes in a sample set. These DTUs are known to originate from ancient hybridization events between progenitors of TcII and TcIII [79].

### Adjustment and transferability

Considering the great variety of sample types to which studies have successfully applied PCR [80–84], we expect that GLST can be applied to metagenomic DNA from many host/vector tissue types, not only from triatomine intestine as shown here. Further tests are required to determine whether low *T*. *cruzi* DNA concentrations in chronic infections or sparsely infected organs (e.g., liver and heart [85]) are also amenable to GLST. We predominantly analyzed *T*. *cruzi* DNA concentrations of at least ten picograms (this equates to approximately 80 parasites in the case of TcI [86]) per microliter metagenomic DNA without heavily investigating options to enhance sensitivity or sensitivity measurement, for example, by additional removal of PCR inhibitors, improved primer purification (e.g., HPLC vs. salt-free), post-PCR probe-hybridization [87] or barcoding/sequencing of samples with unclear first-round PCR amplicon bands. Even relatively aggressive processing methods may be tolerable given that DNA fragmentation is unlikely to compromise the 120–160 nt size range targeted by GLST. Increasing sensitivity by increasing PCR amplification cycles, however, is less advised. PCR error appeared relevant with as little as 30x (+ 7x barcoding) amplification in this study as we observed noise among replicates despite high read-depth and SNP-call overlap between sequencing runs. Rates of error were, however, well within margins expected for methods involving PCR [88]. We also note that the exceptional discrepancy between VZ35814 replicates unlikely represents systematic error but barcode contamination with VZ16816. Such error is perhaps less likely if primers are kept in separate vials instead of in the plate format which we have used here.

Wet lab aside, the main objective of this study was to provide a transparent bioinformatic workflow for highly multiplexable primer panel design using freely available softwares and publicly archived WGS reads (https://github.com/fishntryps/glst). Importantly, we show that knowledge of polymorphic genetic regions in parasite genomes from one small study area (Loja Province, Ecuador) can suffice to guide variant discovery at distant, unassociated sampling sites. Our demonstration using *T*. *cruzi* should be easily transferable to any other pathogenic species with a published reference genome. Target selection can also be tailored to a variety of objectives. For example, while landscape genetic studies on dispersal often focus on neutral or non-coding sequence variation [89], experimental (e.g., drug testing) studies may seek to detect single-nucleotide changes in coding regions, perhaps in genes belonging to specific ontology groups or associated with results of high-throughput proteomic screens [90]. The candidate SNP pool can easily be filtered for such criteria during GLST panel design, e.g., using SnpEff [91] or BEDTools [92] and data mining strategies at EuPathDB [93]. Candidate SNP filtering by minor allele frequency (MAF) may also be useful when the target population is closely related to that of the WGS dataset guiding panel design. Placing a minimum threshold on MAF (using VCFtools [61], etc.), for example, may improve analyses of population structure and genealogy whereas a focus on low-frequency variants may help in tracking individuals or recent gene flow at the landscape scale [94]. It may also be possible to refine panel design towards markers that meet model assumptions in later analysis. Hardy-Weinberg Equilibrium (HWE), for example, is a common requirement in demographic modelling [95–97], Bayesian clustering [98], admixture/migration [99,100] and hybridization tests [101]. Deviation from HWE may occur more frequently in specific genetic regions (e.g., near centromeres [102]), and SNPs in these regions could be excluded from the target pool. Numerous other filtering options–e.g., based on allele count (to enhance resolution per SNP), distance to insertion-deletions (to improve target alignment) or percent missing information (to avoid poorly mapping regions)–are easily implemented with common analysis tools [103].

GLST is also highly scalable because increasing panel size does not lead to more laboratory effort or processing time. Sequencing depth requirements and thermodynamic compatibilities among primers are more relevant in limiting panel size. However, it is also possible to divide large GLST panels into two or more PCR multiplexes based on ΔG-based partitioning in MultiPLX [54]. Unintended primer affinities (i.e., polymer formations) can also be removed by gel excision, e.g., as we have done using the PureLink Quick Gel Extraction Kit.

### Prospects

This study sought to provide a framework for various epidemiological research but remains tentative with its own inferences on *T*. *cruzi* ecology because only few samples (low-quality remainders from different projects) were analyzed from each study area. Samples were also aggregated either to domestic or to sylvatic ecotopes. More extensive, purposeful sampling could have, for example, helped explore whether COL468’s position deep within the Cordillera Oriental contributes to its divergence to samples such as COL135 or COL319, these perhaps more closely related due to lower ‘cost-distances’ [104] of dispersal along the basin range. On the other hand, could relatively low divergence between geographically distant Colombian samples (e.g., differentiation between COL135 and COL319 (separated by ca. 100 km) appears similar to that between VZ1214D and VZ13516 within Caracas (AD = 60 and 61, respectively)) reflect long-range, human-associated dispersal events? Or could restraints to polymorphism within core sequence regions be limiting divergence within TcI? Achieving better resolution of genetic differentiation and dispersal in wild vs. domestic *T*. *cruzi* populations using neutral genetic markers is an exciting new direction for GLST. Fuelled with high GLST sample sizes, landscape genetic simulators such as CDMetaPOP [97] could be especially powerful to this end. It would also be interesting, for example, to extend this study’s sampling to cover gradients along the perimeter of Caracas and adjacent El Ávila National Park. Sylvatic *P*. *geniculatus* vector populations appear to be rapidly adapting to habitats within Caracas [45,105] but parallel changes in the distribution of *T*. *cruzi* genetic diversity have yet to be tracked. The low cost of GLST also makes it more feasible for studies to simultaneously assess genetic polymorphism in each vector individual from which parasite markers were amplified. Such coupled genotyping would enhance resolution of parasite-vector genetic co-structure and thus, for example, help quantify rates of parasite transmission from domiciliating vectors or determine whether parasite gene flow proxies for (or improves understanding of) dispersal patterns in more slowly evolving vectors or hosts. It would also be interesting to test whether deep-sequenced GLST libraries could be used to reconstruct distinct MLGs from multiclonal *T*. *cruzi* infections without the use of cloning tools. Multiclonality has important implications for public health [106,107] but its potential prevalence in *T*. *cruzi* vectors and hosts [108–110] is difficult to describe from cultured cells [108,111]. In this study, alternate allele frequency modes (at heterozygous sites) were either consistently similar or consistently dissimilar to 50%, suggesting that read-depth ratios generated by GLST are informative of initial allelic ratios and can distinguish monoclonal from multiclonal infections. Whether sequencing coverage and other settings can be optimized to clearly parse (low-frequency) MLGs, however, remains to be established (e.g., using experimental co-infections).

The potential to assess karyotypic variability on the basis of GLST read-depth statistics likewise requires further investigation. A reduced number of PCR cycles and a significantly larger number of markers may be necessary based on relationships between copy number measurement accuracy and genome coverage recently described in work on *Leishmania* parasites [20].

Future applications of GLST will help refine the method as well as clarify its limitations and its areas of greatest impact. We see a particular benefit to population and landscape genetic studies, in which prudent spatial and genetic sampling design is often key to meaningful inference. The low cost and high flexibility of our pipeline can help researchers achieve these requirements without extensive technical know-how and within reasonable costs and time.

## Supporting information

S1 Fig Phylogenetic resolution at GLST loci *in silico*. The green tree shows neighbor-joining (NJ) relationships calculated from 106,007 SNP sites identified from whole-genome sequencing (WGS) of 45 TcI clones in southern Ecuador [1]. Sites missing genotypes in ≥ 10% individuals are excluded. Less than 45 km separate the most distant sampling sites within the study region. Several pairs of clones also represent the same host/vector individual (see first seven characters of IDs). NJ was repeated after abridging the WGS dataset to contain only SNPs within the 203 sequence targets proposed by GLST (also excluding sites missing ≥ 10% genotypes). This resultant tree (blue, at right) uses 391 SNP sites and recreates clusters A–K observed in WGS.
(TIF)

S2 Fig Individual primer pair validation. Primer pairs were first applied individually to pure TcI epimastigote DNA to confirm product amplification within the expected size range (164–204 bp). The figure shows the electrophoresed products of 17 different primer pairs in 0.8% agarose gel as well as DNA ladder (L) and no-template control (NTC). All other primer pairs achieved similar results using an initial incubation step at 98°C (2 min); 30 amplification cycles at 98°C (10 s), 60°C (30 s) and 72°C (45 s); and a final extension step at 72°C (2 min).
(TIF)

S3 Fig Preliminary GLST (multiplex) trials on *T*. *cruzi* I mock infections. We created mock infections by mixing 10^4^, 10^5^ and 10^6^ RNAlater-preserved TcI-Sylvio epimastigote (epi) cells with uninfected *R*. *prolixus* vector gut (UVG). DNA extracted from these mock infections was subjected to the multiplexed, 203-target GLST reaction (using the same cycling conditions as for single-target reactions–see Methods or S2 Fig legend) and products were electrophoresed in 0.8% agarose gel. Fainter banding of GLST products from lower concentration mock infections encouraged follow-up on sensitivity thresholds using additional dilution curves and qPCR. Next to DNA ladder (L) and no-template control (NTC), the gel also contains TcZ primer product from pure TcI epimastigote DNA. TcZ primers provide a highly sensitive positive control (PC) as they target 195 bp satellite DNA repeats that make up ca. 5% of the *T*. *cruzi* genome.
(TIF)

S4 Fig *T*. *cruzi* I DNA dilutions and GLST product visibility in 0.8% agarose gel. The left side shows electrophoresed GLST amplicons generated from 3 μl pure TcI epimastigote (epi) DNA with concentrations between 1.35 ng/μl and 2.50 pg/μl (see cycling conditions in Methods or S2 Fig legend). Lanes on the right contain amplicons from seven random metagenomic samples that tested positive for *T*. *cruzi* satellite DNA. DNA ladders (L) and no-template control (NTC) are indicated left and right. Poor amplicon visibility occurs at ≤ 30 pg epimastigote DNA input (3 μl). Gut DNA amplicon visibility is also limited but whether this relates to low *T*. *cruzi* content or amplification interference is unclear without qPCR.
(TIF)

S5 Fig First-round (unbarcoded) PCR product size composition measurement using microfluidic electrophoresis. The figure plots fragment sizes (calculated based on migration times relative to those of standards) and fluorescence intensity (FU) of first-round PCR products (see cycling conditions in Methods or S2 Fig legend) measured with the Agilent Bioanalyzer 2100 System. The first peak represents primer polymerization that is removed in subsequent gel excision/re-solubilization steps. The second peak matches expectations for the multi-target GLST product (164–204 bp).
(TIF)

S6 Fig Large polymer formation from excessive amplicon barcoding. The second (barcoding) PCR reaction uses an initial incubation step at 98°C (2 min); 7 amplification cycles at 98°C (30 s), 60°C (30 s) and 72°C (1 min); and a final extension step at 72°C (3 min). Seven amplification cycles were chosen because unwanted polymers formed at 13 and 18x. The center lanes in the 0.8% agarose gel at left (red border) show electrophoresed GLST products from reference clones after eighteen cycles of barcoding PCR. Large, non-target banding occurs at ≥ 300 bp. Unbarcoded products from TcI epimastigote (epi) DNA are also shown at left. No template controls from barcoding (NTC) and first-round + barcoding PCR (NTC*) occur next to the DNA ladder (L) on the right side of the gel. The smaller image (green border) to the right shows how unwanted banding becomes less pronounced at 13x and largely disappears at 7x. This 0.8% agarose gel also contains NTC* samples, i.e., negative controls carried through both first and second-round PCR.
(TIF)

S7 Fig Barcoded GLST products ready for final pooling and purification. The 0.8% agarose gel shows a subset of fifteen GLST products from the second-round (barcoding) PCR reaction (see cycling conditions in Methods or S6 Fig legend) prior to equimolar pooling and final gel excision/re-solubilization steps. Products from ECU6 and ECU2 occur in this gel but were not included in the final pool. The gel also contains DNA ladder (L) and no-template controls from barcoding (NTC) and first-round + barcoding PCR (NTC*).
(TIF)

S8 Fig Final (barcoded) GLST pool size composition measurement using microfluidic electrophoresis. The figure plots fragment sizes (calculated based on migration times relative to those of standards) and fluorescence intensity (FU) of the final GLST pool measured with the Agilent Bioanalyzer 2100 System. The large peak matches expectations for the multi-target GLST product pool (224–264 bp). Left and right peaks labelled in green and purple represent standards of known size. A small non-target peak remaining near 151 bp encourages improvement of prior size selection steps.
(TIF)

S9 Fig Quality scores at previously identified vs. unidentified variant sites. The GLST primer panel was designed based on single-nucleotide polymorphisms (SNPs) in Ecuadorian TcI clones. It was applied, however, to samples from distant geographic locations as well as to non-TcI clones. Additional, previously unidentified SNP sites (PU) were thus expected to be found but we needed to distinguish true PU from PCR and sequencing error. We reasoned that quality statistics (e.g., mapping quality, strand bias, minor allele frequency, etc.–see Methods) at previously identified SNP sites (PI) could help calibrate quality filters applied to the wider dataset. This strategy finds support in the above density plot of QUAL scores computed by GATK [48]. The plot suggests that, prior to variant filtration, lower QUAL scores occur more often at PU (red) than at PI (black). We thus imposed the most stringent filtering criteria possible without losing PI.
(TIF)

S10 Fig Histogram of read-depths per genotype. Median read-depth is 267x including zero-depth genotypes (6% of total) and 309x excluding zero-depth genotypes.
(TIF)

S11 Fig GLST sample selection and sensitivity estimation via qPCR. We used *T*. *cruzi* satellite DNA qPCR to identify vector gut samples with *T*. *cruzi* DNA quantities within ranges successfully visualized in GLST reactions using epimastigote DNA (S4 Fig). The qPCR reaction used an initial incubation step at 95°C (10 min) and 40 amplification cycles at 95°C (15 s), 55°C (15 s) and 72°C (15 s). The plot shows baseline-corrected fluorescence (dR) for seven sample duplicates. Following the regression equation from the standard curve (see inset), the three samples with highest cycle thresholds (Ct values) in this example represent gut extracts with 0.05 to 0.14 ng/μl *T*. *cruzi* DNA. Such samples with *T*. *cruzi* DNA concentrations above 0.01 ng/μl were prioritized for GLST and none failed in library construction. ECU36, with a mean Ct value of 18.68 in the plot, was also successfully sequenced. A Ct value of 18.68 represents 3.69 pg/μl *T*. *cruzi* DNA. Not all samples with concentrations at single-digit picogram levels (per μl) were successful and we did not troubleshoot those with substantially lower concentrations based on qPCR.
(TIF)

S12 Fig Similar read-depth distribution between separate sequencing runs. We sequenced the same GLST pool in two separate Illumina MiSeq runs. Run 1 involved GLST as a spike to a collaborator’s 16S amplicon library, whereby GLST reads were subsequently decontaminated from (barcode-sharing) 16S reads by alignment to the TcI-Sylvio reference genome. GLST libraries were sequenced alone in run 2. Read-depths at each GLST base position (purple points) are highly correlated between the two runs (Pearson's r = 0.93, p < 0.001). Run 1 had higher sequencing output than run 2. Values are square-root transformed and represent the control sample TBM_2975_CL2_rep1.
(TIF)

S13 Fig Target coverage in control replicates confirms expectations that the GLST panel applied in this study is unreliable for chromosome copy number estimation. We adapted methods from Schwabl *et al*. 2019 [1] to derive somy estimates for each base position within GLST amplicons. Briefly, we calculated median-read-depth of all target bases for each chromosome. We let the median of these chromosomal medians (the ‘inter-chromosomal median’) represent expectations for the disomic state, estimating copy number per base position by dividing each position’s read-depth by the inter-chromosomal median and multiplying by two. Boxplots show median and interquartile ranges of these site-wise somy estimates for each chromosome in TBM_2975_CL2 control replicates. TBM_2795_CL2 did not show chromosomal amplifications in whole-genome analysis [1]. Not unexpectedly for a PCR-based method, somy values estimated from GLST read-depths differ substantially among replicates and are unrealistically high/low on many chromosomes. Estimates on chromosomes with few GLST targets appear especially unreliable–e.g., see chromosomes 8, 28, 33, 39 and 43. These chromosomes contain ≤ 2 GLST targets each. Horizontal cyan lines mark y = 1.5 and y = 2.5.
(TIF)

S14 Fig Neighbor-joining relationships among *T*. *cruzi* I samples and additional reference clones. The tree uses seven reference clones (red font with WGS run accessions) in addition to those from Fig 6. We genotyped these clones *in silico* by subsetting genome-wide variant calls to retain only those occurring within GLST target regions (excluding primer binding sites). Of these, 585 were biallelic and had genotypes called in all individuals. These 585 sites were used for the Euclidean distance matrix of alternate allele counts underlying the tree. The two clones from Colombia and Venezuela represent members of the widespread human-associated ‘TcI_DOM_’ genotype [71]. The close clustering of these two clones is consistent with previous WGS analyses showing low diversity among geographically disparate TcI_DOM_ isolates [52]. No other TcI samples of the dataset appear to belong to the TcI_DOM_ genotype. The addition of TcII (S11 and Y strain cl. 4) [14], TcIII (strain 231) [112], TcV (92–80 cl. 2) and TcVI (Tulahuen cl. 2) (Washington University School of Medicine) demonstrates limited GLST target differentiation between TcV and TcVI relative to that within TcI and among other DTUs.
(TIF)

S15 Fig Alternate allele frequency distributions of heterozygous genotypes at biallelic sites. Alternate allele frequency (i.e., the number of non-reference reads divided by the total number of reads representing each genotype) had a mode near 50% in most samples. Distinct and/or additional modes frequently diminished when excluding genotypes represented by ≤ 200 reads (black vs. blue plots). For approximately one third of samples, distinct allele frequency distributions did not change after setting this exclusion. Alternate allele frequency bins are shown on the x-axis and densities are plotted on y. Abbreviations: MRD, median read-depth of heterozygous genotypes; hets., heterozygous genotypes.
(TIF)

S16 Fig Alternate allele frequency distributions of heterozygous genotypes at biallelic sites. Alternate allele frequency (i.e., the number of non-reference reads divided by the total number of reads representing each genotype) had a mode near 50% in most samples. Distinct and/or additional modes frequently diminished when excluding genotypes represented by ≤ 200 reads (black vs. blue plots). For approximately one third of samples, distinct allele frequency distributions did not change after setting this exclusion. Alternate allele frequency bins are shown on the x-axis and densities are plotted on y. Abbreviations: MRD, median read-depth of heterozygous genotypes; hets., heterozygous genotypes.
(TIF)

S17 Fig Alternate allele frequency distributions of heterozygous genotypes at biallelic sites. Alternate allele frequency (i.e., the number of non-reference reads divided by the total number of reads representing each genotype) had a mode near 50% in most samples. Distinct and/or additional modes frequently diminished when excluding genotypes represented by ≤ 200 reads (black vs. blue plots). For approximately one third of samples, distinct allele frequency distributions did not change after setting this exclusion. Alternate allele frequency bins are shown on the x-axis and densities are plotted on y. Abbreviations: MRD, median read-depth of heterozygous genotypes; hets., heterozygous genotypes.
(TIF)

S1 Table Details on *T*. *cruzi*-infected metagenomic triatomine gut samples from Colombia (COL), Venezuela (VZ) and Ecuador (ECU). (PDF)

S2 Table GLST primer sequences. The 3’ end of each first-round PCR primer is target-specific. The 5’ end of each forward primer contains CS1. The 5’ end of each reverse primer contains CS2. These sequencing primer binding sites are shown in pink. In subsequent barcoding PCR, the reverse primer consists of 5’-CAAGCAGAAGACGGCATACGAGAT*X*TACGGTAGCAGAGACTTGGTCT-3’, where *X* is a unique 10 nt barcode used to label each sample’s sequence reads. The reverse barcoding primer also contains CS2. The forward barcoding primer (5'-AATGATACGGCGACCACCGAGATCTACACTGACGACATGGTTCTA-3') contains CS1 and is the same for all samples.
(PDF)

S3 Table Heterozygosity and allele frequency metrics. *T*. *cruzi* samples/clones are listed in ascending order of total number of heterozygous genotypes (i.e., heterozygosity count in column 2). High heterozygosity counts in PARA7_CL3, CLBRENER and CHACO9_COL15 is consistent with TcV and TcVI originating via hybridization between progenitors of TcII and TcIII [79]. In fact, all 194–210 heterozygous sites found in these three clones match sites at which TcII (variants called from publicly available WGS reads (run accession SRR6357355) [14]) differs from ARMA18_CL1 (TcIII). Heterozygosity per polymorphic genotype refers to the number of heterozygous genotypes divided by the total number of polymorphic genotypes per sample/clone. The fifth column indicates the proportion of all GLST sites (26,042 bp) at which reads representing > 2 alleles were detected with GATK [48] ‘HaplotypeCaller’ algorithm set to ‘-ploidy 4’. This setting allows for tri- and tetra-allelic genotype calls. None occurred.
(PDF)

S4 Table Summary of GLST library preparation and sequencing costs. Green dots indicate items/costs related to first-round PCR and clean-up. Blue dots indicate items/costs related to barcoding PCR and clean-up. The cost summary does not consider qPCR materials because we applied qPCR only for purposes of method development.
(PDF)

## References

[1] Schwabl P, Imamura H, Van den Broeck F, Costales JA, Maiguashca-Sánchez J, Miles MA, et al. Meiotic sex in Chagas disease parasite *Trypanosoma cruzi*. Nat Commun. 2019;10(1):3972 10.1038/s41467-019-11771-z 31481692 PMC6722143

[2] Guerra-Assunção JA, Crampin AC, Houben RMGJ, Mzembe T, Mallard K, Coll F, et al Large-scale whole genome sequencing of *M*. *tuberculosis* provides insights into transmission in a high prevalence area. eLife. 2015;4:e05166 10.7554/eLife.05166 PMC4384740 25732036

[3] Hall MD, Holden MT, Srisomang P, Mahavanakul W, Wuthiekanun V, Limmathurotsakul D, et al Improved characterisation of MRSA transmission using within-host bacterial sequence diversity. eLife. 2019;8:e46402 10.7554/eLife.46402 31591959 PMC6954020

[4] Grigg ME, Bonnefoy S, Hehl AB, Suzuki Y, Boothroyd JC. Success and virulence in *Toxoplasma* as the result of sexual recombination between two distinct ancestries. Science. 2001;294(5540):161–5. 10.1126/science.1061888 11588262

[5] Wu Z, Periaswamy B, Sahin O, Yaeger M, Plummer P, Zhai W, et al Point mutations in the major outer membrane protein drive hypervirulence of a rapidly expanding clone of *Campylobacter jejuni*. Proc Natl Acad Sci U S A. 2016;113(38):10690–5. 10.1073/pnas.1605869113 27601641 PMC5035848

[6] Miotto O, Amato R, Ashley EA, MacInnis B, Almagro-Garcia J, Amaratunga C, et al Genetic architecture of artemisinin-resistant *Plasmodium falciparum*. Nat Genet. 2015;47(3):226–34. 10.1038/ng.3189 25599401 PMC4545236

[7] Auburn S, Benavente ED, Miotto O, Pearson RD, Amato R, Grigg MJ, et al Genomic analysis of a pre-elimination Malaysian *Plasmodium vivax* population reveals selective pressures and changing transmission dynamics. Nat Commun. 2018;9:2585 10.1038/s41467-018-04965-4 29968722 PMC6030216

[8] Teixeira DG, Monteiro GRG, Martins DRA, Fernandes MZ, Macedo-Silva V, Ansaldi M, et al Comparative analyses of whole genome sequences of *Leishmania infantum* isolates from humans and dogs in northeastern Brazil. Int J Parasitol. 2017;47(10–11):655–65. 10.1016/j.ijpara.2017.04.004 28606698 PMC5641220

[9] Devera R, Fernandes O, Coura JR. Should *Trypanosoma cruzi* be called “*cruzi*” complex? a review of the parasite diversity and the potential of selecting population after in vitro culturing and mice infection. Mem Inst Oswaldo Cruz. 2003;98(1):1–12. 10.1590/s0074-02762003000100001 12700855

[10] Alves AM, De Almeida DF, von Krüger WM. Changes in *Trypanosoma cruzi* kinetoplast DNA minicircles induced by environmental conditions and subcloning. J Eukaryot Microbiol. 1994;41(4):415–9. 10.1111/j.1550-7408.1994.tb06099.x 8087110

[11] Dvorak J, Hartman D, Miles M. *Trypanosoma cruzi*: Correlation of growth kinetics to zymodeme type in clones derived from various sources. J Eukaryot Microbiol. 2007;27:472–4.

[12] Deane MP, Jansen AM, Mangia RHR, Gonçalves AM, Morel CM. Are our laboratory “strains” representative samples of *Trypanosoma cruzi* populations that circulate in nature? Mem Inst Oswaldo Cruz. 1984;79(1):19–24.

[13] Lima FM, Souza RT, Santori FR, Santos MF, Cortez DR, Barros RM, et al Interclonal variations in the molecular karyotype of *Trypanosoma cruzi*: chromosome rearrangements in a single cell-derived clone of the G strain. PLoS One. 2013;8(5):e63738 10.1371/journal.pone.0063738 23667668 PMC3646811

[14] Reis-Cunha JL, Baptista RP, Rodrigues-Luiz GF, Coqueiro-dos-Santos A, Valdivia HO, de Almeida LV, et al Whole genome sequencing of *Trypanosoma cruzi* field isolates reveals extensive genomic variability and complex aneuploidy patterns within TcII DTU. BMC Genomics. 2018;19(1):816 10.1186/s12864-018-5198-4 30424726 PMC6234542

[15] Messenger LA, Miles MA, Bern C. Between a bug and a hard place: *Trypanosoma cruzi* genetic diversity and the clinical outcomes of Chagas disease. Expert Rev Anti Infect Ther. 2015;13(8):995–1029. 10.1586/14787210.2015.1056158 26162928 PMC4784490

[16] Cuypers B, Domagalska MA, Meysman P, Muylder G de, Vanaerschot M, Imamura H, et al Multiplexed dpliced-leader sequencing: a high-throughput, selective method for RNA-seq in trypanosomatids. Sci Rep. 2017;7(1):1–11. 10.1038/s41598-016-0028-x 28623350 PMC5473914

[17] Kumar N, Creasy T, Sun Y, Flowers M, Tallon LJ, Dunning Hotopp JC. Efficient subtraction of insect rRNA prior to transcriptome analysis of *Wolbachia*-*Drosophila* lateral gene transfer. BMC Res Notes. 2012;5:230 10.1186/1756-0500-5-230 22583543 PMC3424148

[18] Oyola SO, Gu Y, Manske M, Otto TD, O’Brien J, Alcock D, et al Efficient depletion of host DNA contamination in malaria clinical sequencing. J Clin Microbiol. 2013;51(3):745–51. 10.1128/JCM.02507-12 23224084 PMC3592063

[19] Feehery GR, Yigit E, Oyola SO, Langhorst BW, Schmidt VT, Stewart FJ, et al A method for selectively enriching microbial DNA from contaminating vertebrate host DNA. PLoS One. 2013;8(10):e76096 10.1371/journal.pone.0076096 24204593 PMC3810253

[20] Domagalska MA, Imamura H, Sanders M, Broeck FV den, Bhattarai NR, Vanaerschot M, et al Genomes of intracellular *Leishmania* parasites directly sequenced from patients. bioRxiv. 2019;676163. 10.1371/journal.pntd.0007900 PMC6932831 31830038

[21] Melnikov A, Galinsky K, Rogov P, Fennell T, Van Tyne D, Russ C, et al Hybrid selection for sequencing pathogen genomes from clinical samples. Genome Biol. 2011;12(8):R73 10.1186/gb-2011-12-8-r73 21835008 PMC3245613

[22] Schuenemann VJ, Singh P, Mendum TA, Krause-Kyora B, Jäger G, Bos KI, et al Genome-wide comparison of medieval and modern *Mycobacterium leprae*. Science. 2013;341(6142):179–83. 10.1126/science.1238286 23765279

[23] Metsky HC, Matranga CB, Wohl S, Schaffner SF, Freije CA, Winnicki SM, et al Zika virus evolution and spread in the Americas. Nature. 2017;546(7658):411–5. 10.1038/nature22402 28538734 PMC5563848

[24] Cowell AN, Loy DE, Sundararaman SA, Valdivia H, Fisch K, Lescano AG, et al Selective whole-genome amplification is a robust method that enables scalable whole-genome sequencing of *Plasmodium vivax* from unprocessed clinical samples. mBio. 2017;8(1):e02257–16. 10.1128/mBio.02257-16 28174312 PMC5296604

[25] Hintzsche JD, Robinson WA, Tan AC. A survey of computational tools to analyze and interpret whole exome sequencing data. Int J Genomics. 2016;2016:7983236 10.1155/2016/7983236 28070503 PMC5192301

[26] Gampawar P, Saba Y, Werner U, Schmidt R, Müller-Myhsok B, Schmidt H. Evaluation of the performance of AmpliSeq and SureSelect exome sequencing libraries for Ion Proton. Front Genet. 2019;10:856 10.3389/fgene.2019.00856 31608108 PMC6774276

[27] Nag S, Dalgaard MD, Kofoed P-E, Ursing J, Crespo M, Andersen LO, et al High throughput resistance profiling of *Plasmodium falciparum* infections based on custom dual indexing and Illumina next generation sequencing-technology. Sci Rep. 2017;7(1):2398 10.1038/s41598-017-02724-x 28546554 PMC5445084

[28] Balkenhol N, Cushman S, Storfer A, Waits L. Landscape Genetics: Concepts, Methods, Applications. John Wiley & Sons; 2015. 292 p.

[29] Momčilović S, Cantacessi C, Arsić-Arsenijević V, Otranto D, Tasić-Otašević S. Rapid diagnosis of parasitic diseases: current scenario and future needs. Clin Microbiol Infect. 2019;25(3):290–309. 10.1016/j.cmi.2018.04.028 29730224

[30] Arias A, Watson SJ, Asogun D, Tobin EA, Lu J, Phan MVT, et al Rapid outbreak sequencing of Ebola virus in Sierra Leone identifies transmission chains linked to sporadic cases. Virus Evol. 2016;2(1):vew016 10.1093/ve/vew016 28694998 PMC5499387

[31] Park J, Shin SY, Kim K, Park K, Shin S, Ihm C. Determining genotypic drug resistance by ion semiconductor sequencing with the Ion AmpliSeqTM TB Panel in multidrug-resistant *Mycobacterium tuberculosis* isolates. Ann Lab Med. 2018;38(4):316–23. 10.3343/alm.2018.38.4.316 29611381 PMC5895860

[32] Ferrario C, Milani C, Mancabelli L, Lugli GA, Turroni F, Duranti S, et al A genome-based identification approach for members of the genus *Bifidobacterium*. FEMS Microbiol Ecol. 2015;91(3):fiv009 10.1093/femsec/fiv009 25764568

[33] Makowsky R, Lhaki P, Wiener HW, Bhatta MP, Cullen M, Johnson DC, et al Genomic diversity and phylogenetic relationships of human papillomavirus 16 (HPV16) in Nepal. Infect Genet Evol. 2016;46:7–11. 10.1016/j.meegid.2016.10.004 27725301 PMC5136510

[34] Schwabl P. Genomics and spatial surveillance of Chagas disease and American visceral leishmaniasis. University of Glasgow (doctoral thesis) 2020 Available from: http://theses.gla.ac.uk/81448/1/2020schwablphd.pdf

[35] Brenière SF, Waleckx E, Barnabé C. Over six thousand *Trypanosoma cruzi* strains classified into discrete typing units (DTUs): attempt at an inventory. PLoS Negl Trop Dis. 2016;10(8):e0004792 10.1371/journal.pntd.0004792 27571035 PMC5003387

[36] Monteiro WM, Magalhães LKC, de Sá ARN, Gomes ML, Toledo MJ de O, Borges L, et al *Trypanosoma cruzi* IV causing outbreaks of acute Chagas disease and infections by different haplotypes in the Western Brazilian Amazonia. PloS One. 2012;7(7):e41284 10.1371/journal.pone.0041284 22848457 PMC3405119

[37] Ramírez JD, Montilla M, Cucunubá ZM, Floréz AC, Zambrano P, Guhl F. Molecular epidemiology of human oral Chagas disease outbreaks in Colombia. PLoS Negl Trop Dis. 2013;7(2):e2041 10.1371/journal.pntd.0002041 23437405 PMC3578743

[38] Flores-López CA, Machado CA. Analyses of 32 loci clarify phylogenetic relationships among *Trypanosoma cruzi* lineages and support a single hybridization prior to human contact. PLoS Negl Trop Dis. 2011;5(8):e1272 10.1371/journal.pntd.0001272 21829751 PMC3149036

[39] Grijalva MJ, Suarez-Davalos V, Villacis AG, Ocaña-Mayorga S, Dangles O. Ecological factors related to the widespread distribution of sylvatic *Rhodnius ecuadoriensis* populations in southern Ecuador. Parasit Vectors. 2012;5:17 10.1186/1756-3305-5-17 22243930 PMC3282634

[40] Nascimento JD, Rosa JA da, Salgado-Roa FC, Hernández C, Pardo-Diaz C, Alevi KCC, et al Taxonomical over splitting in the *Rhodnius prolixus* (Insecta: Hemiptera: Reduviidae) clade: are *R*. *taquarussuensis* (da Rosa *et al*., 2017) and *R*. *neglectus* (Lent, 1954) the same species? PLoS One. 2019;14(2):e0211285 10.1371/journal.pone.0211285 30730919 PMC6366742

[41] Velásquez-Ortiz N, Hernández C, Herrera G, Cruz-Saavedra L, Higuera A, Arias-Giraldo LM, et al *Trypanosoma cruzi* infection, discrete typing units and feeding sources among *Psammolestes arthuri* (Reduviidae: Triatominae) collected in eastern Colombia. Parasit Vectors. 2019;12(1):157 10.1186/s13071-019-3422-y 30961657 PMC6454608

[42] Caicedo-Garzón V, Salgado-Roa FC, Sánchez-Herrera M, Hernández C, Arias-Giraldo LM, García L, et al Genetic diversification of *Panstrongylus geniculatus* (Reduviidae: Triatominae) in northern South America. PLoS One. 2019;14(10):e0223963 10.1371/journal.pone.0223963 31622439 PMC6797096

[43] Carrasco HJ, Torrellas A, García C, Segovia M, Feliciangeli MD. Risk of *Trypanosoma cruzi* I (Kinetoplastida: Trypanosomatidae) transmission by *Panstrongylus geniculatus* (Hemiptera: Reduviidae) in Caracas (Metropolitan District) and neighboring states, Venezuela. Int J Parasitol. 2005;35(13):1379–84. 10.1016/j.ijpara.2005.05.003 16019006

[44] Carrasco HJ, Segovia M, Llewellyn MS, Morocoima A, Urdaneta-Morales S, Martínez C, et al Geographical distribution of *Trypanosoma cruzi* genotypes in Venezuela. PLoS Negl Trop Dis. 2012;6(6):e1707 10.1371/journal.pntd.0001707 22745843 PMC3383755

[45] Nakad Bechara CC, Londoño JC, Segovia M, Sanchez MAL, Martínez PCE, Rodríguez RMM, Carrasco HJ. Genetic variability of *Panstrongylus geniculatus* (Reduviidae: Triatominae) in the Metropolitan District of Caracas, Venezuela. Infect Genet Evol. 2018;66:236–44. 10.1016/j.meegid.2018.09.011 30240833

[46] Messenger LA, Yeo M, Lewis MD, Llewellyn MS, Miles MA. Molecular genotyping of *Trypanosoma cruzi* for lineage assignment and population genetics. Methods Mol Biol. 2015;1201:297–337. 10.1007/978-1-4939-1438-8_19 25388123

[47] Li H, Durbin R. Fast and accurate short read alignment with Burrows-Wheeler transform. Bioinformatics. 2009;25(14):1754–60. 10.1093/bioinformatics/btp324 19451168 PMC2705234

[48] DePristo MA, Banks E, Poplin R, Garimella KV, Maguire JR, Hartl C, et al A framework for variation discovery and genotyping using next-generation DNA sequencing data. Nat Genet. 2011;43(5):491–8. 10.1038/ng.806 21478889 PMC3083463

[49] Derrien T, Estellé J, Marco Sola S, Knowles DG, Raineri E, Guigó R, et al Fast computation and applications of genome mappability. PLoS One. 2012;7(1):e3037 10.1371/journal.pone.0030377 22276185 PMC3261895

[50] Franzén O, Talavera-López C, Ochaya S, Butler CE, Messenger LA, Lewis MD, et al Comparative genomic analysis of human infective *Trypanosoma cruzi* lineages with the bat-restricted subspecies *T*. *cruzi marinkellei*. BMC Genomics. 2012;13:531 10.1186/1471-2164-13-531 23035642 PMC3507753

[51] Li L, Stoeckert CJ, Roos DS. OrthoMCL: identification of ortholog groups for eukaryotic genomes. Genome Res. 2003;13(9):2178–89. 10.1101/gr.1224503 12952885 PMC403725

[52] Talavera-Lopez C, Messenger LA, Lewis MD, Yeo M, Reis-Cunha JL, Bartholomeu DC, et al Repeat-driven generation of antigenic diversity in a major human pathogen, *Trypanosoma cruzi*. bioRxiv. 2018;283531. 10.3389/fcimb.2021.614665 PMC7966520 33747978

[53] You FM, Huo N, Gu YQ, Luo M-C, Ma Y, Hane D, et al BatchPrimer3: a high throughput web application for PCR and sequencing primer design. BMC Bioinformatics. 2008;9:253 10.1186/1471-2105-9-253 18510760 PMC2438325

[54] Kaplinski L, Andreson R, Puurand T, Remm M. MultiPLX: automatic grouping and evaluation of PCR primers. Bioinformatics. 2005;21(8):17012 10.1093/bioinformatics/bti219 15598831

[55] Sonnhammer EL, Hollich V. Scoredist: a simple and robust protein sequence distance estimator. BMC Bioinformatics. 2005;6:108 10.1186/1471-2105-6-108 15857510 PMC1131889

[56] Paradis E, Claude J, Strimmer K. APE: analyses of phylogenetics and evolution in R language. Bioinformatics. 2004;20(2):289–90. 10.1093/bioinformatics/btg412 14734327

[57] R: The R Project for Statistical Computing. Available from: https://www.r-project.org/

[58] Cummings KL, Tarleton RL. Rapid quantitation of *Trypanosoma cruzi* in host tissue by real-time PCR. Mol Biochem Parasitol. 2003;129(1):53–9. 10.1016/s0166-6851(03)00093-8 12798506

[59] Access Array System for Illumina Sequencing Systems. Available from: https://docplayer.net/78505463-Access-array-system-for-illumina-sequencing-systems.html

[60] Schmieder R, Edwards R. Fast identification and removal of sequence contamination from genomic and metagenomic datasets. PloS One. 2011;6(3):e17288 10.1371/journal.pone.0017288 21408061 PMC3052304

[61] Danecek P, Auton A, Abecasis G, Albers CA, Banks E, DePristo MA, et al The variant call format and VCFtools. Bioinformatics. 2011;27(15):2156–8. 10.1093/bioinformatics/btr330 21653522 PMC3137218

[62] Bandelt HJ, Forster P, Röhl A. Median-joining networks for inferring intraspecific phylogenies. Mol Biol Evol. 1999;16(1):37–48. 10.1093/oxfordjournals.molbev.a026036 10331250

[63] Leigh JW and Bryant D. PopART: full-feature software for haplotype network construction. Methods Ecol Evol. 2015;6:1110–16.

[64] Purcell S, Neale B, Todd-Brown K, Thomas L, Ferreira MAR, Bender D, et al PLINK: a tool set for whole-genome association and population-based linkage analyses. Am J Hum Genet. 2007;81(3):559–75. 10.1086/519795 17701901 PMC1950838

[65] Ritland K. Inferences about inbreeding depression based on changes of the inbreeding coefficient. Evolution. 1990;44(5):1230–41. 10.1111/j.1558-5646.1990.tb05227.x 28563887

[66] Wigginton JE, Cutler DJ, Abecasis GR. A note on exact tests of Hardy-Weinberg equilibrium. Am J Hum Genet. 2005;76(5):887–93. 10.1086/429864 15789306 PMC1199378

[67] Excoffier L, Lischer HEL. Arlequin suite ver 3.5: a new series of programs to perform population genetics analyses under Linux and Windows. Mol Ecol Resour. 2010;10(3):564–7. 10.1111/j.1755-0998.2010.02847.x 21565059

[68] Oksanen J, Blanchet FG, Friendly M, Kindt R, Legendre P, McGlinn D, et al. vegan: community ecology package. Available from: https://CRAN.R-project.org/package=vegan

[69] Šavrič B, Jenny B, Jenny H. Projection wizard–an online map projection selection tool. Cartogr J. 2016;53(2):177–85.

[70] Slatkin M. Isolation by distance in equilibrium and non-equilibrium populations. Evol Int J Org Evol. 1993;47(1):264–79. 10.1111/j.1558-5646.1993.tb01215.x 28568097

[71] Zumaya-Estrada FA, Messenger LA, Lopez-Ordonez T, Lewis MD, Flores-Lopez CA, Martínez-Ibarra AJ, et al North American import? Charting the origins of an enigmatic *Trypanosoma cruzi* domestic genotype. Parasit Vectors. 2012;5:226 10.1186/1756-3305-5-226 23050833 PMC3481457

[72] Ocaña-Mayorga S, Llewellyn MS, Costales JA, Miles MA, Grijalva MJ. Sex, subdivision, and domestic dispersal of *Trypanosoma cruzi* lineage I in southern Ecuador. PLoS Negl Trop Dis. 2010;4(12):e915 10.1371/journal.pntd.0000915 21179502 PMC3001902

[73] Messenger LA, Garcia L, Vanhove M, Huaranca C, Bustamante M, Torrico M, et al Ecological host fitting of *Trypanosoma cruzi* TcI in Bolivia: mosaic population structure, hybridization and a role for humans in Andean parasite dispersal. Mol Ecol. 2015;24(10):2406–22. 10.1111/mec.13186 25847086 PMC4737126

[74] Ramírez JD, Guhl F, Messenger LA, Lewis MD, Montilla M, Cucunuba Z, et al Contemporary cryptic sexuality in *Trypanosoma cruzi*. Mol Ecol. 2012;21(17):4216–26. 10.1111/j.1365-294X.2012.05699.x 22774844

[75] Llewellyn MS, Lewis MD, Acosta N, Yeo M, Carrasco HJ, Segovia M, et al *Trypanosoma cruzi* IIc: phylogenetic and phylogeographic insights from sequence and microsatellite analysis and potential impact on emergent Chagas disease. PLoS Negl Trop Dis. 2009;3(9):e510 10.1371/journal.pntd.0000510 19721699 PMC2727949

[76] Roman F, Xavier S das C, Messenger LA, Pavan MG, Miles MA, Jansen AM, et al Dissecting the phyloepidemiology of *Trypanosoma cruzi* I (TcI) in Brazil by the use of high resolution genetic markers. PLoS Negl Trop Dis. 2018;12(5):e0006466 10.1371/journal.pntd.0006466 29782493 PMC5983858

[77] Barnabe C, Buitrago R, Bremond P, Aliaga C, Salas R, Vidaurre P, et al Putative panmixia in restricted populations of *Trypanosoma cruzi* isolated from wild *Triatoma infestans* in Bolivia. PloS One. 2013;8(11):e82269 10.1371/journal.pone.0082269 24312410 PMC3843716

[78] Llewellyn MS. The molecular epidemiology of *Trypanosoma cruzi* infection in wild and domestic transmission cycles with special emphasis on multilocus microsatellite analysis. London School of Hygiene & Tropical Medicine (doctoral thesis). 2008 Available from: https://researchonline.lshtm.ac.uk/id/eprint/4652860/

[79] Lewis MD, Llewellyn MS, Yeo M, Acosta N, Gaunt MW, Miles MA. Recent, independent and anthropogenic origins of *Trypanosoma cruzi* hybrids. PLoS Negl Trop Dis. 2011; 5(10):e1363 10.1371/journal.pntd.0001363 22022633 PMC3191134

[80] Shibata H, Rai SK, Satoh M, Murakoso K, Sumi K, Uga S, et al The use of PCR in detecting toxoplasma parasites in the blood and brains of mice experimentally infected with *Toxoplasma gondii*. Kansenshogaku Zasshi. 1995;69(2):158–63. 10.11150/kansenshogakuzasshi1970.69.158 7745290

[81] Yang H, Golenberg EM, Shoshani J. Proboscidean DNA from museum and fossil specimens: an assessment of ancient DNA extraction and amplification techniques. Biochem Genet. 1997;35(5):165–79. 10.1023/a:1021902125382 9332711

[82] Ramos RAN, Ramos CAN, Santos EMS, de Araújo FR, de Carvalho GA, Faustino MAG, et al Quantification of *Leishmania infantum* DNA in the bone marrow, lymph node and spleen of dogs. Rev Bras Parasitol Vet. 2013;22(3):346–50. 10.1590/S1984-29612013000300005 24142164

[83] Schubert G, Stockhausen M, Hoffmann C, Merkel K, Vigilant L, Leendertz F, et al Targeted detection of mammalian species using carrion fly–derived DNA. Mol Ecol Resour. 2015;15(2):285–94. 10.1111/1755-0998.12306 25042567

[84] Côté NML, Daligault J, Pruvost M, Bennett EA, Gorgé O, Guimaraes S, et al A new high-throughput approach to genotype ancient human gastrointestinal parasites. PLoS One. 2016 11(1):e0146230 10.1371/journal.pone.0146230 26752051 PMC4709038

[85] Cencig S, Coltel N, Truyens C, Carlier Y. Parasitic loads in tissues of mice infected with *Trypanosoma cruzi* and treated with AmBisome. PLoS Negl Trop Dis. 2011;5(6):e1216 10.1371/journal.pntd.0001216 21738811 PMC3125148

[86] Thompson CT, Dvorak JA. Quantitation of total DNA per cell in an exponentially growing population using the diphenylamine reaction and flow cytometry. Anal Biochem. 1989; 177(2):353–7. 10.1016/0003-2697(89)90065-1 2658678

[87] Reithinger R, Lambson BE, Barker DC, Davies CR. Use of PCR to detect *Leishmania* (*Viannia*) spp. in dog blood and bone marrow. 2000;38(2):748–51. 10.1128/JCM.38.2.748-751.2000 10655379 PMC86194

[88] Wen C, Wu L, Qin Y, Van Nostrand JD, Ning D, Sun B, et al Evaluation of the reproducibility of amplicon sequencing with Illumina MiSeq platform. PLoS One.2017;12(4):e0176716 10.1371/journal.pone.0176716 28453559 PMC5409056

[89] Storfer A, Patton A, Fraik AK. Navigating the interface between landscape genetics and landscape genomics. Front Genet. 2018;13;9:68 10.3389/fgene.2018.00068 29593776 PMC5859105

[90] Erben ED. High-throughput methods for dissection of trypanosome gene regulatory networks. Curr Genomics. 2018;19(2):78–86. 10.2174/1389202918666170815125336 29491736 PMC5814965

[91] Cingolani P, Platts A, Wang LL, Coon M, Nguyen T, Wang L, et al A program for annotating and predicting the effects of single nucleotide polymorphisms, SnpEff: SNPs in the genome of *Drosophila melanogaster* strain w1118; iso-2; iso-3. Fly. 2012;6(2):80–92. 10.4161/fly.19695 22728672 PMC3679285

[92] Quinlan AR, Hall IM. BEDTools: a flexible suite of utilities for comparing genomic features. Bioinformatics. 2010;26(6):841–2. 10.1093/bioinformatics/btq033 20110278 PMC2832824

[93] Aurrecoechea C, Barreto A, Basenko EY, Brestelli J, Brunk BP, Cade C, et al EuPathDB: the eukaryotic pathogen genomics database resource. Nucleic Acids Res. 2017;45(database issue):D581–D591. 10.1093/nar/gkw1105 27903906 PMC5210576

[94] Linck E, Battey CJ. Minor allele frequency thresholds strongly affect population structure inference with genomic data sets. Mol Ecol Resour. 2019;19(3):639–47. 10.1111/1755-0998.12995 30659755

[95] Excoffier L, Dupanloup I, Huerta-Sánchez E, Sousa VC, Foll M. Robust demographic inference from genomic and SNP data. PLoS Genet. 2013;9(10):e1003905 10.1371/journal.pgen.1003905 24204310 PMC3812088

[96] Bryant D, Bouckaert R, Felsenstein J, Rosenberg NA, RoyChoudhury A. Inferring species trees directly from biallelic genetic markers: bypassing gene trees in a full coalescent analysis. Mol Biol Evol. 2012;29(8):1917–32. 10.1093/molbev/mss086 22422763 PMC3408069

[97] Landguth EL, Bearlin A, Day CC, Dunham J. CDMetaPOP: an individual-based, eco-evolutionary model for spatially explicit simulation of landscape demogenetics. Methods Ecol Evol. 2017;8(1):4–11.

[98] Pritchard JK, Stephens M, Donnelly P. Inference of population structure using multilocus genotype data. Genetics. 2000;155(2):945–59. 10835412 10.1093/genetics/155.2.945 PMC1461096

[99] Piry S, Alapetite A, Cornuet J-M, Paetkau D, Baudouin L, Estoup A. GENECLASS2: a software for genetic assignment and first-generation migrant detection. J Hered. 2004;95(6):536–9. 10.1093/jhered/esh074 15475402

[100] Cheng L, Connor TR, Sirén J, Aanensen DM, Corander J. Hierarchical and spatially explicit clustering of DNA sequences with BAPS software. Mol Biol Evol. 2013;30(5):1224–8. 10.1093/molbev/mst028 23408797 PMC3670731

[101] Anderson EC, Thompson EA. A model-based method for identifying species hybrids using multilocus genetic data. Genetics. 2002;160(3):1217–29. 11901135 10.1093/genetics/160.3.1217 PMC1462008

[102] Graffelman J, Jain D, Weir B. A genome-wide study of Hardy–Weinberg equilibrium with next generation sequence data. Hum Genet. 2017;136(6):727–41. 10.1007/s00439-017-1786-7 28374190 PMC5429372

[103] Sefid Dashti MJ, Gamieldien J. A practical guide to filtering and prioritizing genetic variants. BioTechniques. 2017;62(1):18–30. 10.2144/000114492 28118812

[104] Etherington TR. Python based GIS tools for landscape–genetics: visualising genetic relatedness and measuring landscape connectivity. Methods Ecol Evol. 2011;2:52–5.

[105] Carrasco HJ, Segovia M, Londoño JC, Ortegoza J, Rodríguez M, Martínez CE. *Panstrongylus geniculatus* and four other species of triatomine bug involved in the *Trypanosoma cruzi* enzootic cycle: high risk factors for Chagas’ disease transmission in the Metropolitan District of Caracas, Venezuela. Parasit Vectors. 2014;7:602 10.1186/s13071-014-0602-7 25532708 PMC4307744

[106] Zingales B. *Trypanosoma cruzi* genetic diversity: something new for something known about Chagas disease manifestations, serodiagnosis and drug sensitivity. Acta Trop. 2018;184:38–52. 10.1016/j.actatropica.2017.09.017 28941731

[107] Nunes Maria Carmo Pereira, Beaton Andrea, Acquatella Harry, Bern Caryn, Bolger Ann F., Echeverría Luis E., et al Chagas cardiomyopathy: an update of current clinical knowledge and management: a scientific statement from the American Heart Association. Circulation. 2018;138(12):e169–209. 10.1161/CIR.0000000000000599 30354432

[108] Llewellyn MS, Rivett-Carnac JB, Fitzpatrick S, Lewis MD, Yeo M, Gaunt MW, et al Extraordinary *Trypanosoma cruzi* diversity within single mammalian reservoir hosts implies a mechanism of diversifying selection. Int J Parasitol. 2011;41(6–10):609–14. 10.1016/j.ijpara.2010.12.004 21232539 PMC3084450

[109] Valadares HMS, Pimenta JR, Segatto M, Veloso VM, Gomes ML, Chiari E, et al Unequivocal identification of subpopulations in putative multiclonal *Trypanosoma cruzi* strains by FACs single cell sorting and genotyping. PLoS Negl Trop Dis. 2012;6(7):e1722 10.1371/journal.pntd.0001722 22802979 PMC3393670

[110] Pronovost H, Peterson AC, Chavez BG, Blum MJ, Dumonteil E, Herrera CP. Deep sequencing reveals multiclonality and new discrete typing units of *Trypanosoma cruzi* in rodents from the southern United States. J Microbiol Immunol Infect. 2018;S1684-1182(18)30097–5. 10.1016/j.jmii.2018.12.004 30709717

[111] Yeo M, Lewis MD, Carrasco HJ, Acosta N, Llewellyn M, da Silva Valente SA, et al Resolution of multiclonal infections of *Trypanosoma cruzi* from naturally infected triatomine bugs and from experimentally infected mice by direct plating on a sensitive solid medium. Int J Parasitol. 2007;37(1):111–20. 10.1016/j.ijpara.2006.08.002 17052720

[112] Baptista RP, Reis-Cunha JL, DeBarry JD, Chiari E, Kissinger JC, Bartholomeu DC, et al Assembly of highly repetitive genomes using short reads: the genome of discrete typing unit III *Trypanosoma cruzi* strain 231. Microb Genomics. 2018;4(4):e000156 10.1099/mgen.0.000156 29442617 PMC5989580

