# Supplementary figures and images for: Culture-free genome-wide locus sequence typing (GLST) provides new perspectives on Trypanosoma cruzi dispersal and infection complexity
Source: PLoS Genet. 2020 Dec 16;16(12):e1009170. doi: 10.1371/journal.pgen.1009170 (PMC7743988; doi:10.1371/journal.pgen.1009170)

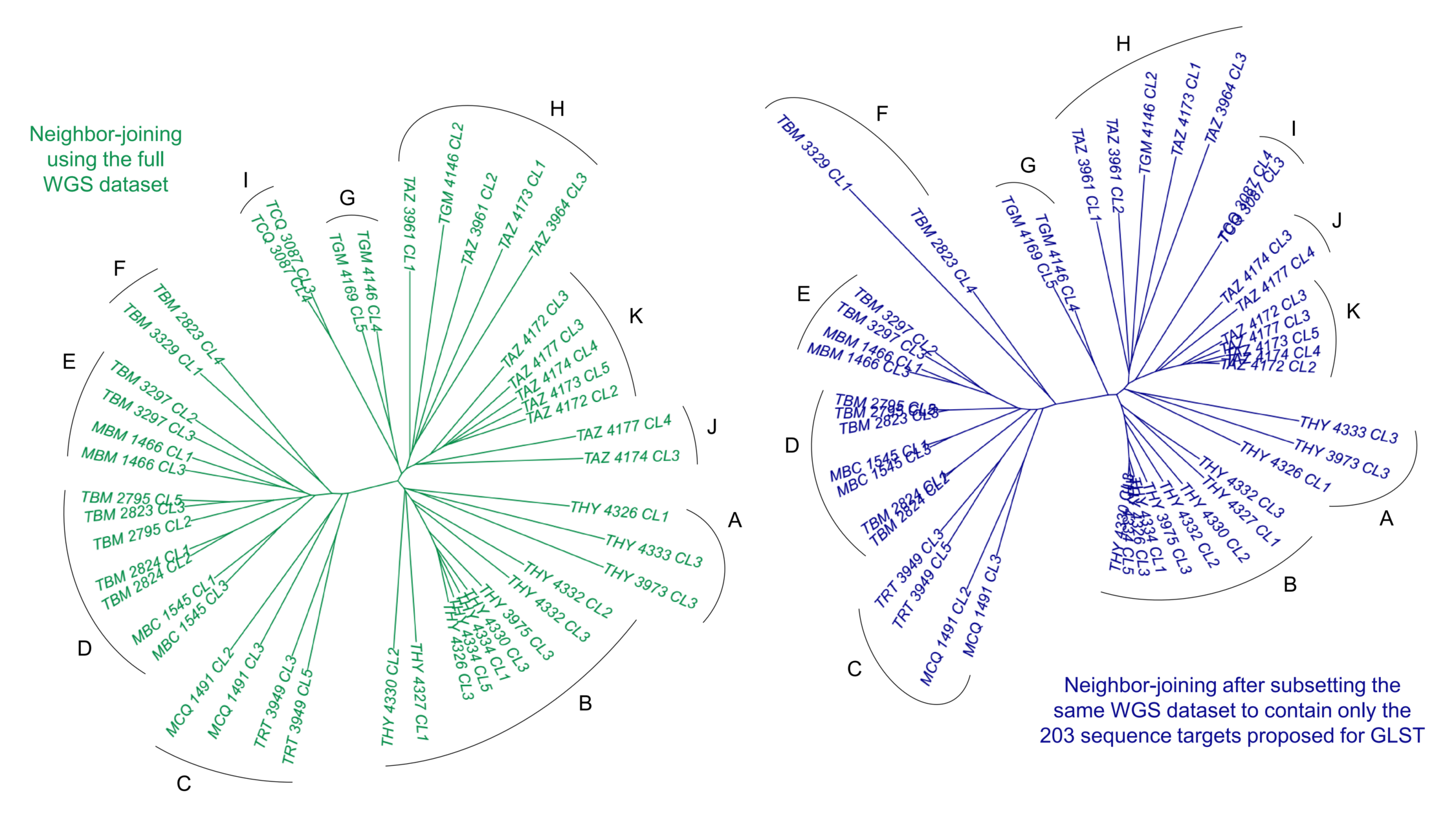

Supplement: S1 Fig — The green tree shows neighbor-joining (NJ) relationships calculated from 106,007 SNP sites identified from whole-genome sequencing (WGS) of 45 TcI clones in southern Ecuador [1]. Sites missing genotypes in ≥ 10% individuals are excluded. Less than 45 km separate the most distant sampling sites within the study region. Several pairs of clones also represent the same host/vector individual (see first seven characters of IDs). NJ was repeated after abridging the WGS dataset to contain only SNPs within the 203 sequence targets proposed by GLST (also excluding sites missing ≥ 10% genotypes). This resultant tree (blue, at right) uses 391 SNP sites and recreates clusters A–K observed in WGS. (TIF) [file pgen.1009170.s001.tif]

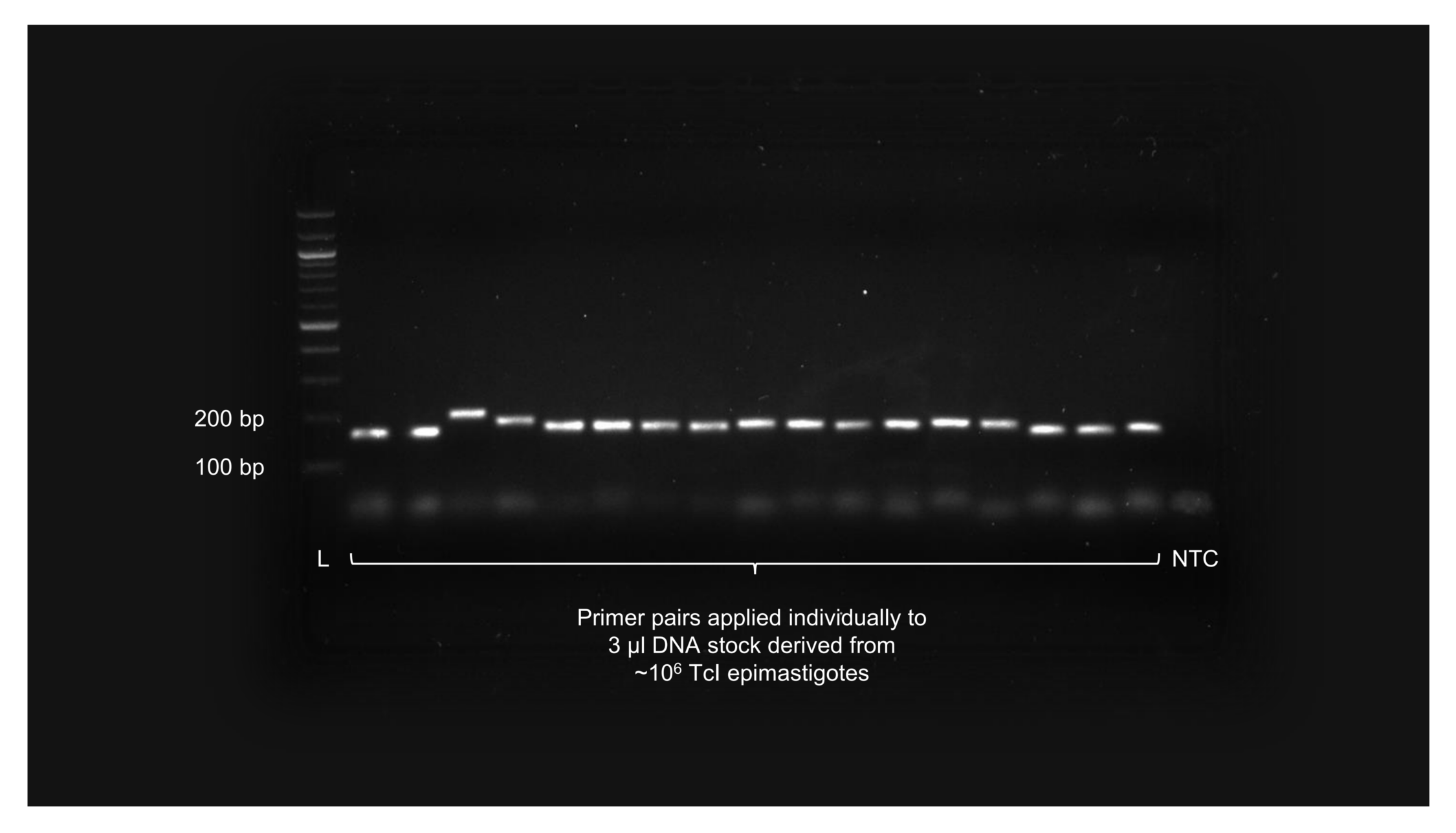

Supplement: S2 Fig — Primer pairs were first applied individually to pure TcI epimastigote DNA to confirm product amplification within the expected size range (164–204 bp). The figure shows the electrophoresed products of 17 different primer pairs in 0.8% agarose gel as well as DNA ladder (L) and no-template control (NTC). All other primer pairs achieved similar results using an initial incubation step at 98°C (2 min); 30 amplification cycles at 98°C (10 s), 60°C (30 s) and 72°C (45 s); and a final extension step at 72°C (2 min). (TIF) [file pgen.1009170.s002.tif]

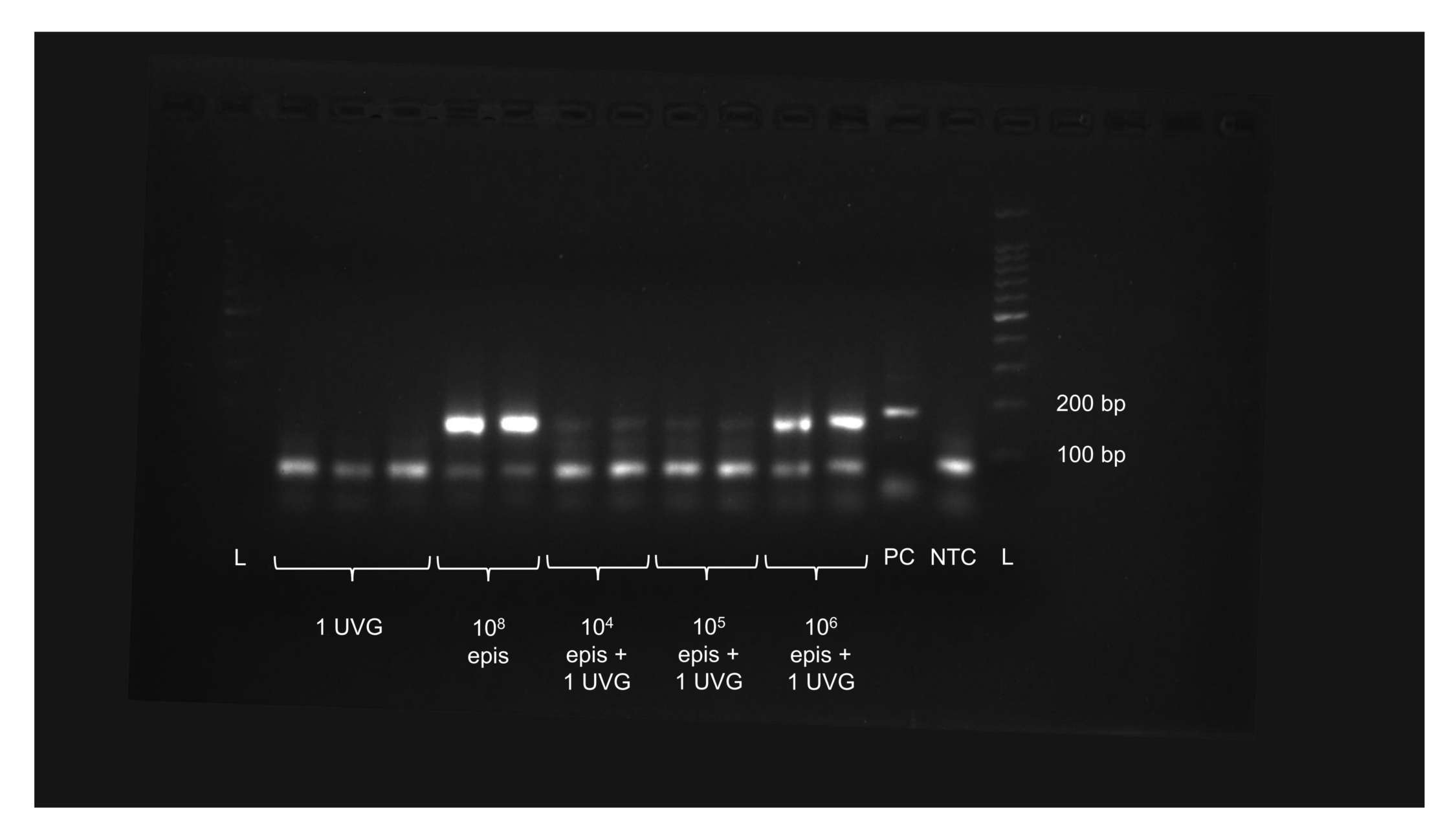

Supplement: S3 Fig — We created mock infections by mixing 104, 105 and 106 RNAlater-preserved TcI-Sylvio epimastigote (epi) cells with uninfected R. prolixus vector gut (UVG). DNA extracted from these mock infections was subjected to the multiplexed, 203-target GLST reaction (using the same cycling conditions as for single-target reactions–see Methods or S2 Fig legend) and products were electrophoresed in 0.8% agarose gel. Fainter banding of GLST products from lower concentration mock infections encouraged follow-up on sensitivity thresholds using additional dilution curves and qPCR. Next to DNA ladder (L) and no-template control (NTC), the gel also contains TcZ primer product from pure TcI epimastigote DNA. TcZ primers provide a highly sensitive positive control (PC) as they target 195 bp satellite DNA repeats that make up ca. 5% of the T. cruzi genome. (TIF) [file pgen.1009170.s003.tif]

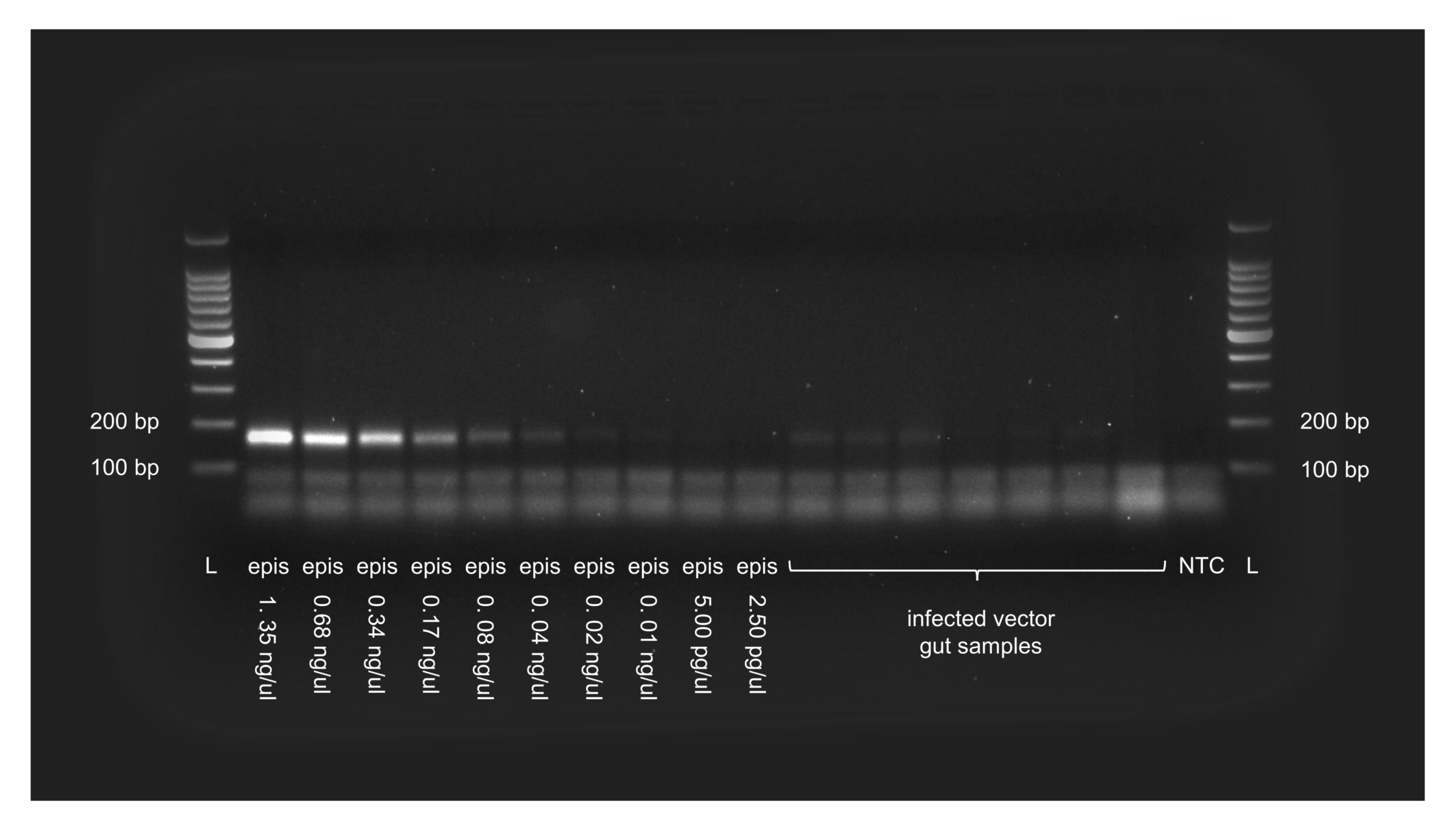

Supplement: S4 Fig — The left side shows electrophoresed GLST amplicons generated from 3 μl pure TcI epimastigote (epi) DNA with concentrations between 1.35 ng/μl and 2.50 pg/μl (see cycling conditions in Methods or S2 Fig legend). Lanes on the right contain amplicons from seven random metagenomic samples that tested positive for T. cruzi satellite DNA. DNA ladders (L) and no-template control (NTC) are indicated left and right. Poor amplicon visibility occurs at ≤ 30 pg epimastigote DNA input (3 μl). Gut DNA amplicon visibility is also limited but whether this relates to low T. cruzi content or amplification interference is unclear without qPCR. (TIF) [file pgen.1009170.s004.tif]

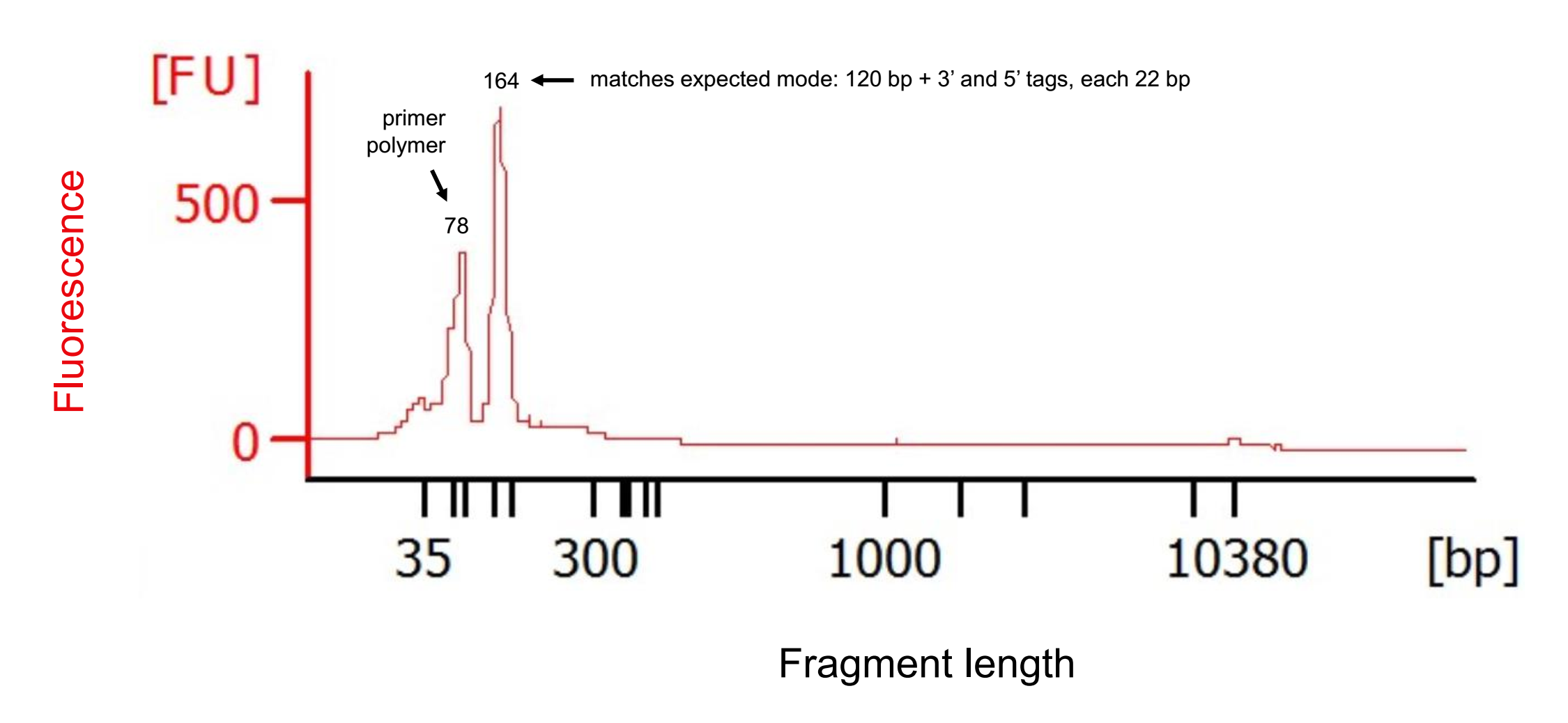

Supplement: S5 Fig — The figure plots fragment sizes (calculated based on migration times relative to those of standards) and fluorescence intensity (FU) of first-round PCR products (see cycling conditions in Methods or S2 Fig legend) measured with the Agilent Bioanalyzer 2100 System. The first peak represents primer polymerization that is removed in subsequent gel excision/re-solubilization steps. The second peak matches expectations for the multi-target GLST product (164–204 bp). (TIF) [file pgen.1009170.s005.tif]

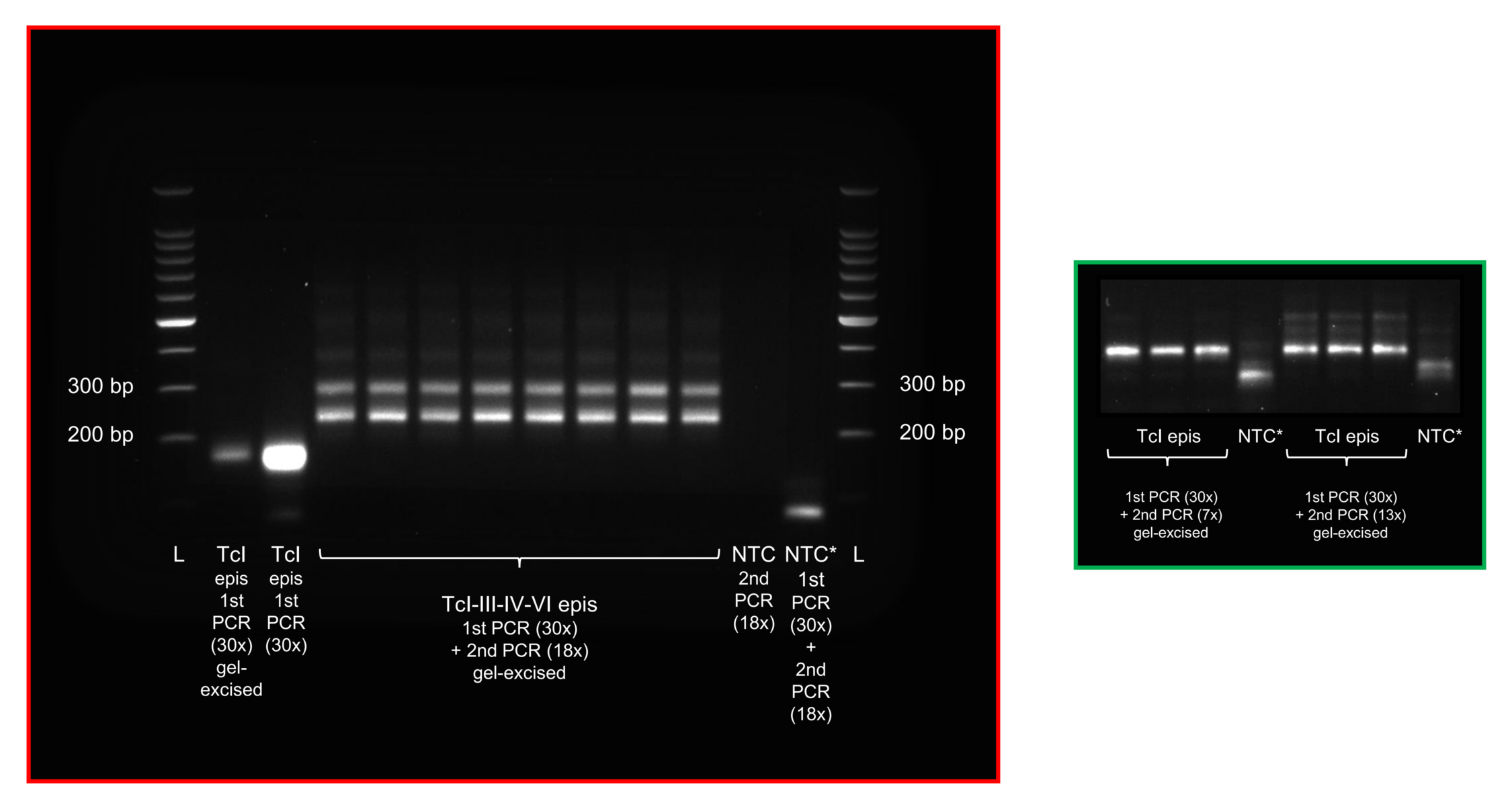

Supplement: S6 Fig — The second (barcoding) PCR reaction uses an initial incubation step at 98°C (2 min); 7 amplification cycles at 98°C (30 s), 60°C (30 s) and 72°C (1 min); and a final extension step at 72°C (3 min). Seven amplification cycles were chosen because unwanted polymers formed at 13 and 18x. The center lanes in the 0.8% agarose gel at left (red border) show electrophoresed GLST products from reference clones after eighteen cycles of barcoding PCR. Large, non-target banding occurs at ≥ 300 bp. Unbarcoded products from TcI epimastigote (epi) DNA are also shown at left. No template controls from barcoding (NTC) and first-round + barcoding PCR (NTC*) occur next to the DNA ladder (L) on the right side of the gel. The smaller image (green border) to the right shows how unwanted banding becomes less pronounced at 13x and largely disappears at 7x. This 0.8% agarose gel also contains NTC* samples, i.e., negative controls carried through both first and second-round PCR. (TIF) [file pgen.1009170.s006.tif]

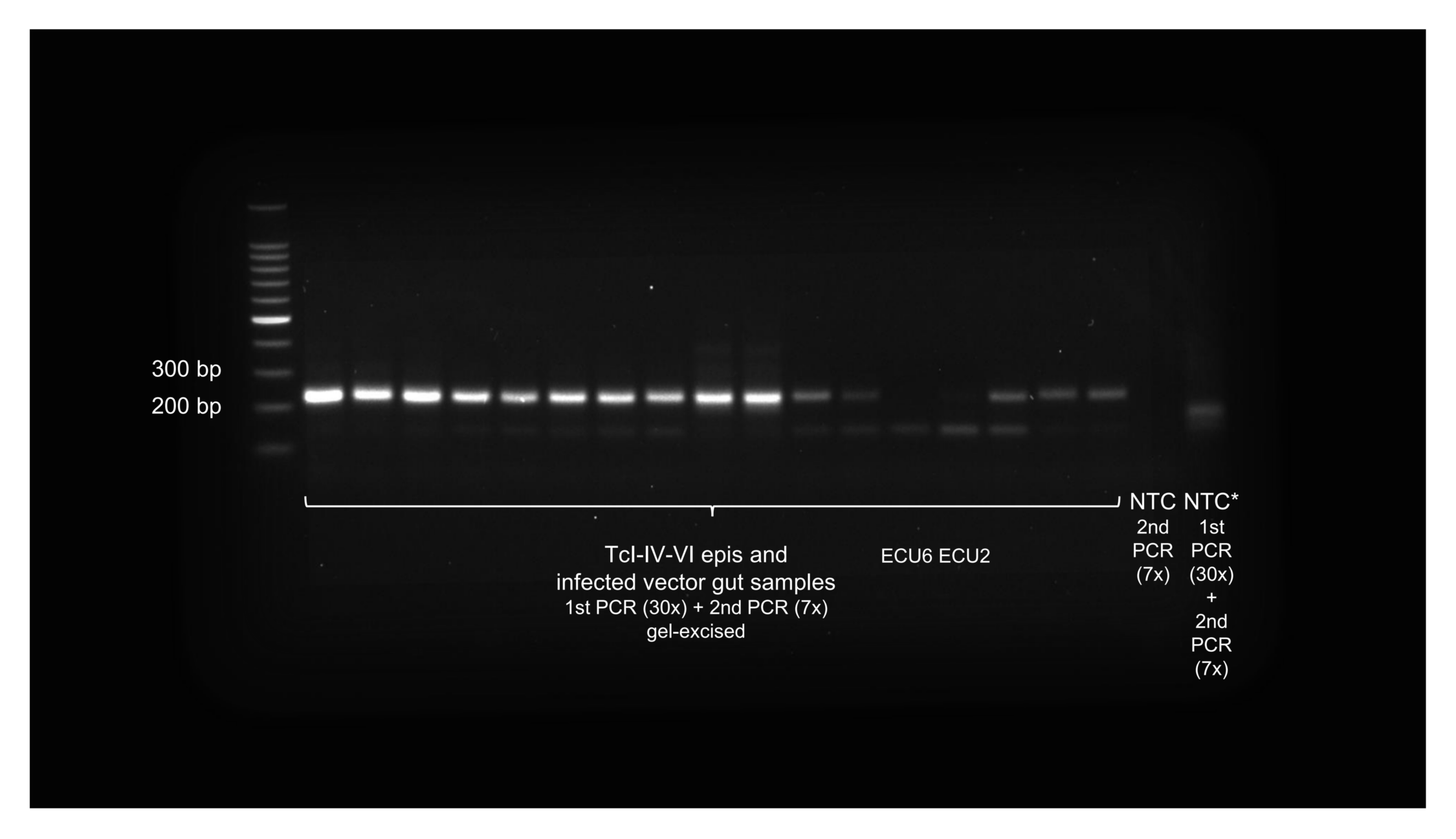

Supplement: S7 Fig — The 0.8% agarose gel shows a subset of fifteen GLST products from the second-round (barcoding) PCR reaction (see cycling conditions in Methods or S6 Fig legend) prior to equimolar pooling and final gel excision/re-solubilization steps. Products from ECU6 and ECU2 occur in this gel but were not included in the final pool. The gel also contains DNA ladder (L) and no-template controls from barcoding (NTC) and first-round + barcoding PCR (NTC*). (TIF) [file pgen.1009170.s007.tif]

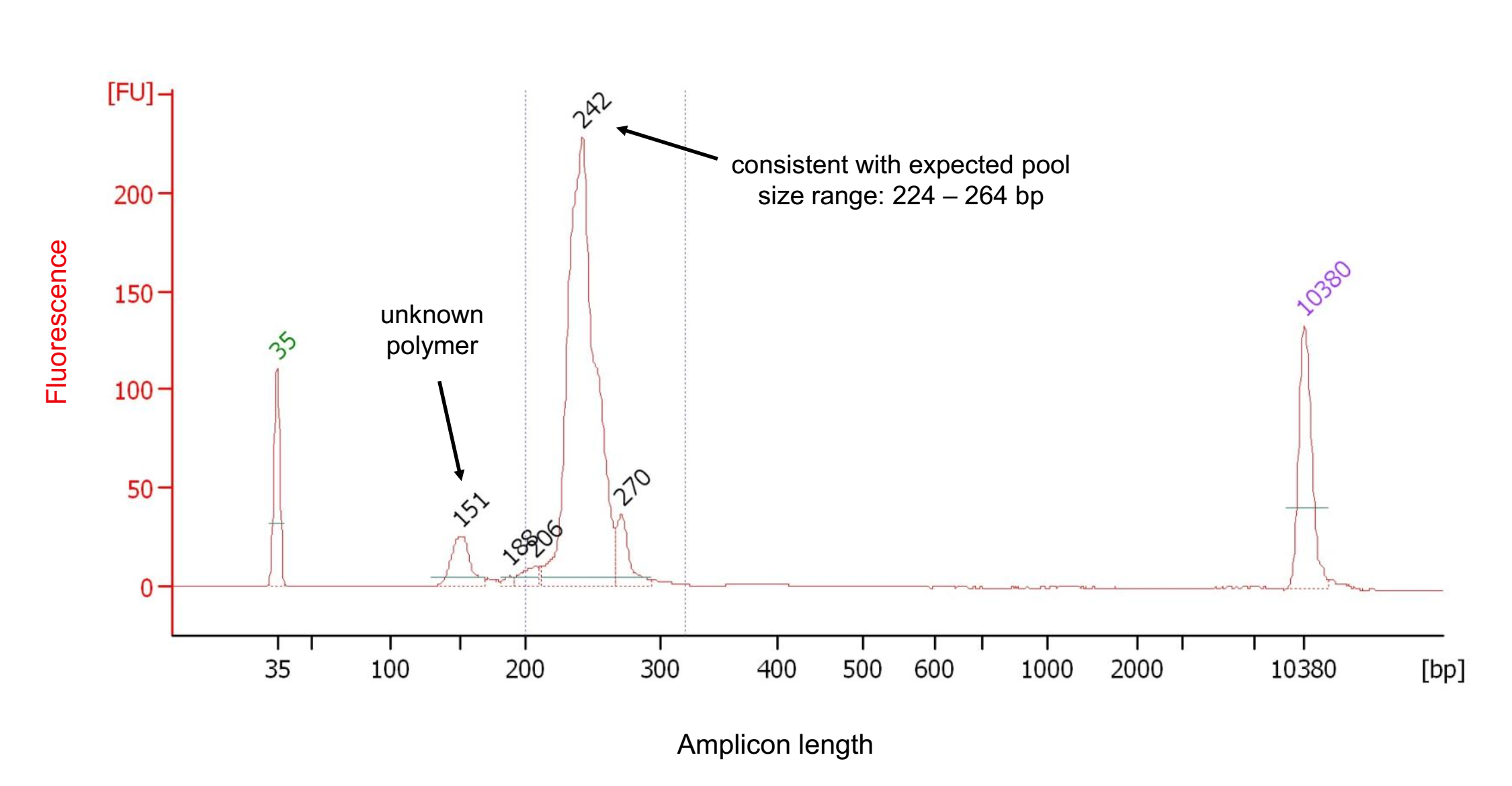

Supplement: S8 Fig — The figure plots fragment sizes (calculated based on migration times relative to those of standards) and fluorescence intensity (FU) of the final GLST pool measured with the Agilent Bioanalyzer 2100 System. The large peak matches expectations for the multi-target GLST product pool (224–264 bp). Left and right peaks labelled in green and purple represent standards of known size. A small non-target peak remaining near 151 bp encourages improvement of prior size selection steps. (TIF) [file pgen.1009170.s008.tif]

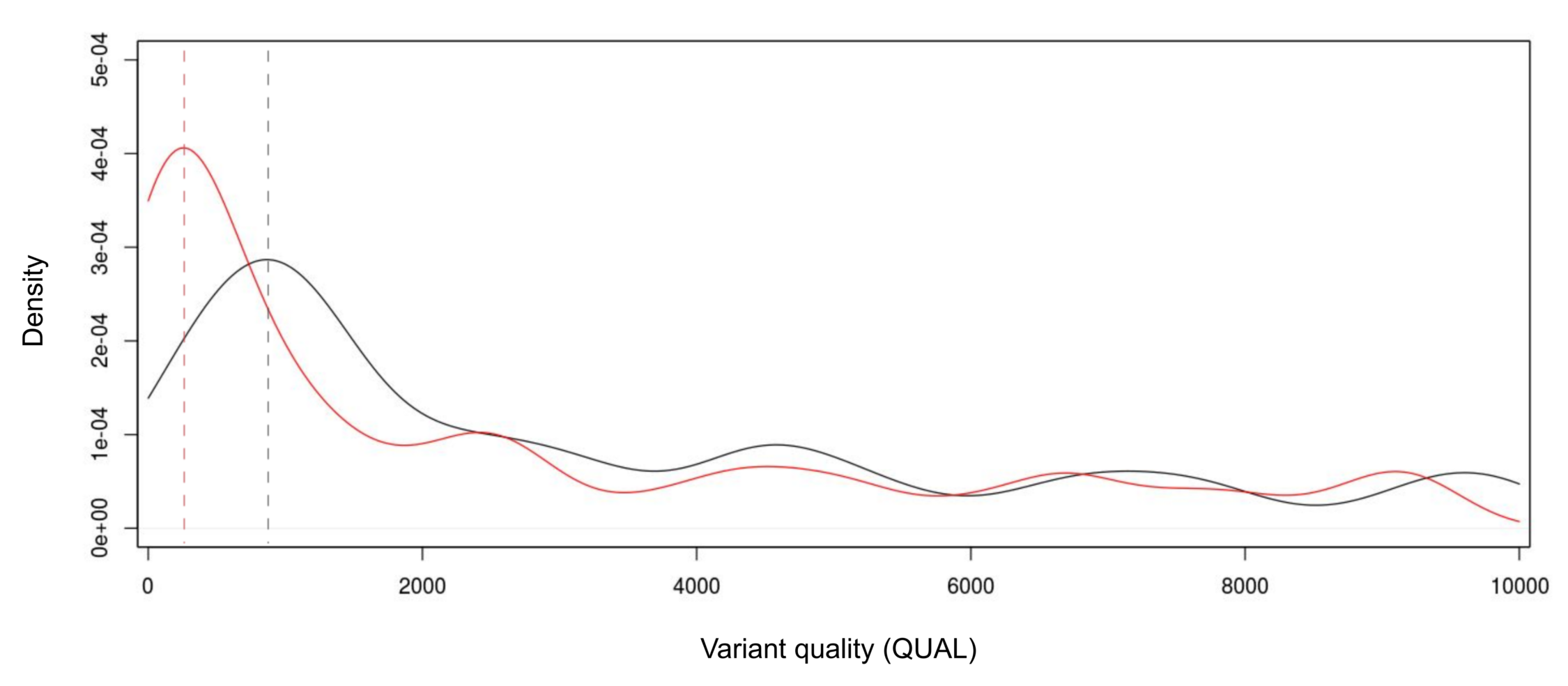

Supplement: S9 Fig — The GLST primer panel was designed based on single-nucleotide polymorphisms (SNPs) in Ecuadorian TcI clones. It was applied, however, to samples from distant geographic locations as well as to non-TcI clones. Additional, previously unidentified SNP sites (PU) were thus expected to be found but we needed to distinguish true PU from PCR and sequencing error. We reasoned that quality statistics (e.g., mapping quality, strand bias, minor allele frequency, etc.–see Methods) at previously identified SNP sites (PI) could help calibrate quality filters applied to the wider dataset. This strategy finds support in the above density plot of QUAL scores computed by GATK [48]. The plot suggests that, prior to variant filtration, lower QUAL scores occur more often at PU (red) than at PI (black). We thus imposed the most stringent filtering criteria possible without losing PI. (TIF) [file pgen.1009170.s009.tif]

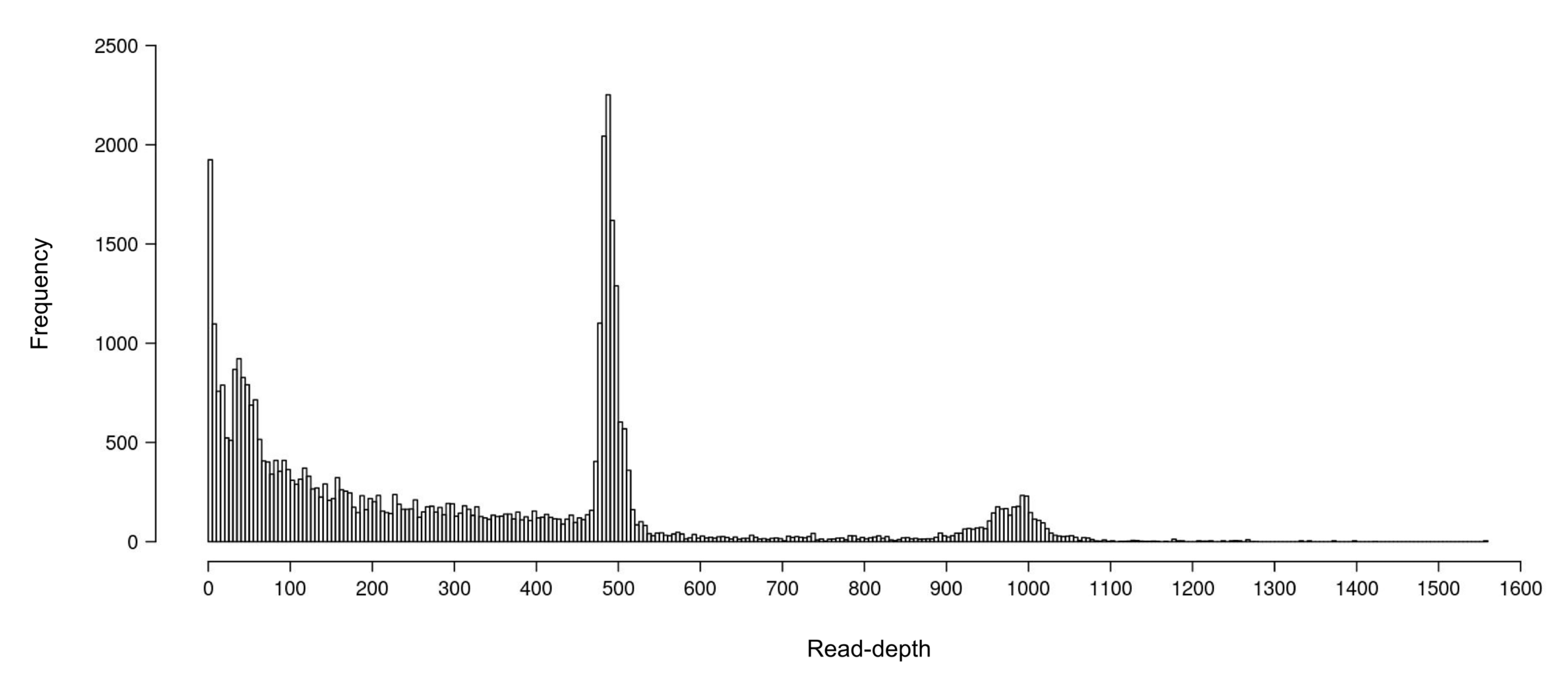

Supplement: S10 Fig — Median read-depth is 267x including zero-depth genotypes (6% of total) and 309x excluding zero-depth genotypes. (TIF) [file pgen.1009170.s010.tif]

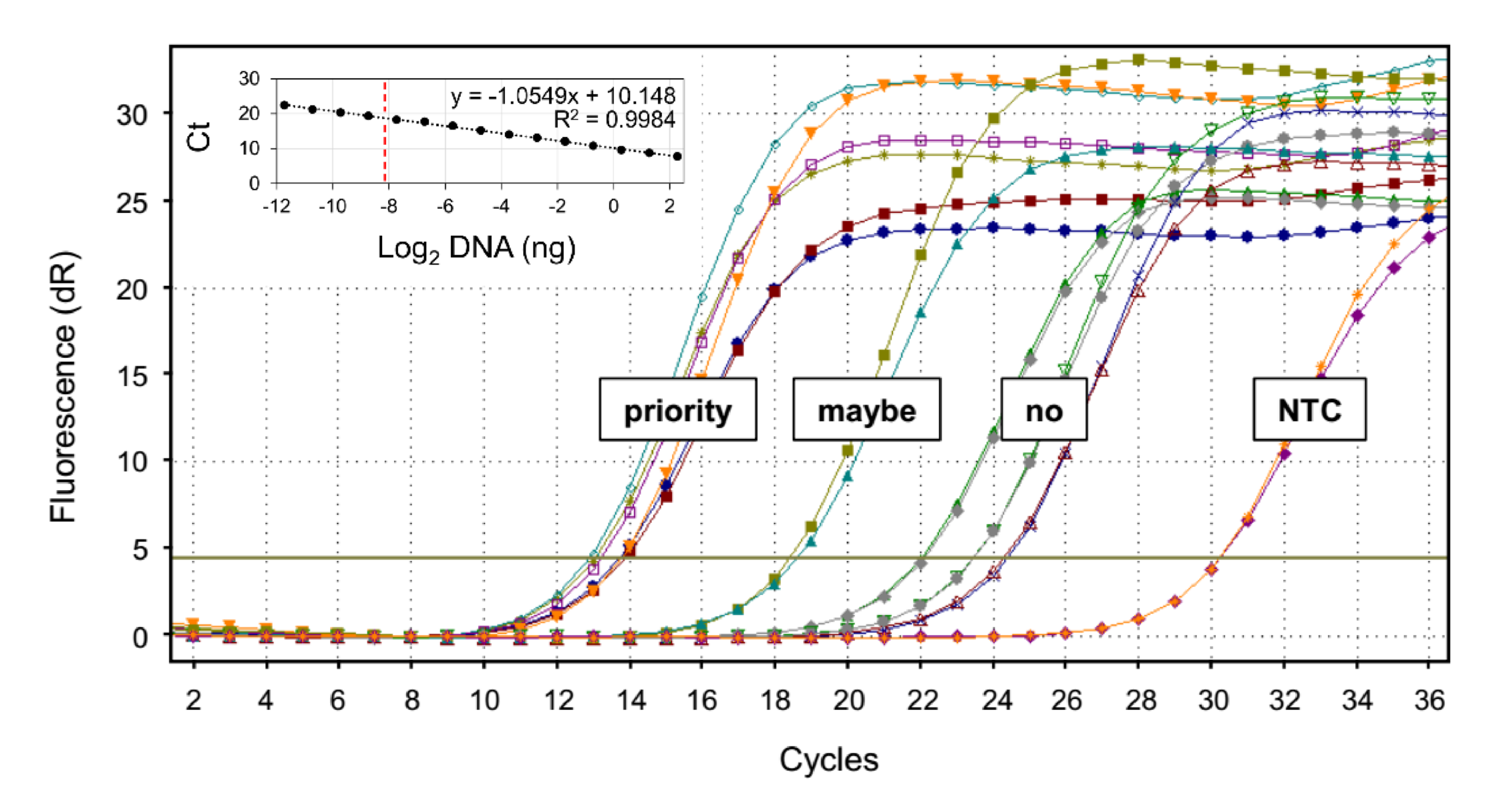

Supplement: S11 Fig — We used T. cruzi satellite DNA qPCR to identify vector gut samples with T. cruzi DNA quantities within ranges successfully visualized in GLST reactions using epimastigote DNA (S4 Fig). The qPCR reaction used an initial incubation step at 95°C (10 min) and 40 amplification cycles at 95°C (15 s), 55°C (15 s) and 72°C (15 s). The plot shows baseline-corrected fluorescence (dR) for seven sample duplicates. Following the regression equation from the standard curve (see inset), the three samples with highest cycle thresholds (Ct values) in this example represent gut extracts with 0.05 to 0.14 ng/μl T. cruzi DNA. Such samples with T. cruzi DNA concentrations above 0.01 ng/μl were prioritized for GLST and none failed in library construction. ECU36, with a mean Ct value of 18.68 in the plot, was also successfully sequenced. A Ct value of 18.68 represents 3.69 pg/μl T. cruzi DNA. Not all samples with concentrations at single-digit picogram levels (per μl) were successful and we did not troubleshoot those with substantially lower concentrations based on qPCR. (TIF) [file pgen.1009170.s011.tif]

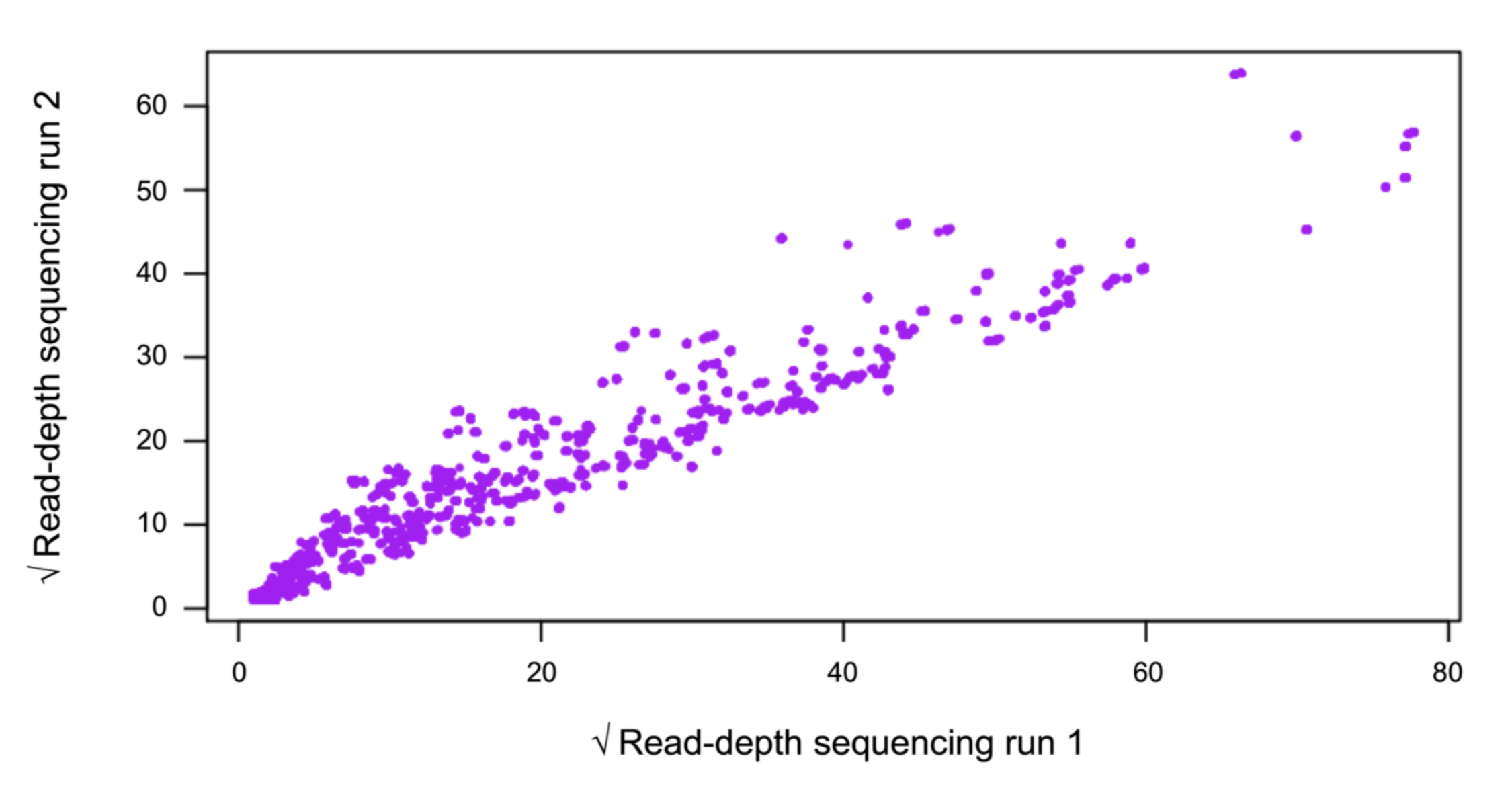

Supplement: S12 Fig — We sequenced the same GLST pool in two separate Illumina MiSeq runs. Run 1 involved GLST as a spike to a collaborator’s 16S amplicon library, whereby GLST reads were subsequently decontaminated from (barcode-sharing) 16S reads by alignment to the TcI-Sylvio reference genome. GLST libraries were sequenced alone in run 2. Read-depths at each GLST base position (purple points) are highly correlated between the two runs (Pearson's r = 0.93, p < 0.001). Run 1 had higher sequencing output than run 2. Values are square-root transformed and represent the control sample TBM_2975_CL2_rep1. (TIF) [file pgen.1009170.s012.tif]

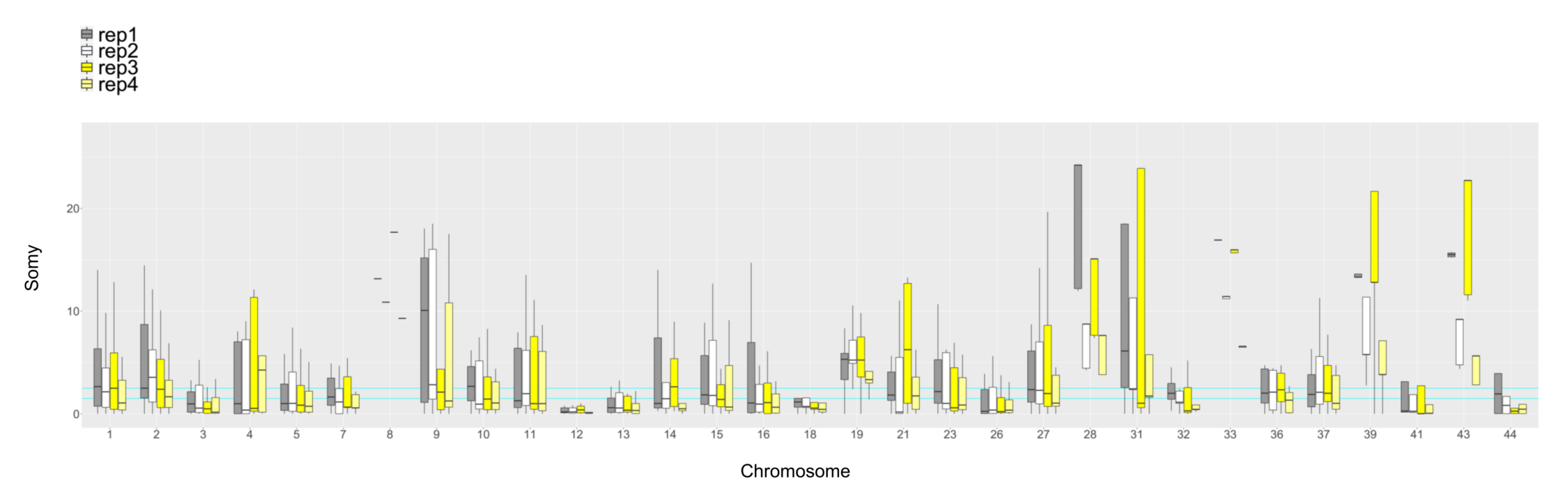

Supplement: S13 Fig — We adapted methods from Schwabl et al. 2019 [1] to derive somy estimates for each base position within GLST amplicons. Briefly, we calculated median-read-depth of all target bases for each chromosome. We let the median of these chromosomal medians (the ‘inter-chromosomal median’) represent expectations for the disomic state, estimating copy number per base position by dividing each position’s read-depth by the inter-chromosomal median and multiplying by two. Boxplots show median and interquartile ranges of these site-wise somy estimates for each chromosome in TBM_2975_CL2 control replicates. TBM_2795_CL2 did not show chromosomal amplifications in whole-genome analysis [1]. Not unexpectedly for a PCR-based method, somy values estimated from GLST read-depths differ substantially among replicates and are unrealistically high/low on many chromosomes. Estimates on chromosomes with few GLST targets appear especially unreliable–e.g., see chromosomes 8, 28, 33, 39 and 43. These chromosomes contain ≤ 2 GLST targets each. Horizontal cyan lines mark y = 1.5 and y = 2.5. (TIF) [file pgen.1009170.s013.tif]

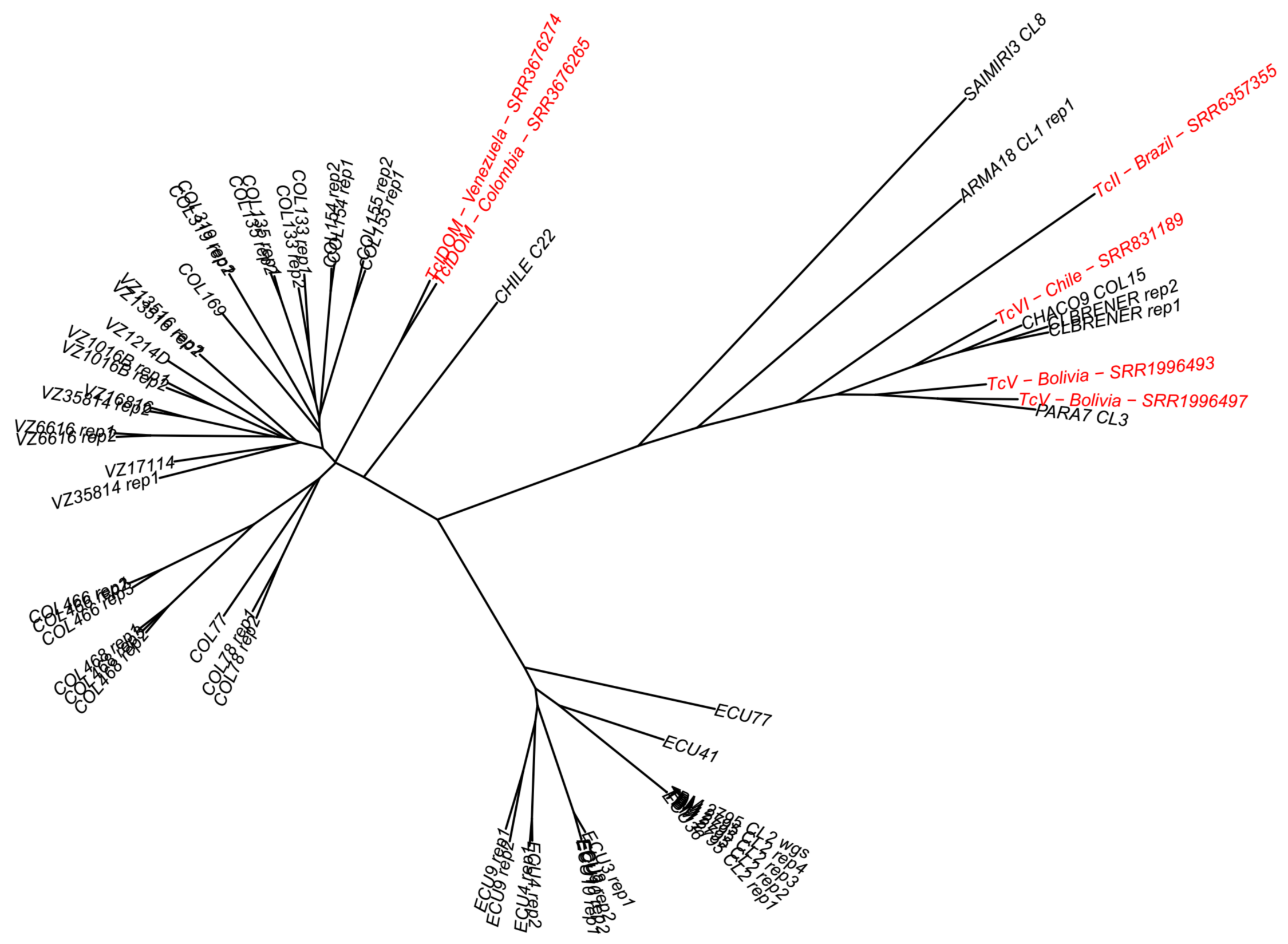

Supplement: S14 Fig — The tree uses seven reference clones (red font with WGS run accessions) in addition to those from Fig 6. We genotyped these clones in silico by subsetting genome-wide variant calls to retain only those occurring within GLST target regions (excluding primer binding sites). Of these, 585 were biallelic and had genotypes called in all individuals. These 585 sites were used for the Euclidean distance matrix of alternate allele counts underlying the tree. The two clones from Colombia and Venezuela represent members of the widespread human-associated ‘TcIDOM’ genotype [71]. The close clustering of these two clones is consistent with previous WGS analyses showing low diversity among geographically disparate TcIDOM isolates [52]. No other TcI samples of the dataset appear to belong to the TcIDOM genotype. The addition of TcII (S11 and Y strain cl. 4) [14], TcIII (strain 231) [112], TcV (92–80 cl. 2) and TcVI (Tulahuen cl. 2) (Washington University School of Medicine) demonstrates limited GLST target differentiation between TcV and TcVI relative to that within TcI and among other DTUs. (TIF) [file pgen.1009170.s014.tif]

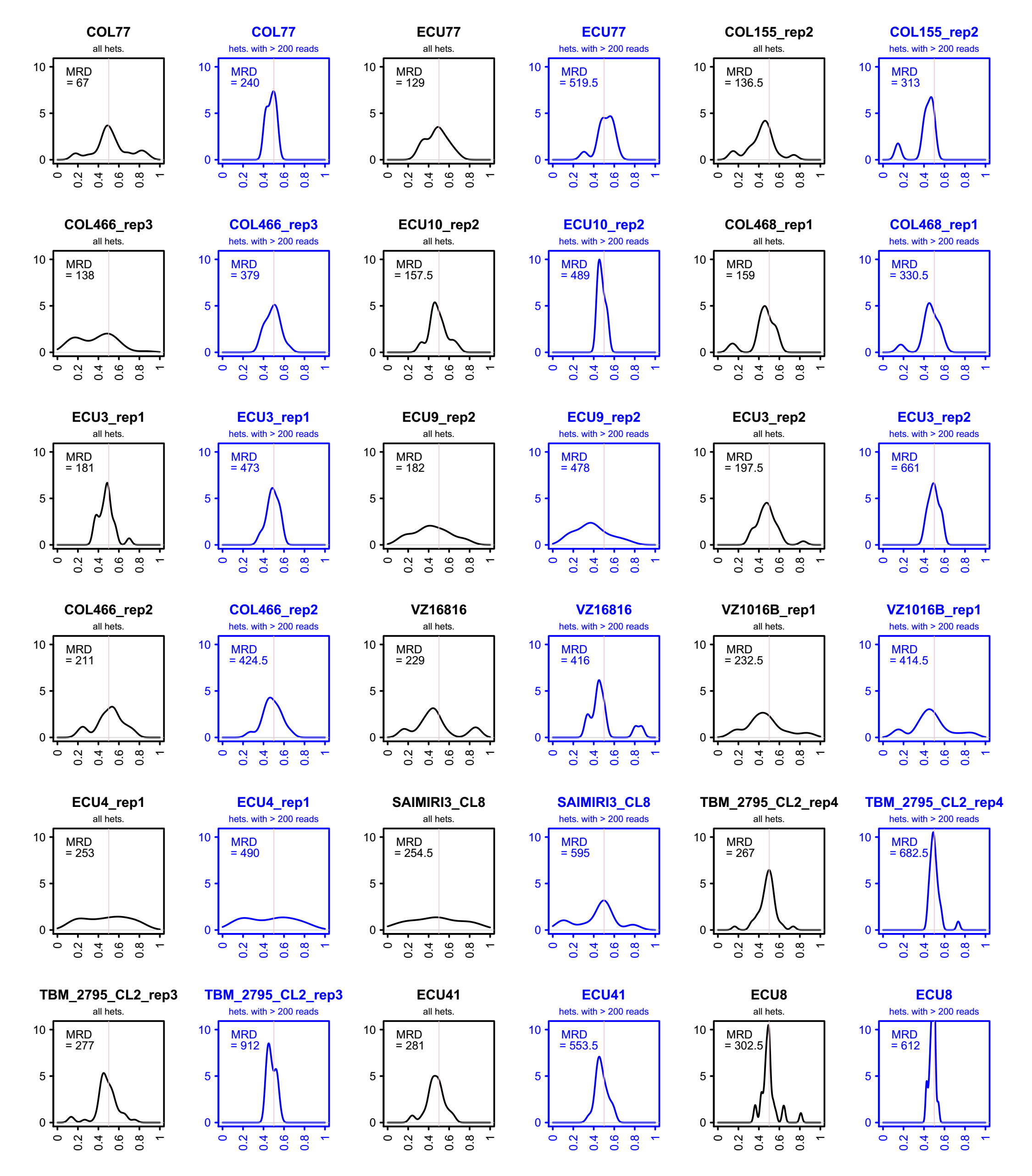

Supplement: S15 Fig — Alternate allele frequency (i.e., the number of non-reference reads divided by the total number of reads representing each genotype) had a mode near 50% in most samples. Distinct and/or additional modes frequently diminished when excluding genotypes represented by ≤ 200 reads (black vs. blue plots). For approximately one third of samples, distinct allele frequency distributions did not change after setting this exclusion. Alternate allele frequency bins are shown on the x-axis and densities are plotted on y. Abbreviations: MRD, median read-depth of heterozygous genotypes; hets., heterozygous genotypes. (TIF) [file pgen.1009170.s015.tif]

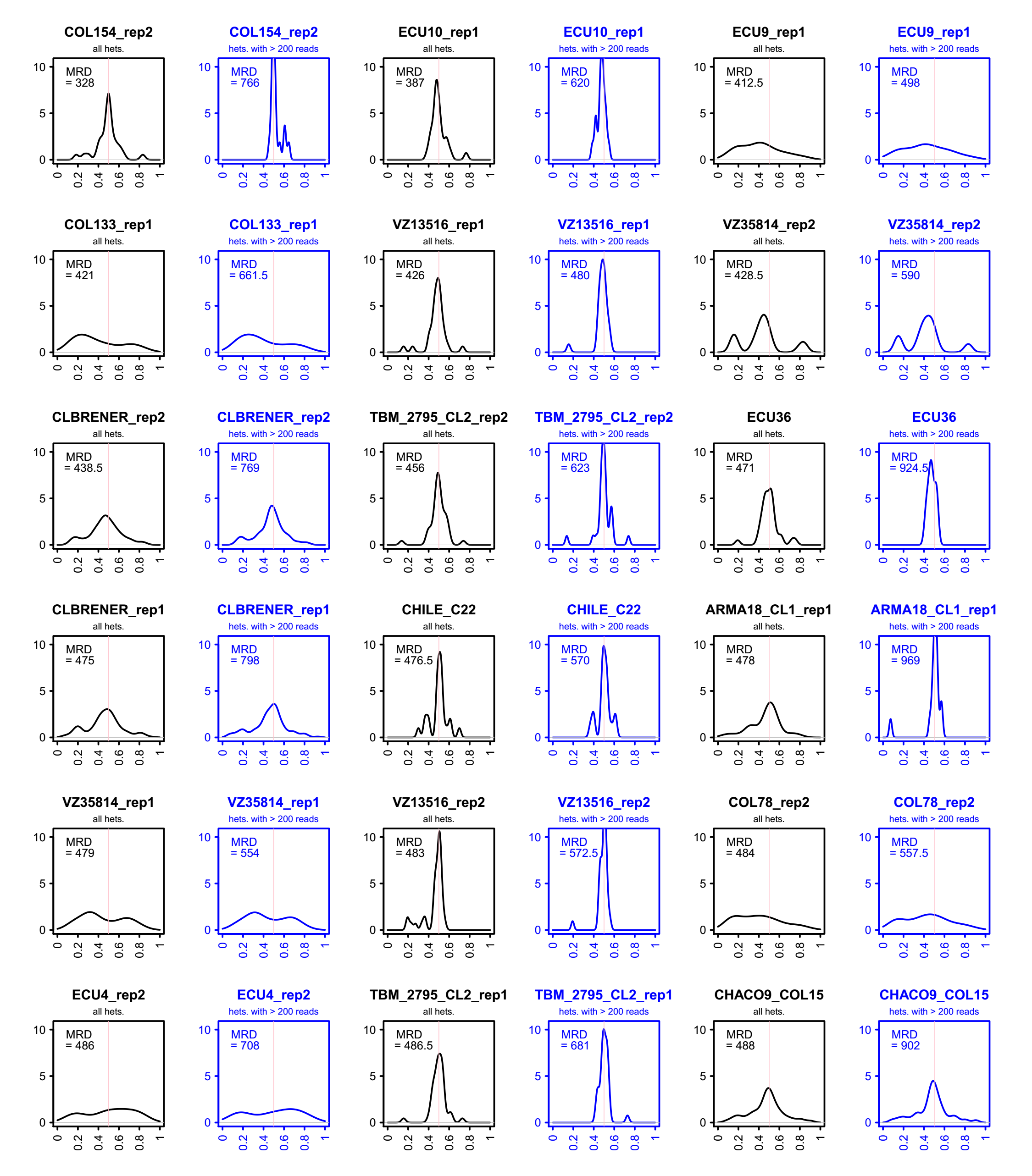

Supplement: S16 Fig — Alternate allele frequency (i.e., the number of non-reference reads divided by the total number of reads representing each genotype) had a mode near 50% in most samples. Distinct and/or additional modes frequently diminished when excluding genotypes represented by ≤ 200 reads (black vs. blue plots). For approximately one third of samples, distinct allele frequency distributions did not change after setting this exclusion. Alternate allele frequency bins are shown on the x-axis and densities are plotted on y. Abbreviations: MRD, median read-depth of heterozygous genotypes; hets., heterozygous genotypes. (TIF) [file pgen.1009170.s016.tif]

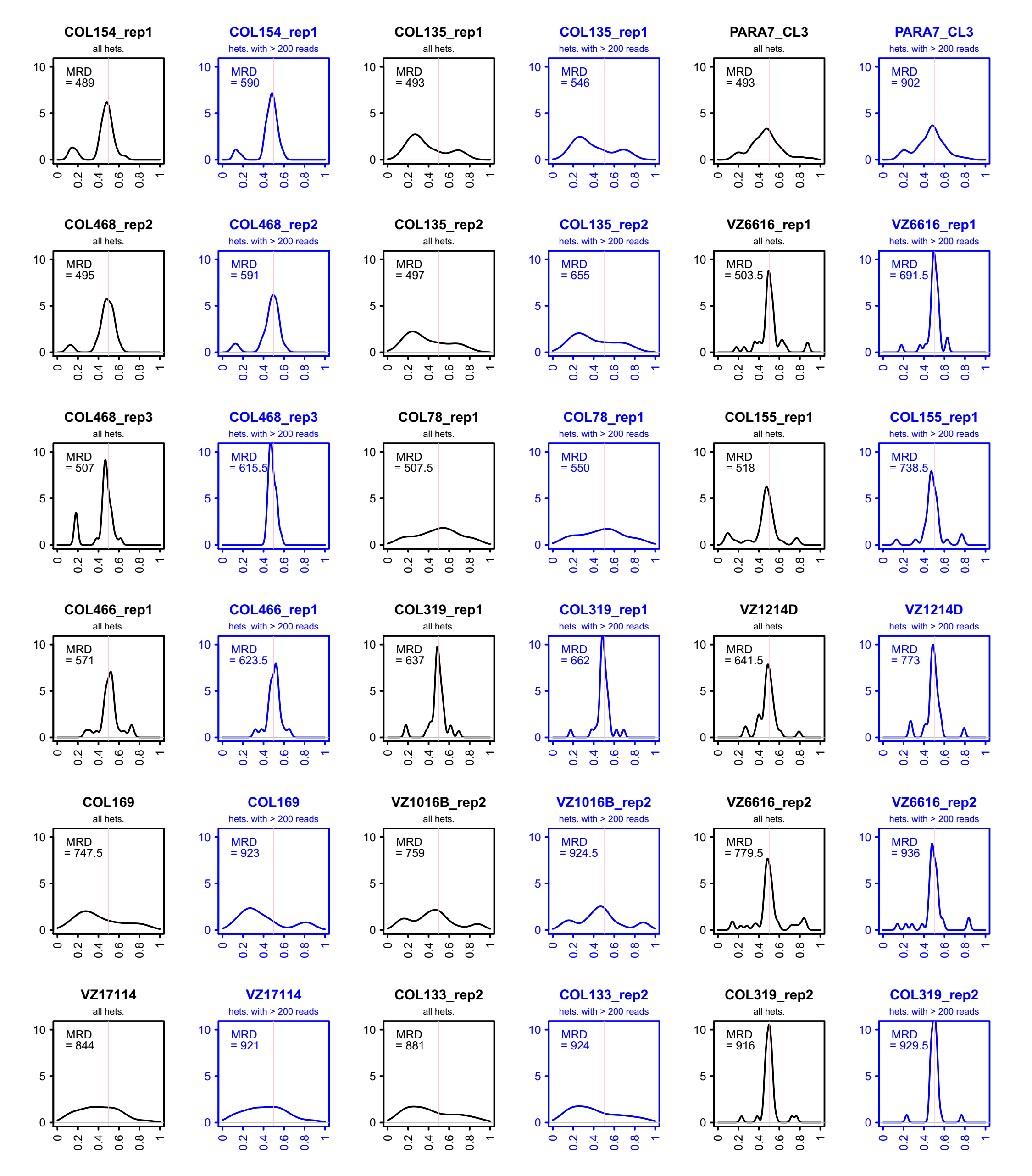

Supplement: S17 Fig — Alternate allele frequency (i.e., the number of non-reference reads divided by the total number of reads representing each genotype) had a mode near 50% in most samples. Distinct and/or additional modes frequently diminished when excluding genotypes represented by ≤ 200 reads (black vs. blue plots). For approximately one third of samples, distinct allele frequency distributions did not change after setting this exclusion. Alternate allele frequency bins are shown on the x-axis and densities are plotted on y. Abbreviations: MRD, median read-depth of heterozygous genotypes; hets., heterozygous genotypes. (TIF) [file pgen.1009170.s017.tif]
