# Supplementary material for: Culture-free genome-wide locus sequence typing (GLST) provides new perspectives on Trypanosoma cruzi dispersal and infection complexity
Source: PLoS Genet. 2020 Dec 16;16(12):e1009170. doi: 10.1371/journal.pgen.1009170 (PMC7743988; doi:10.1371/journal.pgen.1009170)
Supplement: S1 Table — (PDF) [file pgen.1009170.s018.pdf]

S1 Table. Details on *T. cruzi*-infected metagenomic triatomine gut samples from Colombia (COL), Venezuela (VZ) and Ecuador (ECU).

ID	Vector species	Region	Municipality / community	x (EPSG 3786)	y (EPSG 3786)	Ecotope	Year	Reps.
COL77	*Rhodnius pallescens*	Santander Dep.	Lebrija	-8141577.9370	790936.6092	Sylvatic	2015	1
COL78	*Rhodnius sp.*	Santander Dep.	Lebrija	-8141577.9370	790936.6092	Sylvatic	2015	2
COL133	*Rhodnius prolixus*	Casanare Dep.	Paz de Ariporo	-7993997.4220	653950.4247	Domestic	2016	2
COL135	*Rhodnius prolixus*	Casanare Dep.	Paz de Ariporo	-7993997.4220	653950.4247	Domestic	2016	2
COL154	*Rhodnius prolixus*	Casanare Dep.	Tamara	-8024081.7980	648298.0468	Domestic	2016	2
COL155	*Rhodnius prolixus*	Casanare Dep.	Tamara	-8024081.7980	648298.0468	Domestic	2016	2
COL169	*Rhodnius prolixus*	Casanare Dep.	Pore	-8005271.3760	636869.6421	Domestic	2016	1
COL253	*Panstrongylus geniculatus*	Casanare Dep.	Paz de Ariporo	-7993997.4220	653950.4247	Domestic	2016	1
COL319	*Rhodnius prolixus*	Arauca Dep.	Fortul	-7980623.1040	755354.1935	Domestic	2016	2
COL466	*Panstrongylus geniculatus*	Boyacá Dep.	Soata	-8083880.0490	704231.6027	Unknown	2017	3
COL468	*Panstrongylus geniculatus*	Boyacá Dep.	Soata	-8083880.0490	704231.6027	Unknown	2017	3
ECU3	*Rhodnius ecuadoriensis*	Loja Province	Bramaderos	-8875849.2150	-453603.4112	Sylvatic	2009	2
ECU4	*Rhodnius ecuadoriensis*	Loja Province	Bramaderos	-8875849.2150	-453603.4112	Sylvatic	2009	2
ECU8	*Rhodnius ecuadoriensis*	Loja Province	Bramaderos	-8875849.2150	-453603.4112	Sylvatic	2009	1
ECU9	*Rhodnius ecuadoriensis*	Loja Province	Bramaderos	-8875849.2150	-453603.4112	Sylvatic	2009	2
ECU10	*Rhodnius ecuadoriensis*	Loja Province	Bramaderos	-8875849.2150	-453603.4112	Sylvatic	2009	2
ECU36	*Rhodnius ecuadoriensis*	Loja Province	Galápagos	-8832711.9860	-483957.8804	Sylvatic	2009	1
ECU41	*Rhodnius ecuadoriensis*	Loja Province	Guineo	-8899431.9060	-466731.6546	Sylvatic	2009	1
ECU77	*Rhodnius ecuadoriensis*	Loja Province	Jacapo	-8830688.2360	-485500.9341	Sylvatic	2008	1
TBM_2795_CL2	*Panstrongylus chinai*	Loja Province	Bella Maria	-8852271.1950	-466705.6350	Domestic	2009	4
VZ1016B	*Panstrongylus geniculatus*	Met. Caracas	Libertador	-7447967.9080	1167084.6630	Domestic	2016	2
VZ13516	*Panstrongylus geniculatus*	Met. Caracas	Libertador	-7441110.8420	1169154.1140	Domestic	2016	2
VZ35814	*Panstrongylus geniculatus*	Met. Caracas	Libertador	-7450655.1580	1165756.5490	Domestic	2014	2
VZ6616	*Panstrongylus geniculatus*	Met. Caracas	Sucre	-7426686.3980	1163934.1740	Domestic	2016	2
VZ1214D	*Panstrongylus geniculatus*	Met. Caracas	Sucre	-7427396.8230	1166961.1250	Domestic	2014	1
VZ16816	*Panstrongylus geniculatus*	Met. Caracas	Sucre	-7427026.2100	1162328.0720	Domestic	2016	1
VZ17114	*Panstrongylus geniculatus*	Met. Caracas	Sucre	-7426501.1470	1162853.1350	Domestic	2014	1

Abbreviations: Dep., Department; Met. Caracas, Metropolitan District of Caracas; EPSG, European Petroleum Survey Group; reps., technical replicates.
