# Supplementary material for: Culture-free genome-wide locus sequence typing (GLST) provides new perspectives on Trypanosoma cruzi dispersal and infection complexity
Source: PLoS Genet. 2020 Dec 16;16(12):e1009170. doi: 10.1371/journal.pgen.1009170 (PMC7743988; doi:10.1371/journal.pgen.1009170)
Supplement: S2 Table — The 3’ end of each first-round PCR primer is target-specific. The 5’ end of each forward primer contains CS1. The 5’ end of each reverse primer contains CS2. These sequencing primer binding sites are shown in pink. In subsequent barcoding PCR, the reverse primer consists of 5’-CAAGCAGAAGACGGCATACGAGAT*X*TACGGTAGCAGAGACTTGGTCT-3’, where *X* is a unique 10 nt barcode used to label each sample’s sequence reads. The reverse barcoding primer also contains CS2. The forward barcoding primer (5'-AATGATACGGCGACCACCGAGATCTACACTGACGACATGGTTCTA-3') contains CS1 and is the same for all samples. (PDF) [file pgen.1009170.s019.pdf]

S2 Table. GLST primer sequences. The 3’ end of each first-round PCR primer is target-specific. The 5’ end of each forward primer contains CS1. The 5’ end of each reverse primer contains CS2. These sequencing primer binding sites are shown in pink. In subsequent barcoding PCR, the reverse primer consists of 5’-CAAGCAGAAGACGGCATACGAGAT*X*TACGGTAGCAGAGACTTGGTCT-3’, where *X* is a unique 10 nt barcode used to label each sample’s sequence reads. The reverse barcoding primer also contains CS2. The forward barcoding primer (5'-AATGATACGGCGACCACCGAGATCTACACTGACGACATGGTTCTA-3') contains CS1 and is the same for all samples.

ID	Target region	Forward primer sequence (5’ – 3’)	Reverse primer sequence (5’ – 3’)
TC_LOJ_1	chr16:130780-130919	ACACTGACGACATGGTTCTACATGCCAATAACGGTCAAAGTAAACG	TACGGTAGCAGAGACTTGGTCTGCACACGAAGGTACACTCACTTCC
TC_LOJ_2	chr10:534441-534583	ACACTGACGACATGGTTCTACAAGAGTTGTGGCATCCTTGTTCTTG	TACGGTAGCAGAGACTTGGTCTAAACGCCTTCACCTTACTCAGACA
TC_LOJ_4	chr11:368075-368194	ACACTGACGACATGGTTCTACAAGGAGGTGAAACGGATGGTAAAGA	TACGGTAGCAGAGACTTGGTCTTGCGAAGAAGAAGATCAAACTCTCTC
TC_LOJ_5	chr1:2082456-2082586	ACACTGACGACATGGTTCTACAAGCTCAAGGGCTGAAATAGACACA	TACGGTAGCAGAGACTTGGTCTCGTTTAGGCTGGAAAGATGGAAGT
TC_LOJ_6	chr12:1011748-1011869	ACACTGACGACATGGTTCTACACCACTCTATCGTCTACGCATCCTC	TACGGTAGCAGAGACTTGGTCTATCATCTTGAGACACATGCCTTGC
TC_LOJ_8	chr5:515822-515951	ACACTGACGACATGGTTCTACAAATGGAGATGGAGGATATGAAGCA	TACGGTAGCAGAGACTTGGTCTTTTAGACCTCATGTTTCCCGTGTC
TC_LOJ_9	chr1:163164-163296	ACACTGACGACATGGTTCTACACGCTGAGTATCAATTTAAGCGTAGCA	TACGGTAGCAGAGACTTGGTCTACCCATATCCGTCATCCCTATTGT
TC_LOJ_10	chr1:1104374-1104501	ACACTGACGACATGGTTCTACATGCCCTTCACATTTATCCCAAGTA	TACGGTAGCAGAGACTTGGTCTAAATAGCATGGAACTCAGCCAGAA
TC_LOJ_11	chr5:995176-995297	ACACTGACGACATGGTTCTACAGCAACTCCACAAACGACTCAGAAC	TACGGTAGCAGAGACTTGGTCTGATGCTGCCATTTCGTCTTTACTC
TC_LOJ_12	chr14:833083-833213	ACACTGACGACATGGTTCTACACTTGTTGCTAAGTGTCCGTGTGTC	TACGGTAGCAGAGACTTGGTCTGCCTTTATATTGATCGGCTCCTCT
TC_LOJ_13	chr23:560603-560743	ACACTGACGACATGGTTCTACAGTCTTTGATTTCTCGTCCGTACCTT	TACGGTAGCAGAGACTTGGTCTTGCATCTTCTACTTTCTCGGAAGC
TC_LOJ_14	chr19:763581-763703	ACACTGACGACATGGTTCTACAAAGATACAAGAGCACGGTACAAAGGA	TACGGTAGCAGAGACTTGGTCTGTGAAGAGGGATGGATCAACATTC
TC_LOJ_15	chr4:1431898-1432017	ACACTGACGACATGGTTCTACAAGGACTATGCTCAAGACGGGATCT	TACGGTAGCAGAGACTTGGTCTCATCAAGTGGACACAACAGCAACT
TC_LOJ_16	chr16:1168122-1168248	ACACTGACGACATGGTTCTACATACAAACATCAACGCAGAACATGC	TACGGTAGCAGAGACTTGGTCTCACACATCCCGTAACTCAATGGTA
TC_LOJ_19	chr43:177414-177556	ACACTGACGACATGGTTCTACACAGTCCTCCAGTTCTCCAAGTGAT	TACGGTAGCAGAGACTTGGTCTGAGATTGTTCTCTCTGTCCCAACG
TC_LOJ_20	chr26:294140-294261	ACACTGACGACATGGTTCTACAGCACAAGAACGGGTGTACCTTCTA	TACGGTAGCAGAGACTTGGTCTTGTGTCGAGGGAATTGATTACTGC
TC_LOJ_23	chr18:690694-690813	ACACTGACGACATGGTTCTACAAAAGAAACTTCGGGTAGCGACAAC	TACGGTAGCAGAGACTTGGTCTCACCACTTCTGCTAGACCACATCC
TC_LOJ_24	chr1:1993894-1994026	ACACTGACGACATGGTTCTACATTCTACACACTCCGCCTTACGTCT	TACGGTAGCAGAGACTTGGTCTGTCTGCAACGACACATAGATTGGA
TC_LOJ_25	chr36:470603-470728	ACACTGACGACATGGTTCTACAGTGGCTCAGAAGCATGATCGTAAT	TACGGTAGCAGAGACTTGGTCTACCCTTGTAGTCTTCGCAGTCCTC
TC_LOJ_26	chr13:433737-433859	ACACTGACGACATGGTTCTACACAATGGTGATGATGAGGTTAAGCA	TACGGTAGCAGAGACTTGGTCTACGTCCAATACACACAAACACACAG
TC_LOJ_27	chr24:269253-269379	ACACTGACGACATGGTTCTACAGGCGATAAGGAAGAATGGAGAGAA	TACGGTAGCAGAGACTTGGTCTGTCATGTGCTTACGAGAGCCGTAG
TC_LOJ_28	chr27:389665-389794	ACACTGACGACATGGTTCTACAACCACTTCACCATTTGTCTGGTATTC	TACGGTAGCAGAGACTTGGTCTTTTAAGATGGCCGCATACAGTGAG
TC_LOJ_29	chr36:451747-451871	ACACTGACGACATGGTTCTACAGTGTGTTTGAGATTGGGCCTGTAT	TACGGTAGCAGAGACTTGGTCTCACATCAAGTACCTCCGTGTACGA
TC_LOJ_30	chr7:1140939-1141071	ACACTGACGACATGGTTCTACAAGTTGATCGTCTTTCTTCCTTGACC	TACGGTAGCAGAGACTTGGTCTAAATGTTCCTGCGTACACCAAGTC
TC_LOJ_32	chr2:120852-120972	ACACTGACGACATGGTTCTACAAAATGATGTACTGCCTGAACTGGAA	TACGGTAGCAGAGACTTGGTCTGTTCTCCGCCGTATTCTCCTCTAC
TC_LOJ_34	chr16:170448-170597	ACACTGACGACATGGTTCTACAGGAAGAAGGCAGACTAAACAGGATG	TACGGTAGCAGAGACTTGGTCTAGCTTGTCACTGCTCACAGAGTTG

S2 Table (continued)

TC_LOJ_35	chr26:125032-125153	ACACTGACGACATGGTTCTACAGTACGCTACACTGCGAGAGGAATG	TACGGTAGCAGAGACTTGGTCTGCACAACTGAGATTATAGCCAACTCC
TC_LOJ_36	chr5:1012765-1012911	ACACTGACGACATGGTTCTACATCCGTCCCTGTTGTCTTCTCAATA	TACGGTAGCAGAGACTTGGTCTTGAGCAAAGTGTCCTTATTCTTCAGC
TC_LOJ_37	chr1:2889409-2889535	ACACTGACGACATGGTTCTACACAGAGTTCCACGGATAAGTCGTCA	TACGGTAGCAGAGACTTGGTCTACACACTTCCAGATCACTACGAAGC
TC_LOJ_38	chr21:465093-465213	ACACTGACGACATGGTTCTACATGGTTGTAGTCCGTGATCTCTGGT	TACGGTAGCAGAGACTTGGTCTATAACTGGTTCGGGAAGGAAGAAA
TC_LOJ_39	chr1:1160205-1160334	ACACTGACGACATGGTTCTACAACGTCACATTTGTACTGCGAGAGG	TACGGTAGCAGAGACTTGGTCTCCCTTACTTGTCTCCGACTCATTCT
TC_LOJ_40	chr7:1138368-1138496	ACACTGACGACATGGTTCTACAGTCCAAGCCGTTGTCTCTCAATAC	TACGGTAGCAGAGACTTGGTCTTGTTCGTTGTGGTGGAATGTGTAG
TC_LOJ_41	chr1:2693345-2693466	ACACTGACGACATGGTTCTACATGGCTGGTGCAAATGTACTCATATC	TACGGTAGCAGAGACTTGGTCTTAAACAAGTGTGCCATTGCGTATC
TC_LOJ_42	chr10:1016129-1016269	ACACTGACGACATGGTTCTACATACGACTCCCTTTCCACATACGAC	TACGGTAGCAGAGACTTGGTCTATATTGAGCCGAAACACGAAGTACA
TC_LOJ_43	chr1:1956698-1956821	ACACTGACGACATGGTTCTACAGCTCTCATGGGTGGTAGAAGCTAA	TACGGTAGCAGAGACTTGGTCTCCCACTGTCATTATTCAAACTGCTC
TC_LOJ_44	chr3:173883-174019	ACACTGACGACATGGTTCTACAGTCATCATTCTCGGAAACAAAGTAGG	TACGGTAGCAGAGACTTGGTCTGTGTCCATCAGCTCTACAATGCAC
TC_LOJ_45	chr3:174152-174277	ACACTGACGACATGGTTCTACAAGTACGCCACACGACAGTTCAGTT	TACGGTAGCAGAGACTTGGTCTTGAGTAGTTGTGCCCTTCGATGTA
TC_LOJ_46	chr1:1833807-1833948	ACACTGACGACATGGTTCTACAATTCGTGTCATTAGCAGCAGCAAC	TACGGTAGCAGAGACTTGGTCTGACGGTAAATTCTGCGTACACTGC
TC_LOJ_47	chr14:844524-844671	ACACTGACGACATGGTTCTACAAGCAATTCACGGAGTTCACAGATG	TACGGTAGCAGAGACTTGGTCTAGGAGTCACCACAGAAGTCAGAGC
TC_LOJ_48	chr3:1058072-1058196	ACACTGACGACATGGTTCTACAGATAGCACAAACAAGCCAAATGGT	TACGGTAGCAGAGACTTGGTCTGAAAGATACGCCTTCCAATCATCA
TC_LOJ_51	chr12:596775-596914	ACACTGACGACATGGTTCTACAGATTGACATTACGGCGATTCAGAG	TACGGTAGCAGAGACTTGGTCTTGTGGATCTTCTGCCATGATATTG
TC_LOJ_52	chr31:428464-428593	ACACTGACGACATGGTTCTACACCCTCATGGAGACATCTACGAATCT	TACGGTAGCAGAGACTTGGTCTTGAAGAACGAGTGTGCAGGTCATA
TC_LOJ_54	chr2:925727-925855	ACACTGACGACATGGTTCTACAAATGCTAGAGGGCGATAATGAAGAC	TACGGTAGCAGAGACTTGGTCTACCTTTGCCTTGTGTTTACTGCTG
TC_LOJ_55	chr12:306151-306272	ACACTGACGACATGGTTCTACATGGGTCTGCTTGACTGGTTTCTTA	TACGGTAGCAGAGACTTGGTCTGTACGGCGACTCACTTCCAAATAC
TC_LOJ_56	chr21:341510-341636	ACACTGACGACATGGTTCTACAATACTCCTCTGCATTCACCTCCTG	TACGGTAGCAGAGACTTGGTCTGGTTGGTATAACCGAAGGAAATATGG
TC_LOJ_57	chr37:454539-454662	ACACTGACGACATGGTTCTACAGTACGTGAAACGCCCTGACTTTAC	TACGGTAGCAGAGACTTGGTCTTGGATGAACCTCCTTGTAGATGTTG
TC_LOJ_58	chr15:395493-395614	ACACTGACGACATGGTTCTACACTTTGTGACCACCTCCTTGTTATTG	TACGGTAGCAGAGACTTGGTCTAGGTATTTGGCATGTTTGATCTGC
TC_LOJ_59	chr2:856618-856737	ACACTGACGACATGGTTCTACAGCCCGGTTCACAACTTTAGTAGAAA	TACGGTAGCAGAGACTTGGTCTCACCAACACAGCTACGACAACAAC
TC_LOJ_60	chr26:139346-139478	ACACTGACGACATGGTTCTACAGATTATGGTGGTGGTTTCAACACG	TACGGTAGCAGAGACTTGGTCTAAAGTGAATGGCAAATCCTAAGACG
TC_LOJ_61	chr1:1992854-1992995	ACACTGACGACATGGTTCTACAATCTGTTGAGGATGACCGAACACT	TACGGTAGCAGAGACTTGGTCTGAGAAATATCGCCGCACCTTCTAC
TC_LOJ_62	chr1:305886-306012	ACACTGACGACATGGTTCTACATACTCAGGCGTAGAAACAGGCTCA	TACGGTAGCAGAGACTTGGTCTTACCTCCGCTTATCAATGTTGTCC
TC_LOJ_63	chr26:303994-304113	ACACTGACGACATGGTTCTACACATGACAAGCATAAATACAGCGAGAG	TACGGTAGCAGAGACTTGGTCTGAAGGTACAAGCAAGGAGCCATCT
TC_LOJ_64	chr14:889253-889389	ACACTGACGACATGGTTCTACACTTCCCAGACTCATCTTTCTGCTG	TACGGTAGCAGAGACTTGGTCTATTCCCGACTACTTTGGCATGATT
TC_LOJ_67	chr10:143080-143202	ACACTGACGACATGGTTCTACACACTAACTGGGTCAAAGTGTTCTTGC	TACGGTAGCAGAGACTTGGTCTAGCAACTGCGGATACTTGGTCTTC
TC_LOJ_69	chr2:446791-446914	ACACTGACGACATGGTTCTACAGGTAGAAGGTACTCTCATCGGTAGCA	TACGGTAGCAGAGACTTGGTCTCAGAAACAGCTCGCCAGAAATAAA
TC_LOJ_70	chr32:839405-839556	ACACTGACGACATGGTTCTACAGGTGCGTACTGTCTTGGAAGGTTT	TACGGTAGCAGAGACTTGGTCTGTTGACGATCCACGGAAAGATATG
TC_LOJ_71	chr7:179338-179460	ACACTGACGACATGGTTCTACAATGGGAGATCGGGAGTACATGAAG	TACGGTAGCAGAGACTTGGTCTTGAAGAGCCAAATGGGACACTAAT

S2 Table (continued)

TC_LOJ_74	chr1:1413411-1413530	ACACTGACGACATGGTTCTACACAAGATTGTTCCACTGACGAAGACA	TACGGTAGCAGAGACTTGGTCTTTTGAGAGCGTGAAGGAGTACACA
TC_LOJ_75	chr23:504383-504519	ACACTGACGACATGGTTCTACACTTCATCATCTATGCTCCGACGAC	TACGGTAGCAGAGACTTGGTCTTCTGAATGACTGGTTGAAAGACGA
TC_LOJ_76	chr23:505516-505635	ACACTGACGACATGGTTCTACAGTGGACCCAAATGTACTCAGCAAC	TACGGTAGCAGAGACTTGGTCTGAACCTAAGAAACGAAGAACCCTCA
TC_LOJ_80	chr1:2018618-2018750	ACACTGACGACATGGTTCTACAAGTGGACATGGTGACGAAGATGAG	TACGGTAGCAGAGACTTGGTCTGTAGTGCTTCAAACCGCTCAAGAA
TC_LOJ_81	chr37:132370-132499	ACACTGACGACATGGTTCTACAACCGGATGTATTCCTCTCGTGGTA	TACGGTAGCAGAGACTTGGTCTCATGCACTTATCGTCGTCACTTTC
TC_LOJ_82	chr13:741015-741134	ACACTGACGACATGGTTCTACACACAAACCGCTTAGACCCTGAAGT	TACGGTAGCAGAGACTTGGTCTCCAGAAGAAACAATCAATCAACAGC
TC_LOJ_85	chr1:351420-351541	ACACTGACGACATGGTTCTACAAGACTCAATCGCCTTCACGACATA	TACGGTAGCAGAGACTTGGTCTCAGAGGTGTTTATGAGCAAGTACCG
TC_LOJ_86	chr18:746701-746824	ACACTGACGACATGGTTCTACAACCCACTCCAGTAGCATTTCTTCC	TACGGTAGCAGAGACTTGGTCTTTAACTATGGCAATGAGGCAGAGC
TC_LOJ_87	chr37:464692-464819	ACACTGACGACATGGTTCTACACAGATGCTGCCTTGACAGAGATGTA	TACGGTAGCAGAGACTTGGTCTACGAGTGTAGAAGCGAAGATGCTG
TC_LOJ_88	chr16:213322-213477	ACACTGACGACATGGTTCTACAGTAAATAGACACAAGCCATTCCCATC	TACGGTAGCAGAGACTTGGTCTTACTATCACTACCGTGGGCGTCAG
TC_LOJ_89	chr2:121560-121715	ACACTGACGACATGGTTCTACACTCATACCCTTGCTTTGTCATGCT	TACGGTAGCAGAGACTTGGTCTGTTCAGGAGACGGACCACTAGGTT
TC_LOJ_91	chr12:107750-107877	ACACTGACGACATGGTTCTACAGAATGACAACAATGCCCTTTCTTC	TACGGTAGCAGAGACTTGGTCTGTATCTCCATCCATTTCCCAGTGC
TC_LOJ_93	chr27:329031-329151	ACACTGACGACATGGTTCTACATCGTAAAGGTATTGGGCATATTCG	TACGGTAGCAGAGACTTGGTCTCCAGGATCATTCAGCTTAGTCCAG
TC_LOJ_97	chr26:38201-38343	ACACTGACGACATGGTTCTACATTTGAAGAGAAGATGGCCCTGAGT	TACGGTAGCAGAGACTTGGTCTTTGAAGAAAGGATCTGCCTCGTAA
TC_LOJ_99	chr33:297174-297306	ACACTGACGACATGGTTCTACACAAGTTCCTGTTGGACGTGGTAGT	TACGGTAGCAGAGACTTGGTCTAATGTACGCAAGGAGCGACTAGAG
TC_LOJ_100	chr26:479107-479233	ACACTGACGACATGGTTCTACATATTATTTACGAAACGGCGGAGGA	TACGGTAGCAGAGACTTGGTCTAGGAGATGGCTCACTCACTTGAAC
TC_LOJ_102	chr11:853646-853766	ACACTGACGACATGGTTCTACAAGAACAGGAAGTTTGTGACGGTTG	TACGGTAGCAGAGACTTGGTCTATCACCTCTGAAAGAATCGACTGC
TC_LOJ_103	chr13:783091-783210	ACACTGACGACATGGTTCTACAGTACACCCGTCCTTGCAGTATGATT	TACGGTAGCAGAGACTTGGTCTCGCTGAGTTCACGAAGTTATGCTT
TC_LOJ_104	chr15:807734-807870	ACACTGACGACATGGTTCTACACAAGTTCGCAATGTAGGAAAGCTG	TACGGTAGCAGAGACTTGGTCTTATCATGGTGGTCGATGCTGAATA
TC_LOJ_107	chr2:160058-160182	ACACTGACGACATGGTTCTACAGTCATACCTTACCAAACGGCACAG	TACGGTAGCAGAGACTTGGTCTATGTGAACAACCGTACTGGAGGTG
TC_LOJ_108	chr13:664297-664421	ACACTGACGACATGGTTCTACATATCTGTGGTGGCTGTAGATGGTG	TACGGTAGCAGAGACTTGGTCTCGACGACAACAAGGAAGAAGAGGTA
TC_LOJ_109	chr26:419336-419479	ACACTGACGACATGGTTCTACACTTTCGGTGTTACGGTGTACTTCAG	TACGGTAGCAGAGACTTGGTCTTCACTGTTTACAACTACGGCCAGA
TC_LOJ_111	chr41:288290-288430	ACACTGACGACATGGTTCTACACCACGCCACCAGTAACGATAATAA	TACGGTAGCAGAGACTTGGTCTGAAGAAGTGGTACTCTCCCGATCC
TC_LOJ_114	chr5:168922-169061	ACACTGACGACATGGTTCTACATTAGAAACCGTGTAGAGACTTGTCAGC	TACGGTAGCAGAGACTTGGTCTATTACCCTGCACCAAGACACATTC
TC_LOJ_116	chr26:336772-336902	ACACTGACGACATGGTTCTACAGCTGTCTCCAAGAGTCGCAGAATA	TACGGTAGCAGAGACTTGGTCTCATGGATTTCTTTCCAGTGCTTTG
TC_LOJ_117	chr3:965641-965793	ACACTGACGACATGGTTCTACATCCAATCTCTTATCTTTCAGGAGAACG	TACGGTAGCAGAGACTTGGTCTCATACTCAAACGAGGCACGAATCT
TC_LOJ_118	chr15:398374-398497	ACACTGACGACATGGTTCTACACCACAAGTAGGCTGAACCACAAAT	TACGGTAGCAGAGACTTGGTCTGTCAAGCCCTTCGTATCCCTGTTA
TC_LOJ_119	chr1:2137512-2137631	ACACTGACGACATGGTTCTACAGAATCATCAGAGGGTCATTTGCAC	TACGGTAGCAGAGACTTGGTCTAGTACACAACAAAGTTATCGCGGATG
TC_LOJ_120	chr3:196127-196261	ACACTGACGACATGGTTCTACATCATCCTCATCTTCTGGTGGTGAT	TACGGTAGCAGAGACTTGGTCTTGGACTCTCACTTCTGTATCTACTTTGTTG
TC_LOJ_121	chr27:93351-93474	ACACTGACGACATGGTTCTACAACTGCGTTGTATAGCCGAATCACT	TACGGTAGCAGAGACTTGGTCTGACAGGAACACCAAATGTACTGTGAA
TC_LOJ_122	chr36:377593-377718	ACACTGACGACATGGTTCTACACTTTCCTGGGTTCGTTGGTTTAAG	TACGGTAGCAGAGACTTGGTCTCAGGTGTTCCTCGTCAAGCTGTAAT

S2 Table (continued)

TC_LOJ_124	chr10:933564-933686	ACACTGACGACATGGTTCTACATGCAAATACAGAAGATGAGCTACGC	TACGGTAGCAGAGACTTGGTCTTGATTATGAGGAGGAGGATGCAGT
TC_LOJ_125	chr21:539837-539959	ACACTGACGACATGGTTCTACAAAATCTCAGCTACAACAACATCTCTGG	TACGGTAGCAGAGACTTGGTCTTCATCCTTTCCATCGTTCTCACTT
TC_LOJ_126	chr15:908929-909068	ACACTGACGACATGGTTCTACAGGCCTTCTCACTAACTGTCGATCTG	TACGGTAGCAGAGACTTGGTCTACCTTCTTATCACGGAAGAGTATCAGG
TC_LOJ_128	chr11:775649-775772	ACACTGACGACATGGTTCTACAGAAAGAAGCTGAAGAATGGGCAAA	TACGGTAGCAGAGACTTGGTCTGTTGATCCTGGCAATTACACTCGT
TC_LOJ_129	chr18:115349-115471	ACACTGACGACATGGTTCTACAGTGACTTGGCGATTATGATTCGTT	TACGGTAGCAGAGACTTGGTCTCGTTTGTCTTCTCATCCTTCTTCG
TC_LOJ_130	chr9:601749-601872	ACACTGACGACATGGTTCTACATCCCGTTACATCCAATACATCCAA	TACGGTAGCAGAGACTTGGTCTTGCATACACAACAGAGCTAAGTGTCG
TC_LOJ_131	chr9:601909-602028	ACACTGACGACATGGTTCTACAACAAGCAATCCAATTACAACCACAG	TACGGTAGCAGAGACTTGGTCTATTAAAGAAGGTCGCGGCAGTAGA
TC_LOJ_136	chr23:522688-522812	ACACTGACGACATGGTTCTACATTTCAAGCTGCGACTTAATCAACG	TACGGTAGCAGAGACTTGGTCTGATGGAAATGCTTCTTGCACAGTC
TC_LOJ_137	chr16:889485-889604	ACACTGACGACATGGTTCTACACATTTCTGCTGCTTCCTTTGAGAA	TACGGTAGCAGAGACTTGGTCTTCTGATGTTGATCTCTCTTTAACCTACCG
TC_LOJ_138	chr5:1116604-1116723	ACACTGACGACATGGTTCTACACATTTCACCAGAAGTGACAGCAAC	TACGGTAGCAGAGACTTGGTCTGATGAGGGAGAAGCGAATTTGAAC
TC_LOJ_140	chr19:251999-252118	ACACTGACGACATGGTTCTACACCCTCACCTCAATCATATCCACAC	TACGGTAGCAGAGACTTGGTCTGGGACAAGTACGGGAACAGAATAGA
TC_LOJ_141	chr37:317244-317399	ACACTGACGACATGGTTCTACAATTGTGAGAGGATGGGTTCAAATG	TACGGTAGCAGAGACTTGGTCTCCAGTGCATACTTCTGTGTTATGGTAGA
TC_LOJ_142	chr2:327727-327846	ACACTGACGACATGGTTCTACAATGCGGGAGTGTTGTGCATTAGTAT	TACGGTAGCAGAGACTTGGTCTACGGAATACGGGTGGAATAAGAAA
TC_LOJ_144	chr11:235518-235637	ACACTGACGACATGGTTCTACAACGCAGTTGGTCGAGAATTGTATC	TACGGTAGCAGAGACTTGGTCTGAAGGAGAGGTGGTGCAGCTTATC
TC_LOJ_145	chr6:23502-23628	ACACTGACGACATGGTTCTACATTGGCATAAAGGTACGAATCATGG	TACGGTAGCAGAGACTTGGTCTGAACTCACGACCCTGAATAAGACG
TC_LOJ_146	chr27:232849-232974	ACACTGACGACATGGTTCTACACTCAGTATGAACTCCGCTTCCTGT	TACGGTAGCAGAGACTTGGTCTGGATATGTGCTCAAAGTGCCTTGT
TC_LOJ_147	chr4:1219111-1219233	ACACTGACGACATGGTTCTACAAAGCTGAATAGATCGCACAAGCTC	TACGGTAGCAGAGACTTGGTCTTATGCCCTATCCGTGTTTCTTACG
TC_LOJ_152	chr19:553417-553540	ACACTGACGACATGGTTCTACACATAAGGGCAGTGTCATCAACAAA	TACGGTAGCAGAGACTTGGTCTGTATTGCTGGTTGGTTCTCTTCCA
TC_LOJ_154	chr37:156377-156496	ACACTGACGACATGGTTCTACAGTAAGGACCACAAGAGGGAAATGG	TACGGTAGCAGAGACTTGGTCTGCAGAGTAGACAGCATGGAGTGTG
TC_LOJ_156	chr5:627080-627199	ACACTGACGACATGGTTCTACATGGACTACGAGAAGGTTTCATACGAC	TACGGTAGCAGAGACTTGGTCTGCTGTGGAAATGTTGTGATCCTGT
TC_LOJ_157	chr1:1963178-1963304	ACACTGACGACATGGTTCTACATAGAAGAGCGTGTGAAGACTGTGG	TACGGTAGCAGAGACTTGGTCTATGACAACCGCGTCACTTGAATAC
TC_LOJ_158	chr1:1964699-1964825	ACACTGACGACATGGTTCTACACTACACGCATTGTGAGAAACTTGG	TACGGTAGCAGAGACTTGGTCTTGAATTTGTCTGGGATGTGGAAAC
TC_LOJ_159	chr1:1998360-1998510	ACACTGACGACATGGTTCTACAACCGTGCTACTTTCTTCCTTTGGT	TACGGTAGCAGAGACTTGGTCTAATCTTCCTCAATCTCCCTGCTGT
TC_LOJ_160	chr16:738527-738679	ACACTGACGACATGGTTCTACACAGCCACTGTTCAGATCCACAAGT	TACGGTAGCAGAGACTTGGTCTGGCACAAGACCATCAAAGTAGGAC
TC_LOJ_161	chr43:149662-149786	ACACTGACGACATGGTTCTACATGTACCTTTCTGCTTTGTCTTCTTCC	TACGGTAGCAGAGACTTGGTCTTGATGACTATCGCTCCATTCTTCC
TC_LOJ_162	chr16:189968-190097	ACACTGACGACATGGTTCTACAGCTTTGGAGTAGAGCAGATTTGGA	TACGGTAGCAGAGACTTGGTCTCCGAGTTACATTTCTTTGCCTTTG
TC_LOJ_163	chr18:523652-523773	ACACTGACGACATGGTTCTACAGATCGCGTTGTAAGCAAATTCAAG	TACGGTAGCAGAGACTTGGTCTGGCGTAAAGGGCAACTCAAAGTAT
TC_LOJ_165	chr3:169504-169625	ACACTGACGACATGGTTCTACACACGAAAGTCAAACTCCTCCACAA	TACGGTAGCAGAGACTTGGTCTGGTAAATACACGTCCACCGACCTT
TC_LOJ_166	chr3:169646-169792	ACACTGACGACATGGTTCTACAGGCAACGTGGTATGGAATGATAAC	TACGGTAGCAGAGACTTGGTCTTCTGCTCACACAGGACTGAATCTC
TC_LOJ_168	chr28:364521-364659	ACACTGACGACATGGTTCTACACTCGTGGAAGTTTAGTGCTGATCG	TACGGTAGCAGAGACTTGGTCTCGATGATAAAGAAGTCTCCGTACCC
TC_LOJ_169	chr11:721966-722086	ACACTGACGACATGGTTCTACAATGAAACACGTATGCACGATATGC	TACGGTAGCAGAGACTTGGTCTGGCGCTAAATCTGTACGAATACCA

S2 Table (continued)

TC_LOJ_170	chr36:416713-416839	ACACTGACGACATGGTTCTACAGGGAGTACGAGTTTGCAGAGAAGA	TACGGTAGCAGAGACTTGGTCTAGAGGGTTGACATAAGGATGCAGA
TC_LOJ_171	chr2:854454-854583	ACACTGACGACATGGTTCTACAAGCAAGGGCAGTCACAAAGTAACA	TACGGTAGCAGAGACTTGGTCTACTGTGGGTGATACAGGCAAAGAC
TC_LOJ_173	chr19:264153-264279	ACACTGACGACATGGTTCTACACATTGAGAACCACGACTGGCTATT	TACGGTAGCAGAGACTTGGTCTGGACTATGAGATCGACAAGGAGTTTG
TC_LOJ_174	chr18:456154-456275	ACACTGACGACATGGTTCTACAATATCATGGGACTTGCCGGATTAC	TACGGTAGCAGAGACTTGGTCTCAATGTCTGGTTTGGAGGAAGAAG
TC_LOJ_175	chr13:608121-608257	ACACTGACGACATGGTTCTACAACTGACATGGATCATAGCCAATCG	TACGGTAGCAGAGACTTGGTCTCGATAAAGGAACCCAACAAGAACC
TC_LOJ_177	chr7:1112127-1112263	ACACTGACGACATGGTTCTACACTTTGAGAGCTTTGCATCCTTCAC	TACGGTAGCAGAGACTTGGTCTCCGGGACGAGTACACATATACCAA
TC_LOJ_178	chr10:265161-265291	ACACTGACGACATGGTTCTACAGGTATGAGCATCGCCTTATTGATG	TACGGTAGCAGAGACTTGGTCTAAGAGAACCAAATCCCTGAGCAAC
TC_LOJ_180	chr8:851024-851146	ACACTGACGACATGGTTCTACAGACGATGAGGAGTTGGAGGATGTA	TACGGTAGCAGAGACTTGGTCTAGTGTGGCGATAGGTGATTGTGAT
TC_LOJ_181	chr7:987164-987292	ACACTGACGACATGGTTCTACATAGATGTTTGGTCCCATTTGAAGG	TACGGTAGCAGAGACTTGGTCTTGATACCGTCACTATTACCGCTAGAAA
TC_LOJ_182	chr15:497344-497472	ACACTGACGACATGGTTCTACATGTCCAAGACCTTCACATAGTCCA	TACGGTAGCAGAGACTTGGTCTTGGTTACTTTCCAGACAAGGGATG
TC_LOJ_184	chr37:138690-138820	ACACTGACGACATGGTTCTACAAGCTTGGCCTTCAACACATCATTA	TACGGTAGCAGAGACTTGGTCTGCGTCATACTCCCTCACATATCCA
TC_LOJ_185	chr27:387192-387314	ACACTGACGACATGGTTCTACAGGGTGATAGATGCTGTTGCTGAAT	TACGGTAGCAGAGACTTGGTCTTGAGTTTAATGGACCCGAAGGAAC
TC_LOJ_187	chr15:795497-795621	ACACTGACGACATGGTTCTACAGACAAACATTCGACCTTCATCTTCTG	TACGGTAGCAGAGACTTGGTCTTGGTATTTGAGGATCATTCCAGTCA
TC_LOJ_188	chr1:2220221-2220341	ACACTGACGACATGGTTCTACACCAGGTTGTTGGTTGTTATGTGGT	TACGGTAGCAGAGACTTGGTCTGCGGAGATTCACGAAATAGAGGAA
TC_LOJ_191	chr5:703969-704096	ACACTGACGACATGGTTCTACACTATTGGATGGGAACGTGGTACAG	TACGGTAGCAGAGACTTGGTCTGCACAATCTCTGTTGTAAGACTAAACTCCT
TC_LOJ_192	chr37:447759-447878	ACACTGACGACATGGTTCTACACGTATCAAACAGGGCTGGAGACTT	TACGGTAGCAGAGACTTGGTCTATCAAGCTGCAAGAAGAGAACATCC
TC_LOJ_195	chr27:40705-40826	ACACTGACGACATGGTTCTACAATGTTTCCTTGCATGAGTTTGTGG	TACGGTAGCAGAGACTTGGTCTGGAGTCGCCGTAGTATTCCCTTATG
TC_LOJ_197	chr41:298702-298834	ACACTGACGACATGGTTCTACAATTGGGACGGTAGAGCATGTAAGG	TACGGTAGCAGAGACTTGGTCTGCCTGAGTTCCTCCAGTCTTTCTT
TC_LOJ_200	chr37:173415-173536	ACACTGACGACATGGTTCTACACACGAAACTGCCAATGATGACTCT	TACGGTAGCAGAGACTTGGTCTCACCTCCGTCTTTCTTCTCCTTCT
TC_LOJ_201	chr32:855499-855637	ACACTGACGACATGGTTCTACAAAGAGGCGTGTAAGAAGTATGTGGAG	TACGGTAGCAGAGACTTGGTCTTGCAAGTAGTCAGCAATGTCCAGT
TC_LOJ_203	chr25:64845-64984	ACACTGACGACATGGTTCTACAACGCGGATACTAGGGAACATGAGT	TACGGTAGCAGAGACTTGGTCTTTGAGCAGAATACCAAAGCAGTTGT
TC_LOJ_204	chr9:194610-194758	ACACTGACGACATGGTTCTACACTGTTCAAAGTCCATTGTGCTATCC	TACGGTAGCAGAGACTTGGTCTATGACTGCAAGGTATTCCGCTTCT
TC_LOJ_205	chr7:1037003-1037155	ACACTGACGACATGGTTCTACAACAGGGCTTCAGGTGGACATTATT	TACGGTAGCAGAGACTTGGTCTGGTTAAAGGTCGTGGTTGACACAT
TC_LOJ_206	chr19:762223-762346	ACACTGACGACATGGTTCTACAAGCCTTCCCTTTCTACTGGTGGTA	TACGGTAGCAGAGACTTGGTCTTCTGATTTCATACACGTTGCTCCTC
TC_LOJ_209	chr1:2005883-2006014	ACACTGACGACATGGTTCTACATCTTTGAAGGTTCTGGTGTTGGTT	TACGGTAGCAGAGACTTGGTCTTCTCAGGGACGAGGAGACATATAAGA
TC_LOJ_211	chr2:916287-916407	ACACTGACGACATGGTTCTACACTTGATAAACTCTGCGGCTTCCTC	TACGGTAGCAGAGACTTGGTCTCAATGGTACGAACATGATTGACTGTG
TC_LOJ_212	chr44:285730-285879	ACACTGACGACATGGTTCTACAGCTGTCCATATCCGCATCTTCTAA	TACGGTAGCAGAGACTTGGTCTATGTCGTTTCCAAATCAGCACAAC
TC_LOJ_213	chr32:839358-839478	ACACTGACGACATGGTTCTACAGGTGACAAACCCATTCAGCTTACA	TACGGTAGCAGAGACTTGGTCTTACAGCGCCAATCAAATCCACTAC
TC_LOJ_214	chr11:849661-849797	ACACTGACGACATGGTTCTACATTACTACATTGGTGGCGAGACAAAC	TACGGTAGCAGAGACTTGGTCTTCAGACGAAACAGATAGCTCGTGA
TC_LOJ_215	chr10:1052122-1052245	ACACTGACGACATGGTTCTACACAGAGTTCTACAAGGAAGATCGACAAA	TACGGTAGCAGAGACTTGGTCTTTAATGATGGGTGGAAGTGAGAGG
TC_LOJ_217	chr1:2773733-2773861	ACACTGACGACATGGTTCTACAAAACTTATGGCGTACAACAGGGAGT	TACGGTAGCAGAGACTTGGTCTCGATAACGACGATGAAGATGATGA

S2 Table (continued)

TC_LOJ_219	chr26:38066-38187	ACACTGACGACATGGTTCTACAGTTGATGTGGATAGGCTTGACTACTTTC	TACGGTAGCAGAGACTTGGTCTTCACCTTCGTAGCACAATACCTTACA
TC_LOJ_220	chr14:923562-923682	ACACTGACGACATGGTTCTACATCGGGTAAATGTCTAACGGAGAAA	TACGGTAGCAGAGACTTGGTCTCCAGATCCAGTGATTCGTCTTGTT
TC_LOJ_221	chr11:868950-869070	ACACTGACGACATGGTTCTACAGCTTCACAGCTATCGAGGTGTATTG	TACGGTAGCAGAGACTTGGTCTCCAGGAGTTTAGTTACAACAGACGAGA
TC_LOJ_223	chr27:96137-96258	ACACTGACGACATGGTTCTACACAAGCGCACCCTAATAAGAAATTG	TACGGTAGCAGAGACTTGGTCTCAACAAAGAGCTTCAAATGGTGTG
TC_LOJ_224	chr1:2775484-2775623	ACACTGACGACATGGTTCTACAGGTGTGTACGGATGACTGCTACTTACTT	TACGGTAGCAGAGACTTGGTCTCAACAAGGACAAAGACAACCACAA
TC_LOJ_225	chr15:246311-246435	ACACTGACGACATGGTTCTACACGTGAAAGATACGGCTGACACATA	TACGGTAGCAGAGACTTGGTCTGTAGTGCGTGTTGCTCCTGTTGTT
TC_LOJ_227	chr27:116142-116263	ACACTGACGACATGGTTCTACAATGAGGAGGAGGAGAAATGGAAAC	TACGGTAGCAGAGACTTGGTCTGTCGATGACACAGTCCAGACACTC
TC_LOJ_228	chr5:1147485-1147616	ACACTGACGACATGGTTCTACAACAGTGCAGTCGTACTTTCGCATT	TACGGTAGCAGAGACTTGGTCTTGTTGACTACTTTGACGGAAATCGT
TC_LOJ_229	chr5:1148049-1148168	ACACTGACGACATGGTTCTACAAGTGGCTTGGCAGATTTCTTCTGT	TACGGTAGCAGAGACTTGGTCTTGACAGTTTAGAGAGCGTTGTAGTGAAAG
TC_LOJ_230	chr15:926778-926915	ACACTGACGACATGGTTCTACAATTCTGCCTGCGACAGTAGTTCTC	TACGGTAGCAGAGACTTGGTCTCCATTCTTCGTGAAATTGAGGTTG
TC_LOJ_231	chr1:2138077-2138196	ACACTGACGACATGGTTCTACAGGCAGACTCCAGATACTGACGAAT	TACGGTAGCAGAGACTTGGTCTCCACAACTCCTTGACGACTTTCTT
TC_LOJ_232	chr5:191326-191447	ACACTGACGACATGGTTCTACAACATCCTGACCCTTGGCTTTAGAC	TACGGTAGCAGAGACTTGGTCTGGTTAGAGAGAACATTACGACGGAGA
TC_LOJ_234	chr10:715504-715626	ACACTGACGACATGGTTCTACAAGTAAGCCTGTTGCTTTGGAAACTC	TACGGTAGCAGAGACTTGGTCTTCAACCCAGACGAAAGTCTAGTGG
TC_LOJ_235	chr15:197505-197635	ACACTGACGACATGGTTCTACATCGTCAATTTCCCGTAGGATACTTT	TACGGTAGCAGAGACTTGGTCTCAGGAGGAGGGTGAACTGATAATG
TC_LOJ_236	chr11:235245-235379	ACACTGACGACATGGTTCTACAATCTTTACCATGCACCTCCACAAC	TACGGTAGCAGAGACTTGGTCTGGTCTCACCACGTATCACGAGAAG
TC_LOJ_237	chr9:134209-134328	ACACTGACGACATGGTTCTACACTCTTCACGCCAATACATTCCTTG	TACGGTAGCAGAGACTTGGTCTCCAGCTACAACTGCAAACAAATACAC
TC_LOJ_238	chr21:322787-322911	ACACTGACGACATGGTTCTACATCAGGGTAGATTCATCAGGCAGAG	TACGGTAGCAGAGACTTGGTCTTATCAACAATGCTCGACACCCACT
TC_LOJ_239	chr44:237246-237373	ACACTGACGACATGGTTCTACAATTTATGCCCGCAAACCAGATAAC	TACGGTAGCAGAGACTTGGTCTCGAGGCAATTCGTATAATGTCTTCA
TC_LOJ_242	chr31:92921-93071	ACACTGACGACATGGTTCTACAATTGAAGTATCGCCAGAACAGCAT	TACGGTAGCAGAGACTTGGTCTGTGTTGCTTGGAGTAAGGCACTCT
TC_LOJ_243	chr21:288200-288319	ACACTGACGACATGGTTCTACACGGTCAGGATCGTTATAGTTTGGTAG	TACGGTAGCAGAGACTTGGTCTAGACACTTTGTATCGTATGCGTCGT
TC_LOJ_244	chr18:566462-566592	ACACTGACGACATGGTTCTACAATTATCTCGTGAGTTTGGCGGAAT	TACGGTAGCAGAGACTTGGTCTCAGAACCGTCTTGTCCTTCACTTC
TC_LOJ_245	chr3:1209990-1210114	ACACTGACGACATGGTTCTACAGGATCGACGTATGGGACGTATTTC	TACGGTAGCAGAGACTTGGTCTTTGAAGGACTGGAGCAAGACAAGT
TC_LOJ_249	chr10:1031977-1032097	ACACTGACGACATGGTTCTACAAAGCTCAGTGTTCAAAGTGCCATC	TACGGTAGCAGAGACTTGGTCTTTTCCTTGTTATCGGCTGTGAGAA
TC_LOJ_250	chr21:505080-505199	ACACTGACGACATGGTTCTACAGTTCTCCGTTACTTTCCGACACAG	TACGGTAGCAGAGACTTGGTCTTGCCATGTTACCCATAAACCACTT
TC_LOJ_251	chr5:743274-743396	ACACTGACGACATGGTTCTACACTAGGGATAGTGTCTCAACATTGGCTATAA	TACGGTAGCAGAGACTTGGTCTCACCCTTTAACTTTGAACGAACACG
TC_LOJ_252	chr36:237339-237479	ACACTGACGACATGGTTCTACATTAGAGCTTCGTATCGGCATGTTG	TACGGTAGCAGAGACTTGGTCTCACTTCATACATTTCCTCCAGAGACC
TC_LOJ_253	chr3:240382-240505	ACACTGACGACATGGTTCTACACCACTACCATTACCCGTGTCGTTA	TACGGTAGCAGAGACTTGGTCTCGCAGTCCTTGCTTAACCTCATTT
TC_LOJ_255	chr27:388555-388675	ACACTGACGACATGGTTCTACAGTTATTTGTATCCGTATCTTGCTGTCG	TACGGTAGCAGAGACTTGGTCTAGTATCACCTGGAGGACCGTGAAG
TC_LOJ_256	chr39:221720-221854	ACACTGACGACATGGTTCTACAAACTGACCGGAAGTGAGATTGATG	TACGGTAGCAGAGACTTGGTCTGGGCGGCGTCGTAGTATAAATAAG
TC_LOJ_257	chr5:992280-992407	ACACTGACGACATGGTTCTACACCTTTATTACGCTTCGGCAAGTACA	TACGGTAGCAGAGACTTGGTCTTTCCACGCAAACAATCAGTATCAG
TC_LOJ_259	chr32:837402-837557	ACACTGACGACATGGTTCTACAACTCTACACAAAGGCGTCAGAGATG	TACGGTAGCAGAGACTTGGTCTCCTGCAAGATCAATAAGGTTCAGC

S2 Table (continued)

TC_LOJ_260	chr4:1353006-1353141	ACACTGACGACATGGTTCTACATGGTACTTGTTCAGCTCGGAAATC	TACGGTAGCAGAGACTTGGTCTCAAAGGCAGAGGAATGTTCAAAGA
TC_LOJ_262	chr1:2151183-2151303	ACACTGACGACATGGTTCTACACCGTAGTTGCGGTACGAATAAGTG	TACGGTAGCAGAGACTTGGTCTACTGGGAACGTGTATTAGGTATGGAGT
TC_LOJ_264	chr18:649186-649316	ACACTGACGACATGGTTCTACAGTGGAGGCGAAGAAGAAGTTTACA	TACGGTAGCAGAGACTTGGTCTAATAGAAACGGCATTCCATAAGCAC
TC_LOJ_265	chr27:343910-344029	ACACTGACGACATGGTTCTACAGTGCATCATATTCGATAGGGAGATGT	TACGGTAGCAGAGACTTGGTCTTATTACAGCATTGACCGTGTCTTCC
TC_LOJ_266	chr1:2205081-2205202	ACACTGACGACATGGTTCTACACTACGAAGTGCCTTAACTGCCTCA	TACGGTAGCAGAGACTTGGTCTATTCTATGTGCGTTTGGGTTTCAG
TC_LOJ_267	chr26:405401-405543	ACACTGACGACATGGTTCTACATTGCTTTCGATGGAGATAGACCTTT	TACGGTAGCAGAGACTTGGTCTGCGGAGATGTCTGATTTAGGAATTG
TC_LOJ_268	chr26:302445-302583	ACACTGACGACATGGTTCTACACGTAGTCAAACGGACTGAAGTACACA	TACGGTAGCAGAGACTTGGTCTGAGGAGGCAGTGGAGGTGTTAAAT
TC_LOJ_269	chr5:495739-495879	ACACTGACGACATGGTTCTACATCTTTATGACAAGTGCAACCAAAGC	TACGGTAGCAGAGACTTGGTCTCGTGATACTCCACCGTCTCAATCT
TC_LOJ_271	chr2:323827-323957	ACACTGACGACATGGTTCTACAGTGGGTTTCATCTCTCGTTTATGC	TACGGTAGCAGAGACTTGGTCTACCCTTGTCCATGTGTCTTGTAGC
TC_LOJ_273	chr1:2140290-2140430	ACACTGACGACATGGTTCTACACAATGGCACCAAGATAATAGTACAGGA	TACGGTAGCAGAGACTTGGTCTTGCAGAACCATCGTGAGAACTTTA
TC_LOJ_274	chr21:239185-239310	ACACTGACGACATGGTTCTACAAACAAGGTGAAGAAGAGCCATCAG	TACGGTAGCAGAGACTTGGTCTAAGGTGGAGGAGTTTGAACAGTACG
TC_LOJ_275	chr39:50470-50598	ACACTGACGACATGGTTCTACACTGCTCCTGATACTGCACAAACTG	TACGGTAGCAGAGACTTGGTCTGGTGCCTACAATGACTCCGTACAC
TC_LOJ_276	chr1:2694842-2694979	ACACTGACGACATGGTTCTACATTACACATTGCAGGGCAGCATATT	TACGGTAGCAGAGACTTGGTCTGTCTTTGTTCGTCATGTCAGCGTA
TC_LOJ_277	chr4:1382610-1382749	ACACTGACGACATGGTTCTACATAGCATCTTAATCAGCTCGGGAGA	TACGGTAGCAGAGACTTGGTCTGACGAACAAATGGAGAATCAGACG
TC_LOJ_278	chr2:164952-165077	ACACTGACGACATGGTTCTACAGGTCATTCACGCCAGTTCATACAT	TACGGTAGCAGAGACTTGGTCTACGGCCTTCTTCATAATCTCCATAA
TC_LOJ_279	chr9:400076-400197	ACACTGACGACATGGTTCTACACGAGACAGGGATGGACTCTTCAAT	TACGGTAGCAGAGACTTGGTCTGTTACGATGGCCTTGAGTGTGAGA
TC_LOJ_280	chr21:505326-505445	ACACTGACGACATGGTTCTACAGATTGCTACGTGAAGACGTGGAAG	TACGGTAGCAGAGACTTGGTCTGAGCGTATCGTACAGGCCAAAGTA
TC_LOJ_281	chr10:735827-735952	ACACTGACGACATGGTTCTACACAACGCATTTGGATTGCCTACTAA	TACGGTAGCAGAGACTTGGTCTAAACGTCTTGGTCTGTACGAGGAG
TC_LOJ_282	chr37:470203-470342	ACACTGACGACATGGTTCTACACTACTCAAGGAACCAGGCGTATTG	TACGGTAGCAGAGACTTGGTCTAACGTCCCACCAAGAATAATGAGC
TC_LOJ_283	chr12:561576-561706	ACACTGACGACATGGTTCTACACAGAAGGAGAAGACATTGGAACTCA	TACGGTAGCAGAGACTTGGTCTTCTTTGCCACTATCAAGCACCAAC
TC_LOJ_285	chr31:132291-132424	ACACTGACGACATGGTTCTACACATTGACCTTGCCACAGAAGTGTA	TACGGTAGCAGAGACTTGGTCTTGGCCTTATTCACATACTCCACAAG
TC_LOJ_286	chr15:941983-942118	ACACTGACGACATGGTTCTACAGGCGTATCCACCACAAGAGTAGAA	TACGGTAGCAGAGACTTGGTCTGGATGCCAGATTACGTGAAAGAAA
