# Supplementary material for: Culture-free genome-wide locus sequence typing (GLST) provides new perspectives on Trypanosoma cruzi dispersal and infection complexity
Source: PLoS Genet. 2020 Dec 16;16(12):e1009170. doi: 10.1371/journal.pgen.1009170 (PMC7743988; doi:10.1371/journal.pgen.1009170)
Supplement: S3 Table — T. cruzi samples/clones are listed in ascending order of total number of heterozygous genotypes (i.e., heterozygosity count in column 2). High heterozygosity counts in PARA7_CL3, CLBRENER and CHACO9_COL15 is consistent with TcV and TcVI originating via hybridization between progenitors of TcII and TcIII [79]. In fact, all 194–210 heterozygous sites found in these three clones match sites at which TcII (variants called from publicly available WGS reads (run accession SRR6357355) [14]) differs from ARMA18_CL1 (TcIII). Heterozygosity per polymorphic genotype refers to the number of heterozygous genotypes divided by the total number of polymorphic genotypes per sample/clone. The fifth column indicates the proportion of all GLST sites (26,042 bp) at which reads representing > 2 alleles were detected with GATK [48] ‘HaplotypeCaller’ algorithm set to ‘-ploidy 4’. This setting allows for tri- and tetra-allelic genotype calls. None occurred. (PDF) [file pgen.1009170.s020.pdf]

S3 Table. Heterozygosity and allele frequency metrics. *T. cruzi* samples/clones are listed in ascending order of total number of heterozygous genotypes (i.e., heterozygosity count in column 2). High heterozygosity counts in PARA7_CL3, CLBRENER and CHACO9_COL15 is consistent with TcV and TcVI originating via hybridization between progenitors of TcII and TcIII [79]. In fact, all 194 – 210 heterozygous sites found in these three clones match sites at which TcII (variants called from publicly available WGS reads (run accession SRR6357355) [14]) differs from ARMA18_CL1 (TcIII). Heterozygosity per polymorphic genotype refers to the number of heterozygous genotypes divided by the total number of polymorphic genotypes per sample/clone. The fifth column indicates the proportion of all GLST sites (26,042 bp) at which reads representing > 2 alleles were detected with GATK [48] 'HaplotypeCaller' algorithm set to '-ploidy 4'. This setting allows for tri- and tetra-allelic genotype calls. None occurred.

Sample / replicate	Heterozygosity count	Heterozygosity per polymorphic genotype	AAFH at biallelic sites	Prop. sites with reads representing > 2 alleles
SAIMIRI3_CL8	19	0.09	0.48	0.0005
ECU10_rep2	22	0.29	0.48	0.0000
ECU3_rep1	23	0.30	0.48	0.0001
ARMA18_CL1_rep1	25	0.12	0.50	0.0008
CHILE_C22	25	0.44	0.50	0.0000
ECU77	25	0.31	0.48	0.0002
VZ16816	26	0.48	0.45	0.0000
COL77	27	0.49	0.51	0.0000
COL466_rep1	28	0.41	0.52	0.0002
COL468_rep2	28	0.42	0.49	0.0001
ECU10_rep1	28	0.31	0.48	0.0003
ECU3_rep2	28	0.32	0.48	0.0002
ECU8	28	0.32	0.49	0.0001
COL466_rep2	29	0.43	0.52	0.0000
COL154_rep2	30	0.55	0.50	0.0000
COL468_rep1	30	0.49	0.47	0.0001
VZ35814_rep2	31	0.50	0.43	0.0002
COL468_rep3	33	0.46	0.47	0.0001
VZ1214D	33	0.54	0.49	0.0001
VZ13516_rep1	33	0.53	0.48	0.0000
COL154_rep1	34	0.53	0.48	0.0004
ECU36	34	0.37	0.50	0.0003
COL319_rep1	35	0.52	0.49	0.0002
COL319_rep2	36	0.50	0.50	0.0002
ECU41	36	0.41	0.47	0.0001
VZ13516_rep2	38	0.54	0.49	0.0002
COL155_rep2	39	0.61	0.44	0.0000
ECU9_rep2	39	0.43	0.42	0.0002
COL155_rep1	40	0.60	0.47	0.0000
TBM_2795_CL2_rep2	41	0.39	0.49	0.0004
TBM_2795_CL2_rep4	41	0.39	0.49	0.0002
VZ6616_rep1	41	0.54	0.50	0.0001
TBM_2795_CL2_rep1	42	0.39	0.50	0.0003
COL466_rep3	43	0.53	0.40	0.0003

S3 Table (continued)

TBM_2795_CL2_rep3	43	0.41	0.48	0.0003
VZ6616_rep2	45	0.56	0.50	0.0003
COL133_rep1	48	0.71	0.34	0.0001
VZ1016B_rep1	48	0.70	0.44	0.0001
COL133_rep2	49	0.71	0.35	0.0001
VZ17114	50	0.68	0.41	0.0002
COL169	53	0.72	0.35	0.0002
COL78_rep1	53	0.69	0.50	0.0000
ECU9_rep1	54	0.50	0.39	0.0004
COL135_rep2	55	0.71	0.32	0.0003
ECU4_rep1	55	0.53	0.48	0.0001
COL135_rep1	57	0.72	0.31	0.0000
ECU4_rep2	58	0.52	0.51	0.0003
VZ1016B_rep2	58	0.71	0.45	0.0004
VZ35814_rep1	59	0.70	0.35	0.0000
COL78_rep2	71	0.76	0.38	0.0002
PARA7_CL3	194	0.58	0.47	0.0011
CLBRENER_rep1	197	0.59	0.47	0.0008
CLBRENER_rep2	204	0.58	0.47	0.0007
CHACO9_COL15	210	0.60	0.48	0.0007

Abbreviations: AAFH, median alternate allele frequency of heterozygous genotypes; prop., proportion of.
