# Supplementary material for: Culture-free genome-wide locus sequence typing (GLST) provides new perspectives on Trypanosoma cruzi dispersal and infection complexity
Source: PLoS Genet. 2020 Dec 16;16(12):e1009170. doi: 10.1371/journal.pgen.1009170 (PMC7743988; doi:10.1371/journal.pgen.1009170)
Supplement: S4 Table — Green dots indicate items/costs related to first-round PCR and clean-up. Blue dots indicate items/costs related to barcoding PCR and clean-up. The cost summary does not consider qPCR materials because we applied qPCR only for purposes of method development. (PDF) [file pgen.1009170.s021.pdf]

**S4 Table. Summary of GLST library preparation and sequencing costs.** Green dots indicate items/costs related to first-round PCR and clean-up. Blue dots indicate items/costs related to barcoding PCR and clean-up. The cost summary does not consider qPCR materials because we applied qPCR only for purposes of method development.

| | Item | Availability (quantity / price) | Quantity for 100 samples | Cost for 100 samples | Comment |
|---|---|---|---|---|---|
| Library preparation | 200 GLST primer primer pairs (EUG) 🟢 | 60.90 ml / 1508.88 £ | 25 pmol | 1.26 £ | 18,861 bases purchased salt-free at 0.08 £ / base; primers delivered at 200 µM in 150 µl |
| | Q5 High-Fidelity 2X Master Mix (NEB) 🟢 | 2.5 ml / 106.75 £ | 500 µl | 21.35 £ | |
| | UltraPure Agarose (Invitrogen) 🟢 | 100 g / 124.00 £ | 15.6 g | 19.34 £ | 13 agarose gels (0.8%) to visualize 100 samples, separated by empty lanes |
| | 100 bp DNA Ladder* (NEB) 🟢 | 50 µg / 34.50 £ | 13 µg | 8.97 £ | 0.5 µg ladder at left and right margins of each gel |
| | 6X Gel Loading Dye (NEB) 🟢 | 1 ml free with ladder* | 226 µl | 0.00 £ | 2 µl dye for each sample/ladder lane |
| | PureLink Quick Gel Extraction Kit (Invitrogen) 🟢 | 3 x 50 units / 143.64 £ | 100 units | 95.76 £ | |
| | SYBR Safe (Invitrogen) 🟢 | 400 µl / 62.78 £ | 60 µl | 9.42 £ | |
| | Miscellaneous 🟢 | n/a | n/a | 50.00 £ | Pipette tips, vials, blades, etc. |
| | Barcoded reverse primer (EUG) 🔵 | 0.02 µmol / 49.95 £ | 0.8 nmol | 2.00 £ | Primers purified by manufacturer using high performance liquid chromatography |
| | Universal forward primer (EUG) 🔵 | 0.02 µmol / 49.95 £ | 0.8 nmol | 2.00 £ | Primers purified by manufacturer using high performance liquid chromatography |
| | Q5 High-Fidelity 2X Master Mix (NEB) 🔵 | see above | 1 ml | 42.70 £ | |
| | Nuclease-free $dH_2O$ (Qiagen) 🔵 | 1000 ml / 35.68 £ | 540 µl | 19.27 £ | |
| | Qubit assay tubes (Invitrogen) 🔵 | 500 tubes / 51.50 £ | 102 tubes | 10.51 £ | |
| | Qubit dsDNA HS Assay Kit (Invitrogen) 🔵 | 100 assay kit / 66.25 £ | 100 assays | 66.25 £ | |
| | UltraPure Agarose (Invitrogen) 🔵 | see above | 1.2 g | 1.49 £ | Only one agarose gel (0.8%) is needed because samples have been pooled |
| | 100 bp DNA Ladder (NEB) 🔵 | see above | 1 µg | 0.69 £ | 0.5 µg ladder at left and right margins of the gel |
| | 6X Gel Loading Dye (NEB) 🔵 | see above | 9 µl | 0.00 £ | 7 µl dye for sample (pool) lane, 2 µl for each ladder lane |
| | PureLink Quick Gel Extraction Kit (Invitrogen) 🔵 | see above | 1 unit | 0.96 £ | Only one unit is needed because samples have been pooled |
| | SYBR Safe (Invitrogen) 🔵 | see above | 10 µl | 1.57 £ | |
| | Miscellaneous 🔵 | n/a | n/a | 50.00 £ | Pipette tips, vials, blades, etc. |
| | | | | Total library preparation cost for 100 samples: 256.41 £ | ~ 3.15 $ per sample |

| | Item | Availability (quantity / price) | Quantity for 100 samples | Cost for 100 samples | Comment |
|---|---|---|---|---|---|
| Sequencing | Illumina Reagent Kit v2 Micro | 1 cartridge / 390.00 £ | 1 cartridge | 390.00 £ | As listed at https://emea.illumina.com (March 2020) |
| | 300-cycle Illumina MiSeq | 1 run / 400.00 £ | 1 run | 400.00 £ | Costs for quality control, data storage, etc. vary considerably among providers |
| | | | | Total sequencing cost for 100 samples: 790.00 £ | ~ 9.72 $ per sample; 70x MGRD expected based on 125x MGRD for 56 samples in run 2 |

Abbreviations: EUG, Eurofins Genomics; NEB, New England Biolabs; MGRD, median genotype read-depth.
